# New Glucosamine-Based TLR4 Agonists: Design, Synthesis, Mechanism of Action, and In Vivo Activity as Vaccine Adjuvants

**DOI:** 10.1021/acs.jmedchem.2c01998

**Published:** 2023-02-02

**Authors:** Alessio Romerio, Nicole Gotri, Ana Rita Franco, Valentina Artusa, Mohammed Monsoor Shaik, Samuel T. Pasco, Unai Atxabal, Alejandra Matamoros-Recio, Marina Mínguez-Toral, Juan Diego Zalamea, Antonio Franconetti, Nicola G. A. Abrescia, Jesus Jimenez-Barbero, Juan Anguita, Sonsoles Martín-Santamaría, Francesco Peri

**Affiliations:** † Department of Biotechnology and Biosciences, University of Milano-Bicocca, Piazza della Scienza, 2, 20126 Milano, Italy; ‡ Center for Cooperative Research in Biosciences (CIC bioGUNE), Basque Research and Technology Alliance (BRTA), 48160 Derio, Bizkaia, Spain; § Centro de Investigaciones Biológicas Margarita Salas CSIC, C/Ramiro de Maeztu, 9, 28040 Madrid, Spain; ∥ Ikerbasque, Basque Foundation for Science, Plaza Euskadi 5, 48009 Bilbao, Bizkaia, Spain; ⊥ Department of Organic Chemistry, II Faculty of Science and Technology, EHU-UPV, 48940 Leioa, Spain; Δ Centro de Investigación Biomédica En Red de Enfermedades Respiratorias, 28029 Madrid, Spain

## Abstract

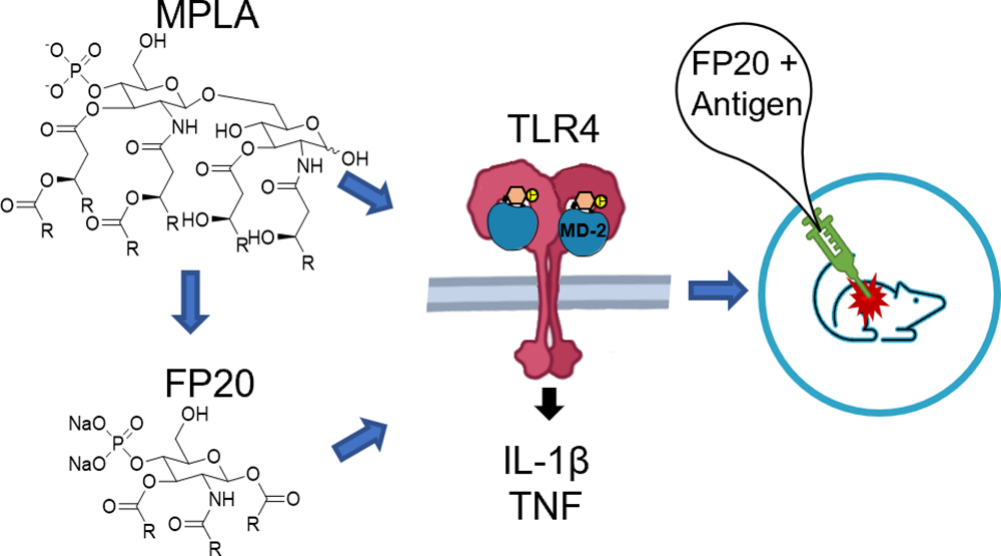

We disclose here a panel of small-molecule TLR4 agonists (the **FP20** series) whose structure is derived from previously developed TLR4 ligands (**FP18** series). The new molecules have increased chemical stability and a shorter, more efficient, and scalable synthesis. The **FP20** series showed selective activity as TLR4 agonists with a potency similar to **FP18**. Interestingly, despite the chemical similarity with the **FP18** series, **FP20** showed a different mechanism of action and immunofluorescence microscopy showed no NF-κB nor p-IRF-3 nuclear translocation but rather MAPK and NLRP3-dependent inflammasome activation. The computational studies related a 3D shape of **FP20** series with agonist binding properties inside the MD-2 pocket. **FP20** displayed a CMC value lower than 5 μM in water, and small unilamellar vesicle (SUV) formation was observed in the biological activity concentration range. **FP20** showed no toxicity in mouse vaccination experiments with OVA antigen and induced IgG production, thus indicating a promising adjuvant activity.

## Introduction

Vaccination is one of the most successful public health achievements ever carried out and continues to have a large impact in preventing the spread of infectious diseases worldwide.^1,2^ The most recent example of vaccines’ success was the Covid-19 pandemic and the impact of vaccination in the decrease of disease burden worldwide.^3^

Subunit vaccines contain specific purified pathogen antigens and show an improved safety profile compared with whole-pathogen vaccines, by eliminating the risk of incomplete inactivation.^4−6^ They are also often less immunogenic and require combination with adjuvants to enhance, accelerate, and prolong antigen-specific immune responses by triggering and modulating both the innate and adaptive immunity.^7,8^

Adjuvants also allow the decrease in antigen dose, reduce booster immunizations, generate more rapid and durable immune responses, and increase the effectiveness of vaccines in poor responders. Despite their key role, few sufficiently potent adjuvants with acceptable toxicity for human use are available in licensed vaccines. For more than 70 years, Alum (a mixture of diverse aluminum salts) has been the only approved adjuvant in humans.^9^ Besides aluminum salts, the other few molecules used in adjuvant systems (AS) approved for use in humans are the Toll-like receptor 4 (TLR4) agonist monophosphoryl lipid A (MPLA),^10^ squalene, and a simplified version of saponin, QS-21.^11^

MPLA is a detoxified analogue of lipid A, which is the membrane-anchoring part of gram-negative bacterial lipopolysaccharide (LPS).^12^ Lipid A is also the bioactive moiety of LPS, so most of molecules mimicking the LPS agonist activity on TLR4 are lipid A analogues.^13^ Specifically, MPLA is derived from *Salmonella minnesota* R595 lipid A and it is obtained through hydrolysis of the hydrophilic polysaccharide, of the C1 phosphate, and of the (*R*)-3-hydroxytetradecanoyl groups.^14^ The lack of the C1 phosphate group allows it to maintain its immunostimulating properties while eliminating toxicity. Its activity, as well as that of its synthetic analogue GLA, is based on TLR4 stimulation that results in promotion of Th1 (cellular)-biased immune response.^7,15,16^ MPLA is present in the formulation of AS used in vaccines: in combination with aluminum salts (Alum) in AS04, approved for the vaccine against human papillomavirus (HPV), Cervarix, and in AS01, a liposomal formulation containing MPLA and the natural product saponin QS-21, which has been recently approved for GSK’s malaria (Mosquirix) and shingles (Shingrix) vaccines.^17,18^

Due to the reduced chemical variety of approved adjuvants and the lack of clarification of their mechanism of action, there is still a pressing need for novel, potent, and less toxic adjuvants and new formulations for use in subunit vaccines. The accumulating knowledge on pattern recognition receptors (PPRs), as it is the case of TLR4, has led to the development of new adjuvants that target these receptors in immune cells.^19^

TLR4 activation initiates two intracellular signaling pathways: the MyD88-dependent pathway that is triggered upon formation of (TLR4/MD-2/LPS)_2_ activated dimers on the plasma membrane and leads to NF-kB nuclear translocation and production of pro-inflammatory cytokines such as TNF, IL-6, and pro-IL-1β, and the TRAM/TRIF-dependent pathway that starts after homodimer internalization and endosomal activation and leads to the production of type I interferon (IFN-β). Both pathways begin with the assembly of supramolecular clusters composed of different cytosolic proteins named, respectively, myddosome and triffosome.^20^

Recently, it has been observed that many clinical approved adjuvants, including alum and combinatorial vaccine adjuvants AS01 and AS04, promote immunogenicity also through inflammasome-mediated signaling, activating the NLRP3 inflammasome, which leads to the activation of caspase-1, resulting in cleavage of pro-IL-1β and pro-IL-18 and secretion of their mature forms.^21^ Importantly, the IL-1 family cytokines are important for T-cell activation and memory cell formation, which is crucial for the achievement of immune protection.^22^

**Figure 1:**
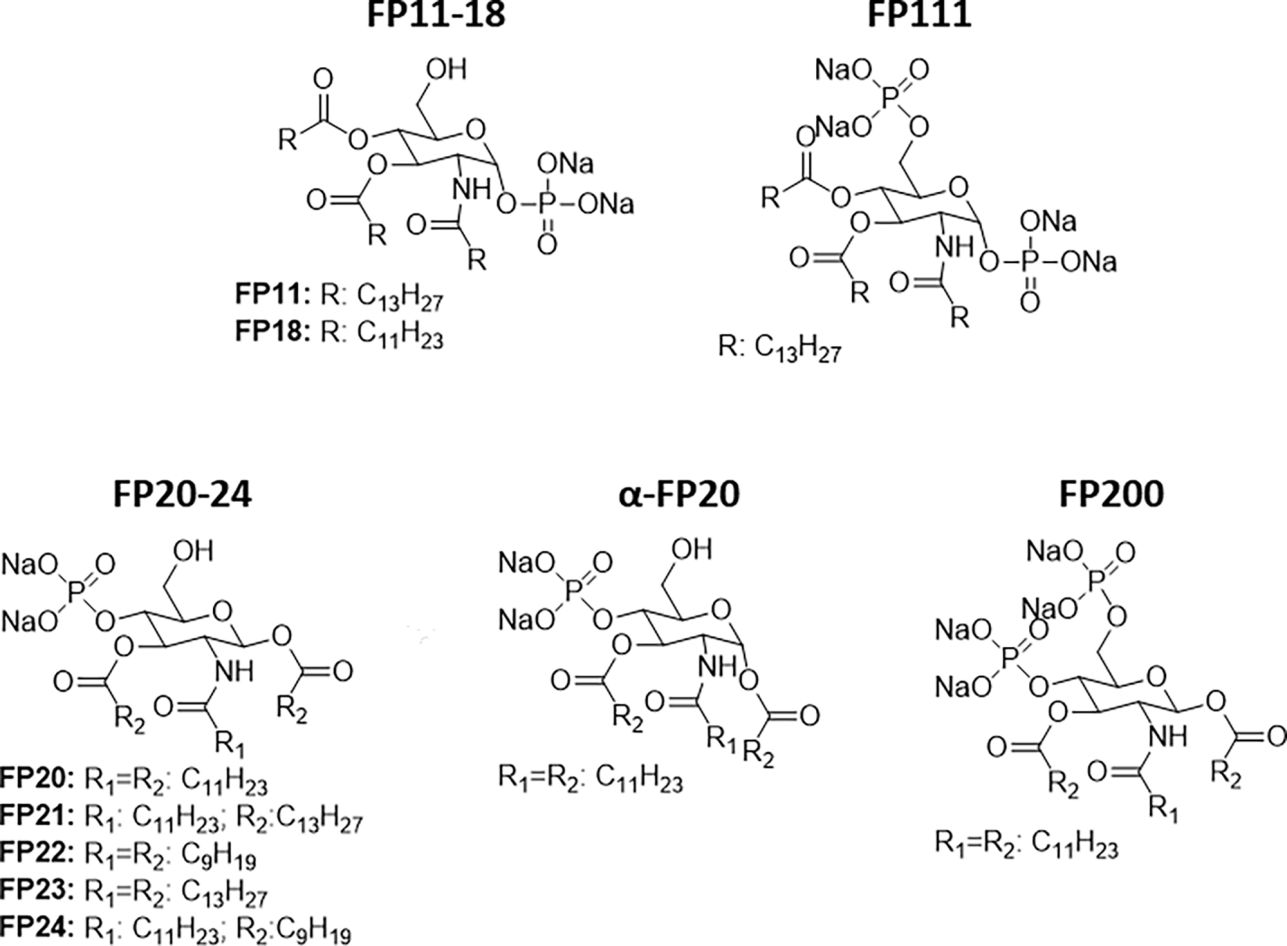
Structures of TLR4 agonists **FP11** and **18** and new molecules **FP20**–**24**, α-**FP20**, and **FP200**.

We recently reported the activity as vaccine adjuvants of two structurally simplified MPLA analogues, the **FP11** and **FP18** molecules (Figure 1), whose chemical structure is composed of the glucosamine monosaccharide bearing three fatty acid chains and one phosphate group in C1.^23^

Despite their simplified monosaccharide structures when compared to disaccharide lipid A and MPLA, the FP molecules, in particular **FP18**, strongly activate both MyD88- and TRAM/TRIF-dependent pathways, leading respectively to production of TNF, IL-6, and IFN-β. **FP18** also activates the NLRP3 inflammasome, thus inducing IL-1β maturation and release. Moreover, OVA immunization experiments showed that **FP18** has an adjuvant activity similar to MPL.^23^

The presence of the anomeric phosphate, which is a good leaving group, causes chemical instability of **FP18**-type compounds. We then designed a new series of triacylated glucosamine derivatives in which the C1 phosphate is moved to the C4 position.

Compounds **FP20**–**24** present variable chain lengths, with the anomeric acyl chain always in the beta-configuration. To better assess the structure–activity relationship, we also designed and synthesized a compound with anomeric acyl chain in the alfa configuration (α-**FP20**) and a molecule with both C4 and C6 positions phosphorylated (**FP200**).

## Results and Discussion

### Chemical Synthesis

**Scheme 1:**
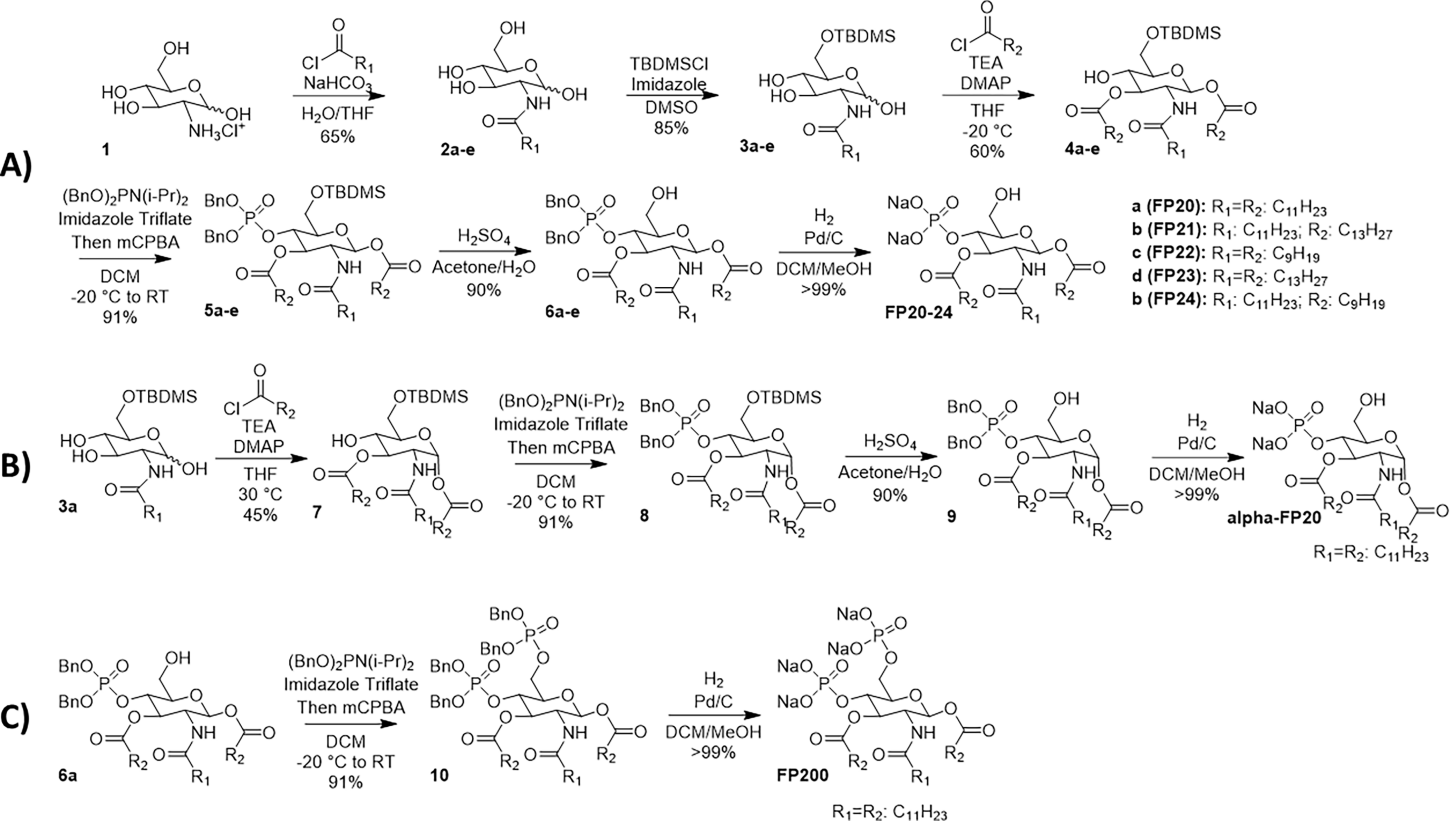
(A) Synthetic Pathway to Compounds **FP20**–**24**, (B) to Compound α-**FP20**, which Can Be Obtained Starting from Intermediate **3a**, and (C) to Compound **FP200**, which Can Be Obtained Starting from Intermediate **6a**. A Larger Version of This Scheme Is Provided in the SI

Compounds **FP20**–**24** were obtained by means of a six-step synthesis (Scheme 1A). Commercially available glucosamine hydrochloride was acylated on the amino group in position C2 with different acyl chlorides, obtaining compounds **2a**–**e**, which were regioselectively protected in the C6 position as *tert*-butyldimethylsilyl (TBDMS) ethers, obtaining compounds **3a**–**e**. The acylation of compounds **3a**–**e** by reaction with acyl chlorides in the presence of triethylamine (TEA) and *N*,*N*-dimethylaminopyridine (DMAP) in THF, at low temperature, afforded compounds **4a**–**e** with anomeric lipid chains in the beta configuration. The regio- and stereoselectivity observed is due to the combination of steric effects (TBDMS in C6 hindering position C4), electronic effects (increased nucleophilicity of beta-anomer), and solvent effects (the dipolar moment of the solvent THF partially suppresses the alpha effect). Compounds **4a**–**e** are then phosphorylated on the C4 position through the phosphite insertion strategy using dibenzyl *N*,*N*-diisopropylphosphoramidite, giving compounds **5a**–**e** that were desilylated in diluted sulfuric acid and then debenzylated by catalytic hydrogenation, thus obtaining the final compounds **FP20**–**24**. The overall yield was about 30% for all compounds.

This synthetic pathway is shorter than the previously published one for **FP11** and **FP18** compounds,^23^ as well as more cost effective because it only requires three purification steps instead of the seven required for **FP18**. Furthermore, **FP20** synthesis also requires three less steps than **FP18** and avoids the use of some toxic solvents (e.g., pyridine, DMF) used in the previous synthesis. By means of the same strategy and changing the conditions (temperature, concentration, and amount of catalyst) of acylation of compound **3a**, α-**FP20** was obtained (Scheme 1B). Compound **FP200** was obtained by phosphorylating compound **6a**, and then deprotecting compound **10** by catalytic hydrogenation, yielding product 14 with a 26% overall yield (Scheme 1C).

### Cryogenic Electron Microscopy (Cryo-EM) and Dynamic Light Scattering (DLS)

**Figure 2:**
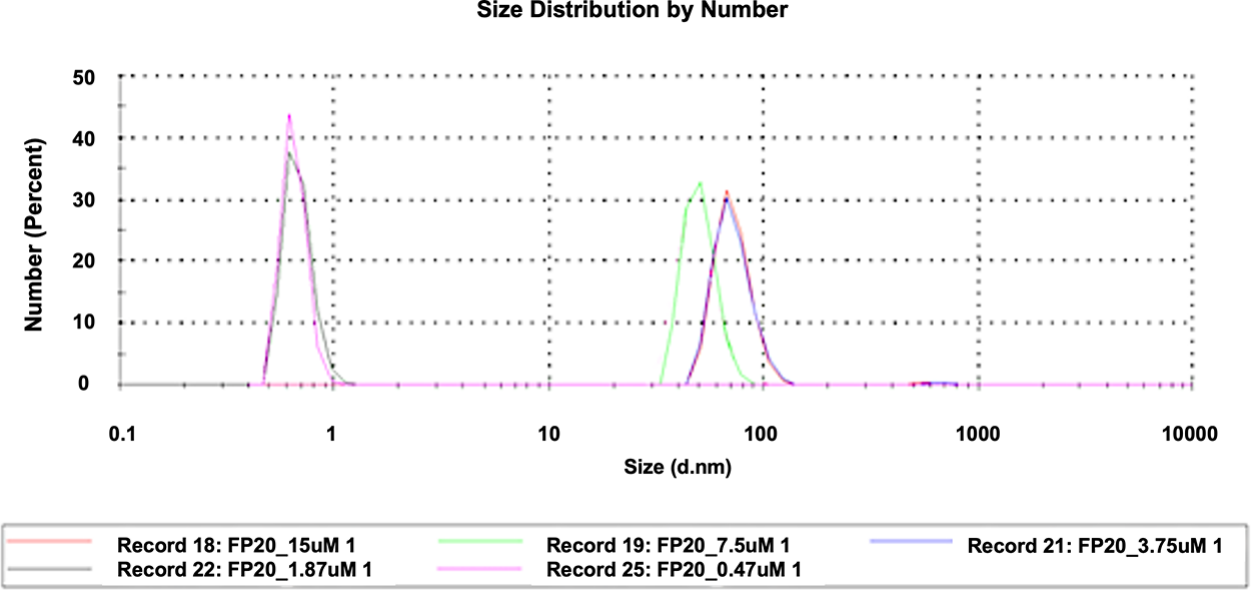
Detection of the hydrodynamic diameter of **FP20** at different concentrations in solution using DLS.

The aggregation behavior in solution of lipid A, lipid X, and their synthetic analogues strongly influences the potency of TLR4 agonists, so that it has been stated that aggregates are the biologically active units of endotoxin.^24^ It is therefore important to know the aggregation properties in the aqueous environment of this new family of compounds. In previous studies carried out with monosaccharide glycolipids derived from lipid X, such as in the case of **FP7** glycolipid,^25^ a critical micelle concentration (CMC) of 9 μM was found. Compound **FP15**, bearing two succinate esters instead of phosphates in C1 and C4 positions, formed spherical and homogeneous small unilamellar vesicles (SUVs).^26^ The different disposition of fatty acids and phosphate groups in the **FP20**-series compared to **FP11** affected the aggregation properties. A CMC value lower than 5 μM was found for **FP20** in water, since the formation of large aggregates can be observed even at such low concentrations. Simultaneously, DLS data indicated a CMC value between 1.87 and 3.75 μM, much lower than any other synthetic glycolipid and even lower than that of the parent lipid X (Figure 2). Furthermore, DLS allowed the calculation of the hydrodynamic diameter of **FP20** particles. Particles with a diameter of about 100 nm can be identified in solution when a concentration between 15 and 3.75 μM was used.

**Figure 3:**
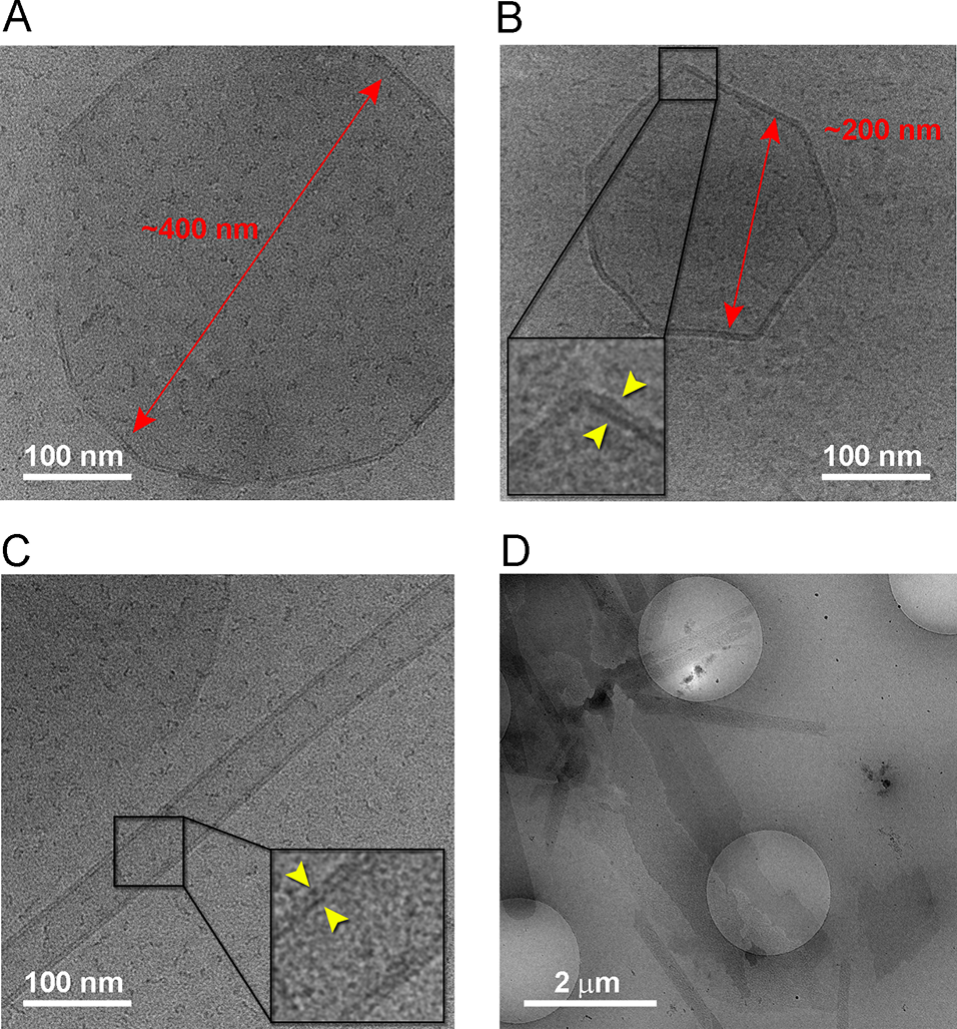
Cryo-EM images of **FP20** supramolecular structures formed at a concentration of 0.8 mM. (A) LUV; (B) polygonal LUV; (C) cylindrical vesicle and LUV; (D) low-magnification view of large assemblies present on the TEM grid. Insets have been enlarged to 2.5× from original and Gaussian-filtered for better clarity; yellow arrowheads mark the lipid bilayer whose thickness is about 0.4 nm.

**FP20** was selected as a model compound for transmission cryo-electron microscopy studies (Figure 3). Collected 2D images using glycolipid concentrations of 0.8 and 1.0 mM, respectively, showed few supramolecular structures. Some of these structures organized as large unilamellar vesicles (LUV) with a diameter ranging from about 130 to 400 nm, some with a polygonal shape (Figure 3A,B). Others were assembling in cylindrical vesicles with different lengths and in bilayer sheets (Figure 3B,C). When lower concentrations of the glycolipid **FP20** were used in DLS experiments, no large aggregates can be detected. The combined cryo-EM and DLS results might indicate that at lower concentrations **FP20** leans toward the formation SUVs, but when the concentration is increased then higher-order aggregates, such as LUV and/or cylindrical vesicles, start forming simultaneously.

### TLR4 Selectivity Studies in HEK-Blue hTLR4 and hTLR2

**Figure 4:**
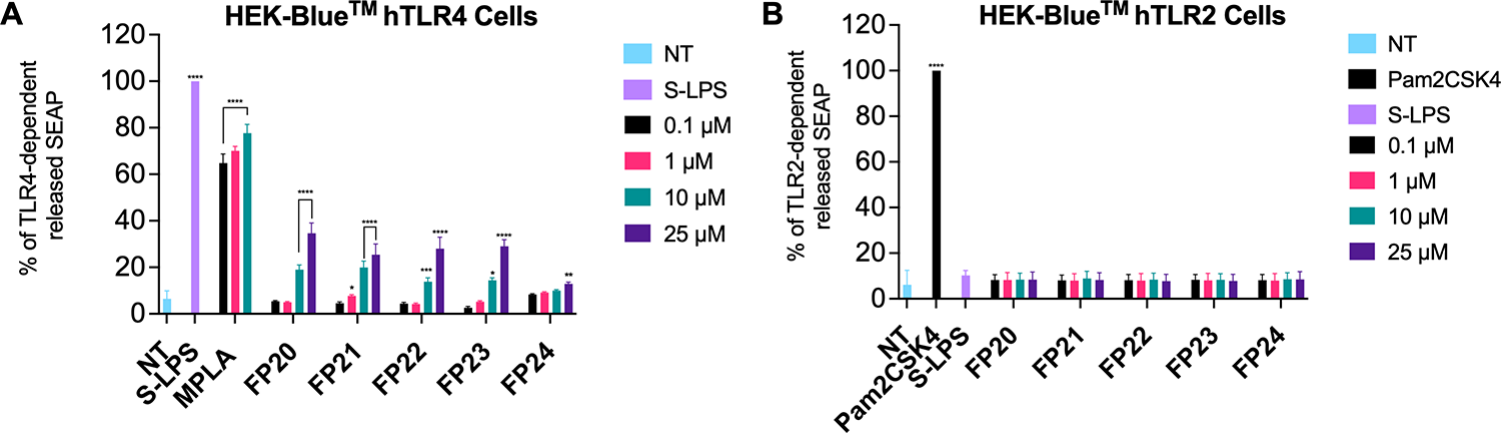
Selectivity of FP compounds toward TLR4. HEK-Blue hTLR4 cells (A) and HEK-Blue TLR2 (B) were treated with the shown concentrations of **FP20**–**24**, MPLA, LPS (100 ng/mL), and Pam_2_CSK_4_ (1 ng/mL) and incubated for 16–18 h. The 100% stimulation has been assigned to the positive control LPS (A) or Pam_2_CSK_4_ (B). Data are expressed as mean ± SEM of at least three independent experiments (treated vs non-treated: *P < 0.05; **P < 0.01; ***P < 0.001; ****P < 0.0001).

The selectivity of compounds **FP20**–**24** toward human TLR4 was investigated using specific HEK reporter cell lines. HEK-Blue hTLR4 and HEK-Blue hTLR2 (InvivoGen) are cell lines designed to study the activation of human TLR4 and TLR2 receptors, respectively, by monitoring the activation of transcription factors NF-κB and AP-1. Stimulation with TLR4 (HEK-Blue hTLR4) or TLR2 ligands (HEK-Blue hTLR2) activates NF-κB and AP-1, inducing the production and release of the secreted embryonic alkaline phosphatase (SEAP) in the extracellular environment. SEAP release can then be measured using a colorimetric assay, QUANTI-Blue (InvivoGen), which relies on the ability of SEAP to process its substrate generating a chromogenic product whose wavelength of maximum absorbance is 630 nm. HEK-Blue hTLR4 (Figure 4A) and HEK-Blue hTLR2 (Figure 4B) were treated with increasing concentrations of **FP20**–**24** (0.1–25 μM) and incubated for 18 h. Smooth LPS (S-LPS) from *S. minnesota* and MPLA were used as positive controls for TLR4 activation, while Pam_2_CSK_4_ was used as a positive control for the TLR2-mediated response. As shown in Figure 4, all compounds showed selective activity toward TLR4 and were inactive on TLR2.

### Activity on THP-1 Derived Macrophages (TDM)

To assess the biological activity of new compounds, an initial screening was performed using the THP-1 X-Blue cell line. Human THP-1 X-Blue monocytes were differentiated into macrophages by exposure to PMA (100 ng/mL). THP1 X-Blue (InvivoGen) were derived from the human THP-1 monocyte cell line by stable integration of an NF-κB/AP-1-inducible SEAP reporter construct. The analysis of levels of NF-κB/AP-1-induced SEAP in the cell culture supernatant, which correlates with the activation of the NF-κB/AP-1 pathway, was performed using QUANTI-Blue solution, a SEAP detection reagent (InvivoGen).

**Figure 5:**
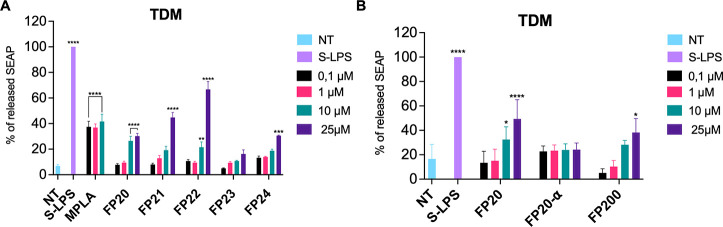
Activity of FP compounds in TDM. Differentiated THP1 X-Blue cells were treated with the shown concentrations of **FP20**, **FP21**, **FP22**, **FP23**, and **FP24** (A) or **FP20**, α-**FP20**, and **FP200** (B) and incubated for 16–18 h. MPLA and S-LPS (100 ng/mL) were used as controls. The 100% stimulation has been assigned to the positive control. Data are expressed as mean ± SEM of at least three independent experiments (treated vs non-treated: *P < 0.05; **P < 0.01; ***P < 0.001; ****P < 0.0001).

Cells were treated with increasing concentrations of **FP20**–**24** (0.1–25 μM) and incubated for 18 h. Smooth LPS (S-LPS) from *S. minnesota* and MPLA were used as positive controls. Results show that most compounds significantly induce SEAP release in the human myeloid cell line in a dose-dependent manner (Figure 5A). Compound **FP23**, with C_14_ fatty acid (FA) chains, is the only glycolipid whose activity is not statistically significant in macrophage-like human cells. On the contrary, **FP22**, with C_10_ FA, shows an increased NF-κB/AP-1 activation when compared to **FP20**.

To further investigate the SAR of this series of compounds in human cells, α-**FP20** and **FP200** were tested in TDM using the same assay as the previously mentioned derivatives. As shown in Figure 5B, α-**FP20** with the alpha-anomeric FA chain shows no significant activity. This fact points out the importance of the anomeric configuration of the lipid chain in C1, which can affect the physico-chemical properties of FP aggregates in the extracellular aqueous medium and have a direct impact in their detection by innate immune system.^27,28^ The LPS-binding protein (LPB) transfers FP monomers to TLR4/MD2 via the interaction with CD14. It is indeed probable that if the anomeric carbon is in the beta configuration, the lipid chain lies further away from the chains in 2-OH and 3-OH, decreasing the hydrophobic interaction between the **β**-**FP20** lipid chains and weakening the packing of glycolipids,^29^ which can facilitate their transfer to TLR4 through the LBP.^30^

We performed molecular dynamics (MD) simulations to reveal the influence of the anomeric configuration on the packing of α-**FP20** and **FP20** (with β-anomeric configuration) glycolipids at the atomic level (thorough explanation and discussion are given in the Supporting Information). Starting from a random mixture of either α-**FP20** or **FP20** molecules in water (see Computational Methods for details), the two systems were observed to self-organize into a bilayer (Figure S1). The calculated bilayer area was greater for **FP20** compared to α-**FP20** (Figure S2), indicating that the lipid chains are less packed in the case of the β-anomer **FP20**. In each monolayer, **FP20** molecules were regrouped in assemblies (see the Supporting Information). Interestingly, in the α-**FP20** molecules, the carbonyl group of the acyl chain at the anomeric carbon was always pointing in the same direction in all the molecules that formed the same arrangement (see Figure S3). Thus, this position of the carbonyl group can contribute to the ordering of the bilayer, driven by entropic factors, suggesting that the α-**FP20** compound induces a more ordered phase in the **FP20** assemblies, which favors more compact lipid packing, and can make the transfer of α-**FP20** along the TLR4 extracellular cascade difficult, in agreement with the experimental lack of activity observed for this compound.

**FP200**, a derivative with two phosphates, was also tested. We previously reported that compound **FP111** (Figure 1), the di-phosphorylated analogue of **FP11**, was inactive as TLR4 agonist.^23^ In contrast with this observation,^23^**FP200** retains activity as TLR4 agonist (Figure 5B). This indicates that the position of the phosphate groups on the glucosamine scaffold is important for activity.

### Computational Studies of the TLR4 Binding of **FP20**, **FP22**, and **FP24**

Compounds **FP20**, **FP22**, and **FP24** were selected as representative compounds to study computationally and to provide insights at the atomic level of their binding to TLR4. The 3D structure of the human (TLR4/MD-2)_2_ heterodimer in the agonist conformation was first used (PDB ID 3FXI)^31^ to carry out molecular docking calculations, followed by MD calculations of selected (TLR4/MD-2/ligand)_2_ complexes.

**Figure 6:**
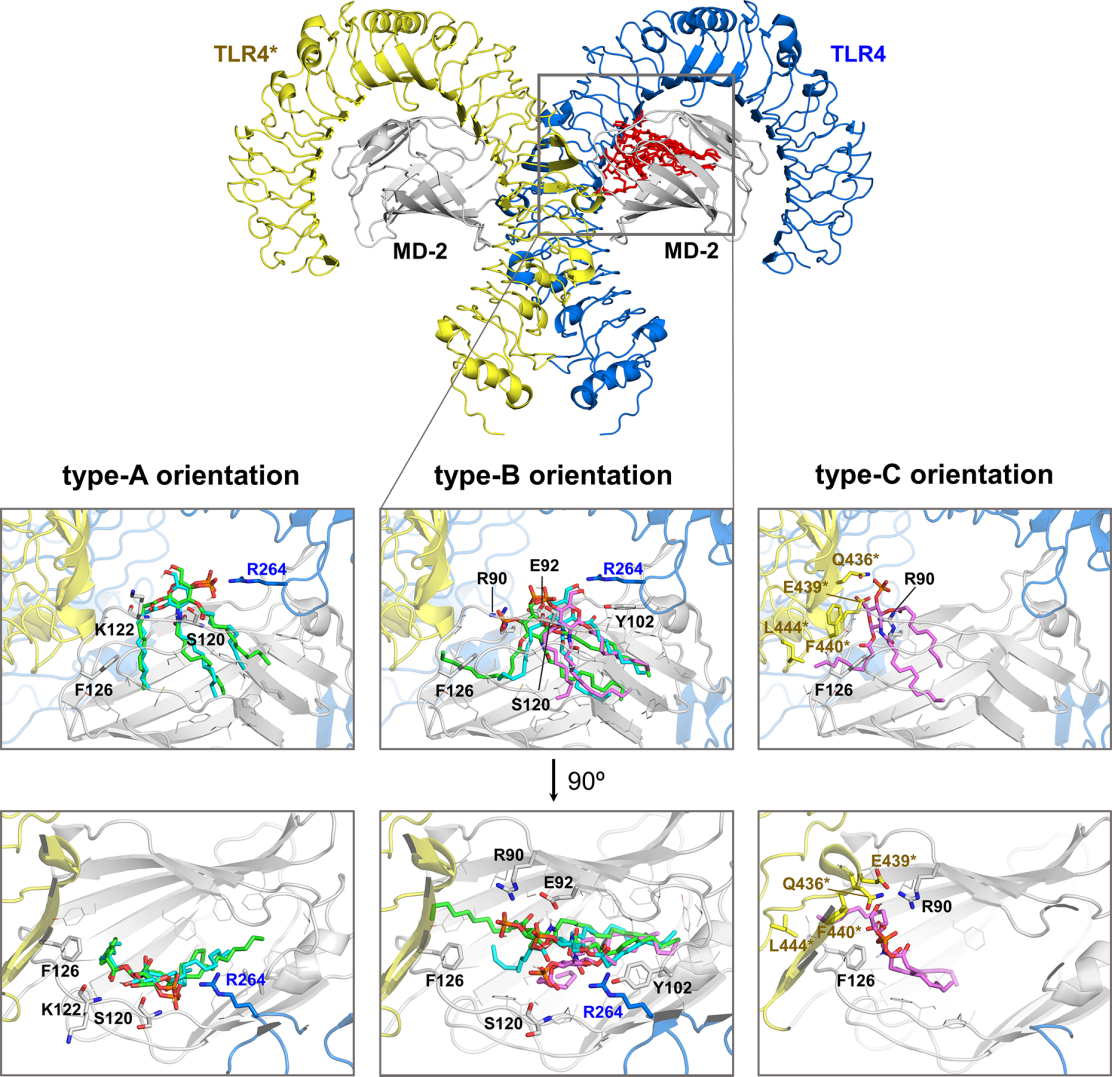
Compounds **FP20**, **FP22**, and **FP24** docked into the (TLR4/MD-2)_2_ complex (PDB ID 3FXI). Top: 3D structure of a human (TLR4/MD-2)_2_ dimer (yellow, dark blue, and gray cartoon) used for docking calculations to assess the binding of **FP20**, **FP22**, and **FP24** (all in red sticks). Below: docked poses corresponding to type-A, -B, and -C orientations; the best AutoDock 4-predicted binding modes for ligands **FP20** (green), **FP22** (blue), and **FP24** (magenta) are shown in sticks. For each binding mode, the front view (top) and the 90° rotated view (below) are depicted, as well as details of the interactions with residues of TLR4 (blue sticks), MD-2 (gray sticks), and TLR4* (yellow sticks). Type-A and -B poses differ by 180° rotation along the lipid chains axis: binding orientation type A places the phosphate group pointing toward residue Arg264 of TLR4, whereas binding orientation type B orients the phosphate pointing toward the partner TLR4. PDB files are available online at (include link).

Preliminary docking calculations performed with AutoDock Vina^32^ predicted plausible binding modes for all the explored ligands. Most docked poses can be classified into three main binding types (types A, B, and C) (Figure 6). The binding poses inserted the FA chains into the hydrophobic pocket of MD-2 interacting with many hydrophobic and aromatic residues, and with the saccharide moiety positioned at the MD-2 rim, either establishing polar interactions with residues from the MD-2 entrance (type-A and -B binding modes, rotated 180° between them, see the Supporting Information) or shifted upward toward the partner TLR4 (designated TLR4*) allowing the formation of polar interactions with TLR4* residues at the dimerization interface (type-C binding mode) (Figure 6 and Figure S4).

These predicted poses were used as starting geometries for redocking calculations with AutoDock 4.^33^ Similarly to Vina, AutoDock 4 also predicted poses belonging to the type-A, -B, and -C binding modes. Nevertheless, not all the binding types (A, B, and C) were predicted for the three FP ligands in the redocking calculations; type A was predicted for **FP20** and **FP22**, type B for all the ligands, and type C only for **FP24** (see Table S1).

Regarding the positioning of the FA chains, two different behaviors were observed for all the ligands. Independently of the binding mode (type A, B, or C), in most cases, the three FA chains were placed inside the MD-2 pocket. However, some poses placed one FA chain (either C1 or C3 chain) into the MD-2 channel delimited by Phe126 (Figure S4)^31^ and the other FA chains inside the MD-2 cavity (Table S1). Overall, the docking predictions point to a different behavior for **FP24** in comparison to **FP20** and **FP22** (see the Supporting Information).

The stability of the best-predicted binding modes was confirmed by MD simulations (200 ns) (Figures S5 and S6, and complete description in the Supporting Information). The root mean square deviation (RMSD) was monitored along the simulation time and confirmed the stability of the (TLR4/MD-2/ligand)_2_ complexes (Figures S7 and S8). The orientation of the FP molecules along the simulation was assessed, observing that the FP compounds did not undergo orientation flip, pointing to the ability of these ligands to interact with TLR4 in different orientations (Figure S9). Remarkably, the **FP24** type-C binding mode turned into type-A. During MD simulations, most interactions were maintained for **FP20** and **FP22** binding poses and, additionally, new interactions with the key TLR4 residues Lys341 and Lys362^31^ were formed. Conversely, for **FP24** complexes, the important interactions with either TLR4 Lys341 or Lys362 were not observed at the end of the simulations (Figures S10 and S11). Regarding the FA chains, the acyl chain initially placed at the MD-2 channel migrated into the MD-2 pocket in all cases (Figures S12 and S13). Despite this common observation, a different behavior was detected for **FP24** acyl chains compared with **FP20** and **FP22**: whereas **FP20** and **FP22** FA chains were inserted linearly into the MD-2 pocket, the **FP24** FA chains were often bent, especially FA chain C2, the longest one. Interestingly, **FP20** and **FP22** retained the Phe126 agonist conformation in both MD-2 chains of the (TLR4/MD-2/ligand)_2_ complex only in the type-B binding mode, but **FP24** only in type-A (type-C at the beginning of the simulations) (Figure S14).

As observed from the docking calculations and the MD simulations, **FP24** behaves differently than **FP20** and **FP22**, in agreement with the fact that **FP24** is less active in stimulating TLR4. Although **FP24** was reaching similar regions of the MD-2 pocket as **FP20**, the sugar moiety was not able to establish interactions that are key in TLR4 agonist recognition. Since the shape of the LPS lipid A component may be a key determinant for the TLR4 activation,^34^ we wondered about the shape of these three FP analogues, finding that the active compounds **FP20** and **FP22** adopted a cylindrical shape, whereas the less-active **FP24** displayed a different shape (see the Supporting Information and Figure S15) impeding potential polar interactions that occur for **FP20** and **FP22** saccharide moieties. Altogether, our computational studies suggest that there is an optimal shape and length for the FA chains for an appropriate TLR4 agonist binding, in addition to the presence of a single phosphate group and its positioning at the pyranose ring.

### Pro-Inflammatory Cytokine Profile in Human Macrophages

Lead compounds **FP20**, **21**, and **22** were tested on both TDM and primary human macrophages in order to evaluate their adjuvant activity. First is the release of pro-inflammatory cytokines after treatment with the synthetic glycolipids, in the same human macrophage-like model mentioned above. S-LPS from *S. minnesota* served as positive control as it induces the release of TNF, IL-β, and IL-6 upon binding to TLR4 and activation of the MyD88 pathway and the inflammasome.

**Figure 7:**
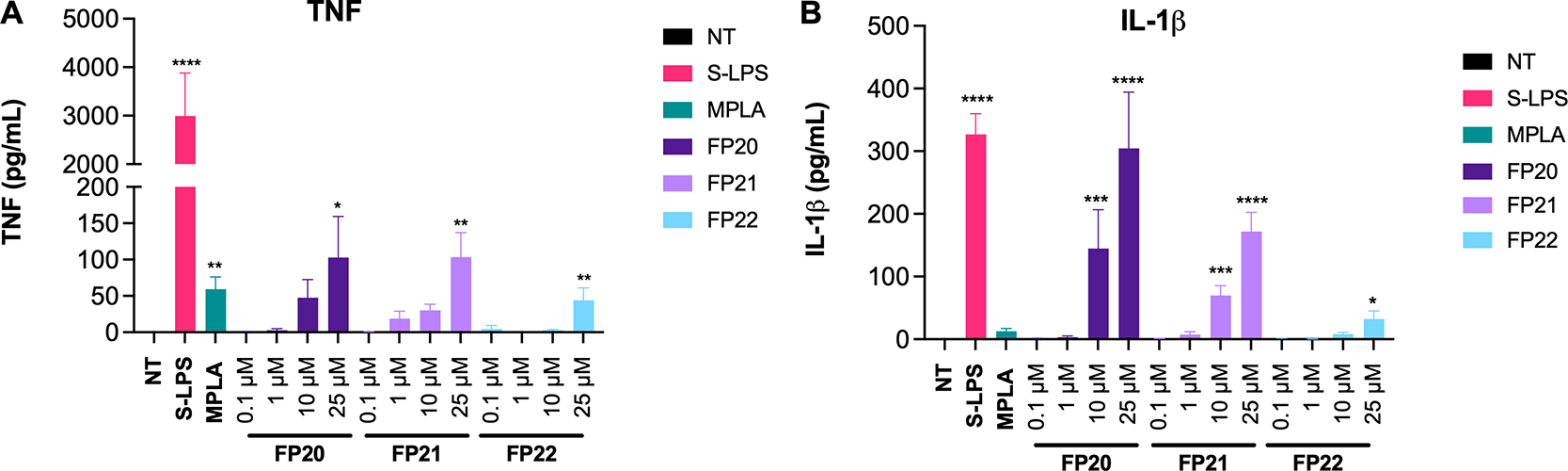
FP compound pro-inflammatory cytokine release in TDM. Differentiated THP1-XBlue cells were treated with increasing concentrations of **FP20**, **21**, and **22** (0.1–25 μM) and LPS (100 ng/mL). TNF (A) and IL-1β (B) levels were evaluated by ELISA after 6 h of incubation. Data are expressed as mean ± SEM of at least three independent experiments (treated vs non-treated: *P < 0.05; **P < 0.01; ***P < 0.001; ****P < 0.0001).

**FP20**, **21**, and **22** induced a significant TNF release in TDM only when used at the highest concentration (25 μM) and in reduced amounts compared to S-LPS (Figure 7A). In contrast, a remarkable, dose-dependent release of IL-1β was observed upon **FP20** and **FP21** treatment. **FP20** was able to induce a level of IL-1β comparable to S-LPS at the highest tested concentration (25 μM) (Figure 7B). The three tested glycolipids were not able to induce IL-6 secretion in TDM. MTT assays were performed to assess cytotoxicity in TDM, and the results revealed that the compounds did not affect the cell viability (see the Supplementary Information).

**Figure 8:**
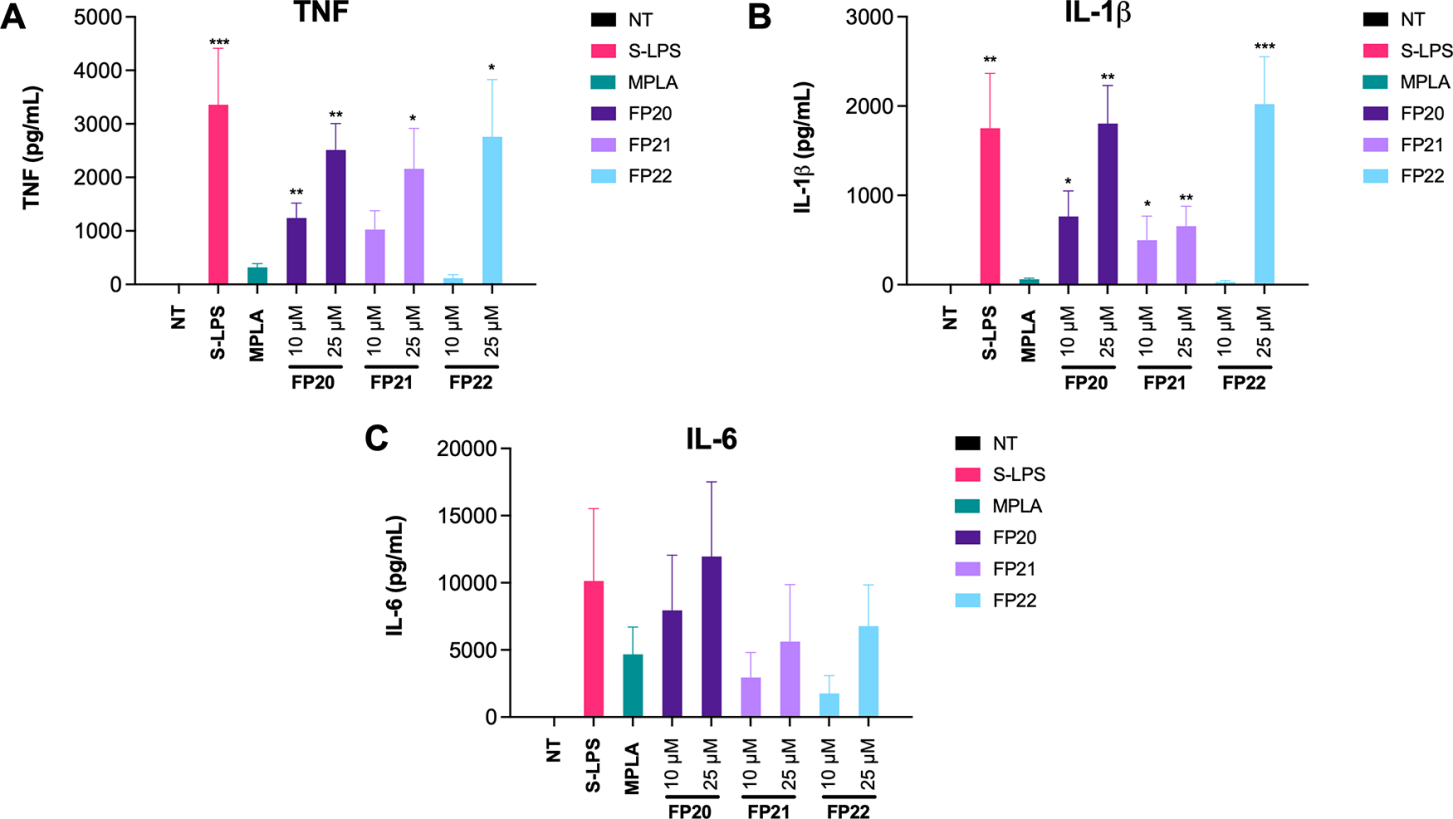
Cytokine release induction by FP molecules in PBMCs. PBMCs were treated with two selected active concentrations of **FP20**, **21**, and **22** (10 and 25 μM) and LPS (100 ng/mL). TNF (A), IL-1β (B), and IL-6 (C) levels were evaluated by ELISA after 6 h of incubation. Data are expressed as mean ± SEM of at least three independent experiments (treated vs non-treated: *P < 0.05; **P < 0.01; ***P < 0.001; ****P < 0.0001).

To evaluate pro-inflammatory cytokine production in primary cells, peripheral blood mononuclear cells (PBMCs) were isolated from whole blood of healthy donors and treated with 10 and 25 μM of **FP20**–**22**. After 6 h of incubation, cytokine release was measured via ELISA. Results show the ability of these molecules to induce a dose-dependent TNF and IL-1β production (Figure 8A,B). The release of IL-6 from stimulated PBMCs was highly variable among donors even in the case of S-LPS and MPLA stimulation (see error bars); consequently, it was not possible to appreciate a statistically significant correlation between the different treatments and the production of this cytokine (Figure 8C). In addition, since we do not observe any IL-6 production in TDM, we can assume that the IL-6 release observed in PBMCs might result from the contribution of mononuclear cells different from macrophages (e.g., monocytes and lymphocytes), which can contribute to the observed variability. MTT assays were performed to assess cytotoxicity in PBMCs. None of the compounds affected cell viability even at higher concentrations (0.1–25 μM) (see the Supporting Information).

### **FP20** Mechanism of Action Studies in TDM

**Figure 9:**
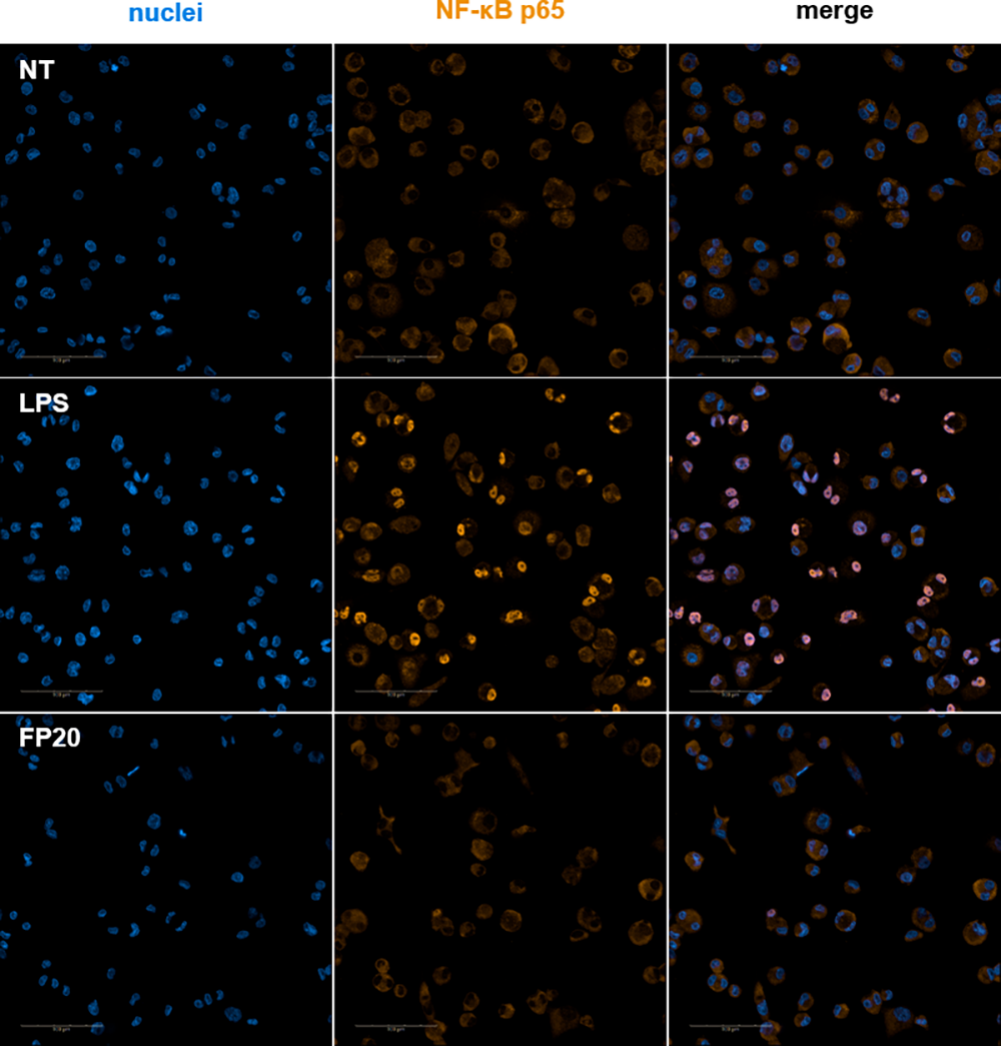
Immunofluorescence analysis of NF-κB translocation. Phospho-NF-κB localization in THP-1-derived macrophages (TDM) after LPS stimulation and **FP20** 25 μM treatment at *t* = 1.5 h.

The mechanism of action of **FP20** was investigated in TDM, a simple and reliable cell line model to study macrophage activity in inflammation.^35^ Since we observed a modest but significative release of TNF when compared with S-LPS, we wanted to elucidate the effect of **FP20** on the MyD88-dependent NF-κB pathway. We did not detect any p-p65 by immunodetection (Western blot assay—data not shown). To confirm this data, we employed immunofluorescence analysis using the Operetta CLS High Content Analysis System (PerkinElmer). The results showed no p65 translocation into the nucleus upon treatment with **FP20** between 0 and 4 h, while treatment with S-LPS triggered p65 translocation with a peak at 1.5 h (Figure 9).

**Figure 10:**
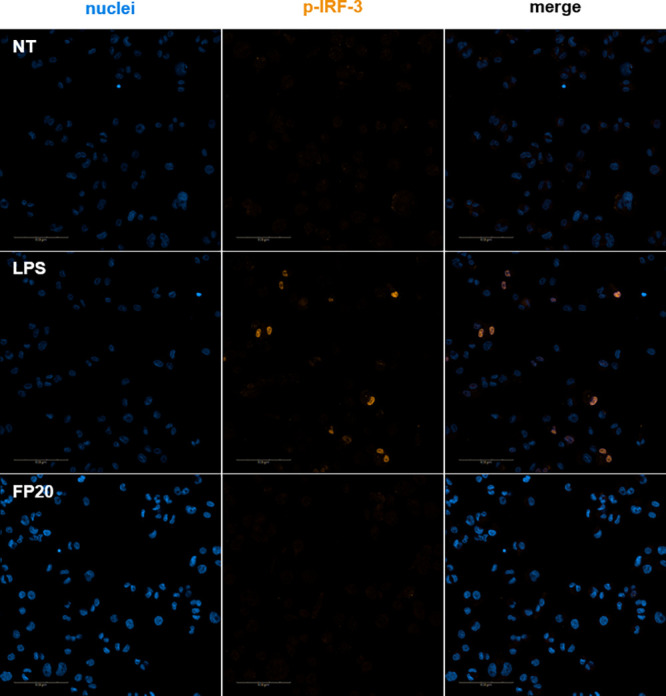
Immunofluorescence analysis of p-IRF-3 nuclear translocation at 2 h. Phospho-IRF-3 localization in THP-1-derived macrophages (TDM) after LPS stimulation and **FP20** 25 μM treatment at *t* = 2 h.

**Figure 11:**
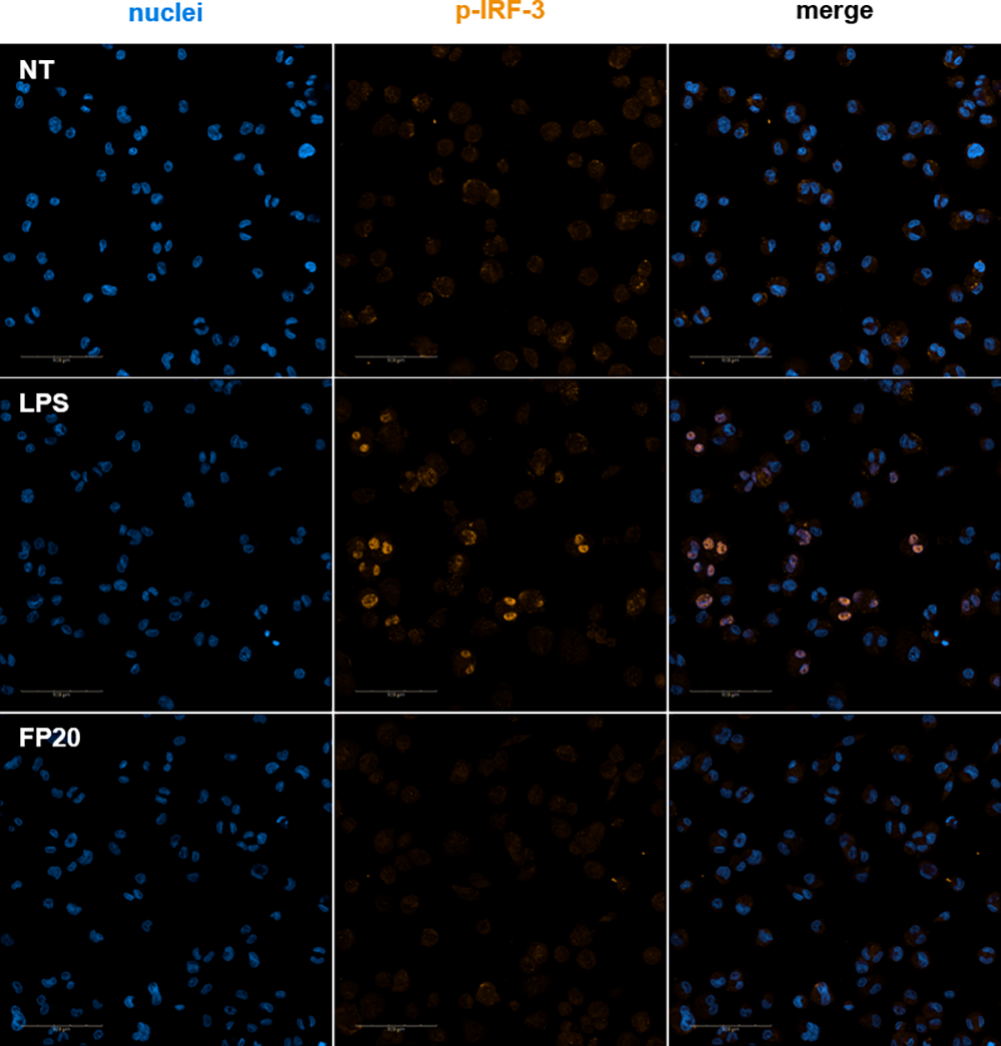
Immunofluorescence analysis of p-IRF-3 nuclear translocation at 4 h. Phospho-IRF-3 localization in THP-1-derived macrophages (TDM) after LPS stimulation and **FP20** 25 μM treatment at *t* = 4 h.

Following the same strategy, we studied the involvement of the TRIF/IRF3 axis in the FP-induced intracellular signaling. IRF-3 phosphorylation was assessed using Western blotting and also using the Operetta CLS High Content Analysis System (PerkinElmer). As in the case of the p-NF-κB p65 subunit, p-IRF-3 was not detected by immunoblotting (data not shown) and these data were confirmed by immunofluorescence analysis. As shown in Figure 10, the positive control, S-LPS, was able to induce p-IRF-3 nuclear translocation at 2 h, when p-IRF-3 translocation reached its peak (Figure 10), and 4 h (Figure 11), while **FP20** did not induce any phosphorylation and therefore no nuclear translocation as in the case of non-treated samples.

**Figure 12:**
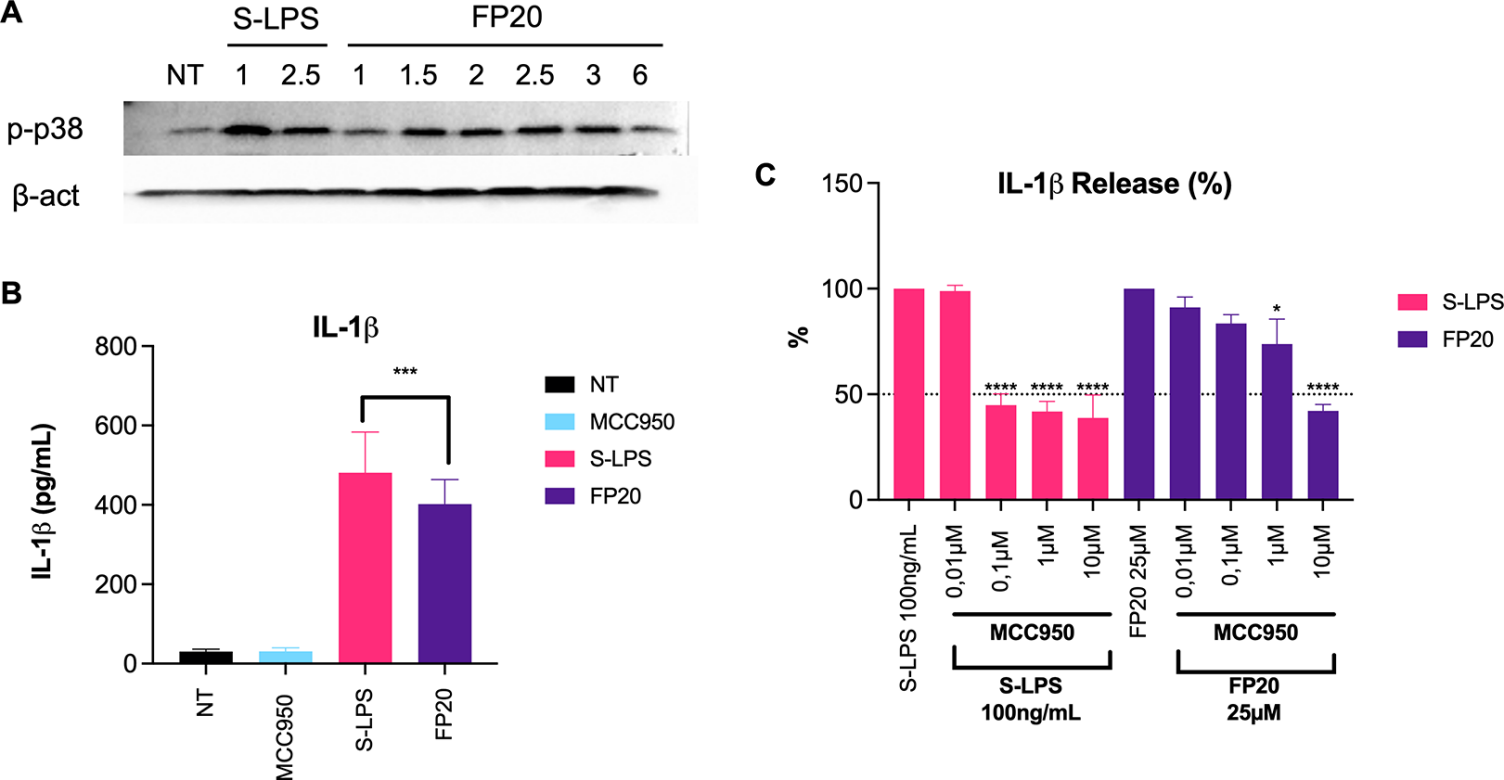
(A) Differentiated THP1-XBlue cells were treated with 25 μM of **FP20** or 100 ng/mL of S-LPS from 0 to 6 h. p-p38 MAPK was detected by Western blot in cell lysates. (B) Differentiated THP1-XBlue cells were treated with 25 μM of **FP20** or 100 ng/mL of S-LPS for 6 h. IL-1β levels in supernatant were detected by ELISA. Data are expressed as mean ± SEM of at least three independent experiments (treated vs non-treated: *P < 0.05; **P < 0.01; ***P < 0.001; ****P < 0.0001). (C) Differentiated THP1-XBlue cells were pre-treated with NLRP3 inhibitor MCC950 for 1 h followed by treatment with 25 μM of **FP20** or 100 ng/mL of S-LPS for 6 h. The effect of MCC950 on IL-1β release was measured in supernatants using ELISA and expressed as percentage. Data are expressed as mean ± SEM of at least three independent experiments (treated vs non-treated: *P < 0.05; **P < 0.01; ***P < 0.001; ****P < 0.0001).

Considering the lack of activation and nuclear translocation of the NFκB p-65 subunit and of IRF-3, we decided to investigate p38 mitogen-activated protein kinase (MAPK), since it is known to play an important role in TLR4-mediated inflammatory response after the activation of the receptor and the assembly of myddosome.^36^ It is reported that activation of MAPK cascades (p38 and JNK) leads to the phosphorylation of AP-1 components and consequently to their nuclei translocation.^20^ AP-1-induced transcription is associated with the production of proinflammatory cytokines,^37^ and p38 activation has been linked with the induction of TNF in cells treated with TLR4 agonist MPLA.^38^ Western blot analysis showed that **FP20** was able to induce a significant activation of p-p38 MAPK at 1.5, 2, and 2.5 h (Figure 12A). This can explain both the production of TNF in the absence of active p-p65 in **FP20**-treated TDM and the **FP20**-induced SEAP release, which can be ascribable to AP-1 activation.

**Figure 13:**
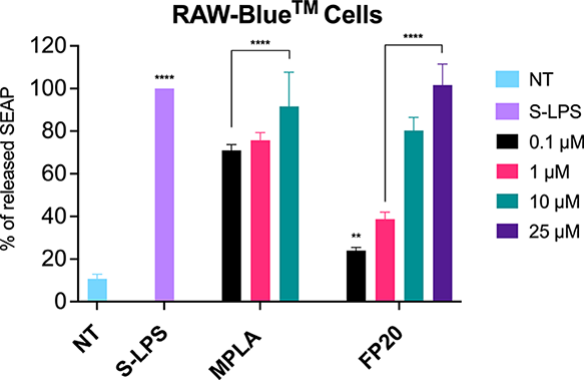
Activity of **FP20** in murine macrophages. RAW-Blue cells were treated with the shown concentrations of **FP20** and incubated for 16–18 h. MPLA and LPS (100 ng/mL) were used as controls. The 100% stimulation has been assigned to the positive control LPS. Data are expressed as mean ± SEM of at least three independent experiments (treated vs non-treated: *P < 0.05; **P < 0.01; ***P < 0.001; ****P < 0.0001).

Nevertheless, looking into the **FP20** cytokine profile, it is clear that the levels of IL-1β are comparable to S-LPS, which indicates that its release is significant for activity and may greatly contribute to explain **FP20** activity. It is known that p38 also plays a role in regulating pro-IL-1β transcription,^39^ while activation of the NLRP3 inflammasome and its downstream cascade is essential to the cleavage of pro-IL-1β and the release of mature IL-1β^40^ through membrane permeabilization.^41^ Considering the abovementioned results, we set out to investigate whether the NLRP3 inflammasome was involved in **FP20** activity. First, we measured the amount of IL-1β released at 6 h after S-LPS or **FP20** treatment (Figure 12B). Then, we pre-treated TDM with increasing concentrations of MCC950 (0.01–10 μM), a known NLRP3 inhibitor, prior to 6 h of S-LPS or **FP20** in order to observe its impact on IL-1β release. We observed a dose-dependent inhibition that in the case of **FP20** resulted in a decrease of IL-1β in the range of 50% with the highest dose of MCC950 (Figure 13C). This result confirms the involvement that NLRP3 inflammasome in the **FP20**-induced production and release of IL-1β. This mechanism of action is particularly interesting in the case of vaccine adjuvant development, since inflammasome-mediated immunogenicity is necessary to mount a proper immune response.^21^

### Activity in Murine Cells and in Vivo Immunization Experiments

Prior to in vivo immunization, **FP20** was tested using the RAW-Blue cell line (InvivoGen). This murine cell line is derived from the murine RAW 264.7 macrophage-like cells with chromosomal integration of a SEAP reporter construct inducible by NF-κB and AP-1. As shown in Figure 12, **FP20** displays a higher activity in the murine cell line, when compared to human cells. In fact, the compound is active at the lowest concentration tested (0.1 μM) in RAW-Blue cells while in TDM cells, the activity is only significant from a 100-fold higher concentration (10 μM). This species-specific activity has been observed in the case of similar TLR4 antagonists,^42^ and it is related to differences in the structure and binding sites in the human and murine TLR4/MD-2 receptor complex.^43−45^ Overall, these data confirm that **FP20** is active in a murine cell line and thus it is worth it to test its efficacy and safety in vivo.

**Figure 14:**
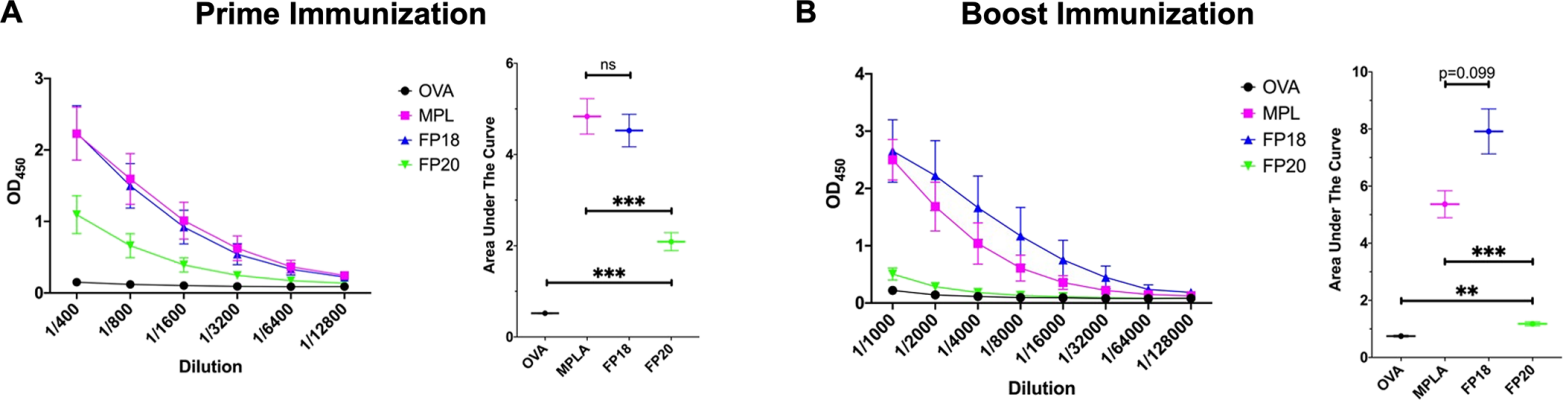
C57BL/6 mice were immunized with OVA formulated with or without MPLA, **FP18**, and **FP20** as adjuvants. (A) Total antibody response to prime OVA immunization 21 days post immunization. (B) Total antibody response to boost immunization 42 days post immunization. Values represent mean ± SEM. Brown–Forsythe and Welch one-way ANOVA tests (with an alpha of 0.05) were utilized to compare the areas under each curve. *p < 0.05; **p < 0.01; ***p < 0.001.

In order to evaluate the adjuvant efficacy of **FP20** in vivo, C57BL/6 mice were immunized with 10 μg of the model antigen chicken ovalbumin (OVA), formulated with or without 10 μg of MPLA, the previously developed agonist **FP18**, and **FP20**. MPLA and **FP18** were used as controls. After 21 days, the total anti-OVA IgG response was evaluated. Following the priming immunization, **FP18** was used as well as the MPLA control, and **FP20** produced significantly higher titers than the OVA control (Figure 14A). Mice then received a boost immunization on day 22, and final antibody responses, including IgG subtyping, were evaluated on day 42. Although not statistically significant, **FP18** generated slightly higher total IgG titers than MPLA, while **FP20** titers were significantly higher than OVA but lower than MPLA (Figure 14B).

**Figure 15:**
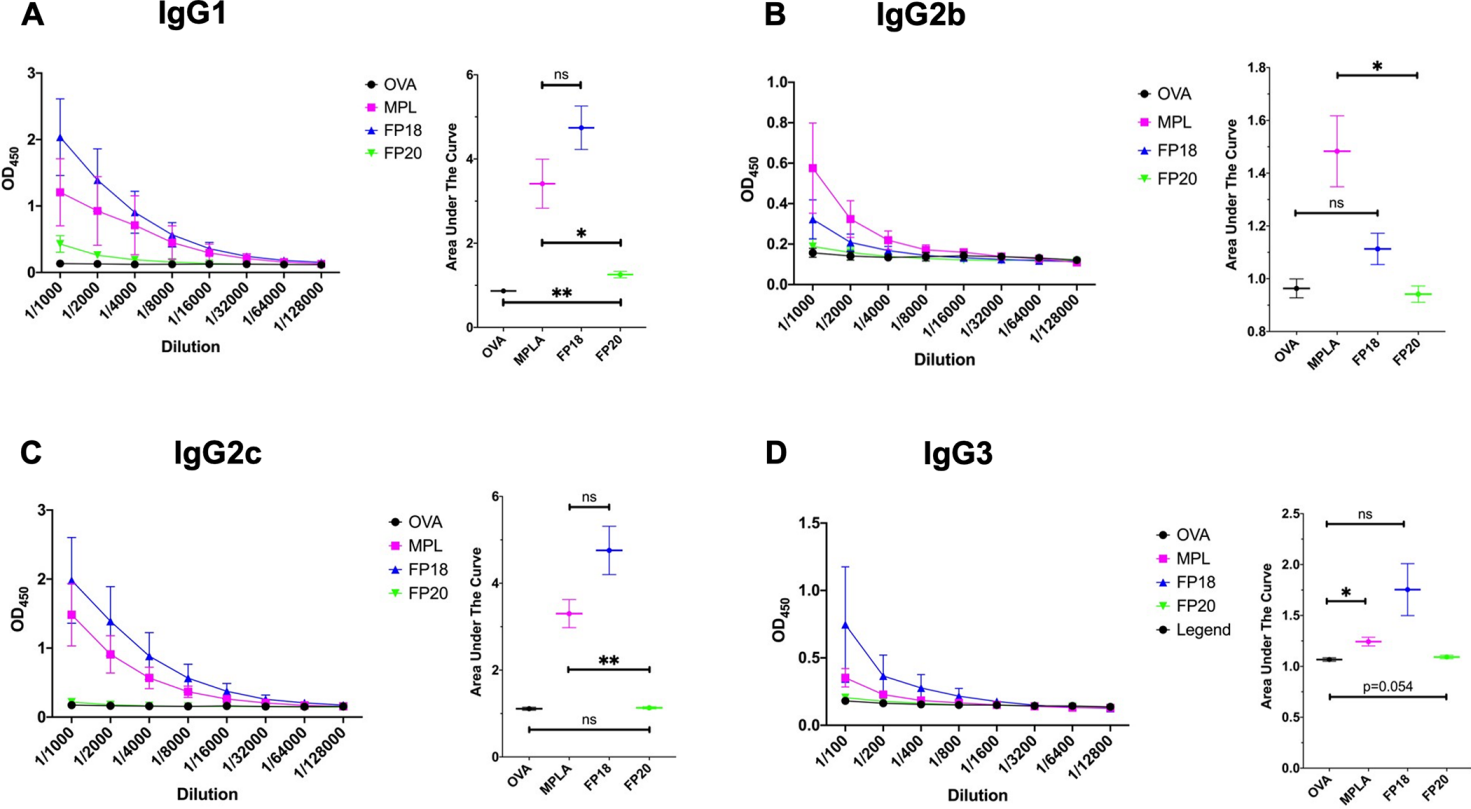
IgG Profile responses to boost OVA immunization 42 days post immunization. (A) IgG1, (B) IgG2b, (C) IgG2c, and (D) IgG3. Values represent mean ± SEM. Brown–Forsythe and Welch one-way ANOVA tests (with an alpha of 0.05) were utilized to compare the areas under each curve. *p < 0.05; **p < 0.01; ***p < 0.001.

MPLA IgG1 titers did not differ significantly from **FP18** and were significantly higher than the **FP20** group (Figure 15A). IgG2b titers of MPLA and **FP18** were not significantly different, and there was no difference between **FP20** and the OVA control group (Figure 15B). MPLA produced significantly higher IgG2c titers than **FP20**, while **FP18** did not differ from the OVA control group (Figure 15C). **FP18** and **FP20** did not significantly differ to OVA in IgG3 production (Figure 15D). Taken together, these data show that **FP20**, while being less active than **FP18**, retains adjuvant activity. Overall liver transaminases data indicate that both **FP18** and **FP20** are non-toxic in this immunization scheme (Supporting Information).

## Conclusions

The development of new vaccine adjuvants has been slow; thus, the discovery of new compounds with adjuvant activity that have straightforward and scalable synthesis and a known mechanism of action is urgent.^19^ We have shown here that **FP20** and its derivatives strongly and selectively stimulate TLR4 and that **FP20** has adjuvant activity. **FP20** shows a potency similar to previously published **FP18**, while having a shorter and more scalable synthesis, together with an increased chemical stability. At the same time, DLS demonstrated that the CMC of **FP20** is lower than that of the parent lipid X, as well as any other synthetic analogue. Related to this, at micromolar concentrations (3.75–15 μM), the formation of SUV-like particles has been observed by DLS, while at higher concentrations (0.8–1.0 mM), LUV or cylindrical vesicles have been visualized by cryo-EM. Thus, low concentrations of **FP20** would be better suited for vaccine formulations. Through MD simulations, it was observed that the α-anomer of **FP20** (α-**FP20**) presents a different packing of the lipophilic chains, which decreases the interaction with TLR4/MD-2, and upstream with the L BP and CD14 proteins, in respect to its β analogue. **FP200**, with two phosphates, shows lower activity, which is consistent with the MD data that shows that one phosphate and an anomeric-β FA chain are optimal for receptor interaction.

Compounds **FP20**–**24** were tested in order to study the effect of the FA chain length in the activity, and the results show that the presence of three C12 chains (**FP20**) or C10 chains (**FP22**) yields better ligands for TLR4/MD-2, according to docking and MD simulations, and have higher biological activity on TDM cells. This observation parallels what we described in the case of TLR4 antagonists with a similar structure.^42^ The computational studies have shown that **FP20** and **FP22** display a cylindrical shape that allows these ligands to accomplish optimal agonist binding properties inside the MD-2 pocket. Conversely, **FP24**, with one C12 and two C10 FA chains, has a different shape, which decreased its polar interactions with the target receptor, presenting a behavior that was mirrored by the lower activity in TDM cells. We therefore observed a relationship between the chemical structure, shape, and FA chain length of compounds FP and their TLR4/MD-2 binding ability.

**FP20**–**22** were further characterized, and their cytokine profile both in PBMCs and in TDM revealed a significative production of TNF and IL-1β. **FP20** did not show to induce NF-κB (p65 subunit) or p-IRF-3 nuclear translocation in immunofluorescence experiments. As these transcriptional factors were not detected in Western blot analysis, we did analyze another important protein in TLR4 signaling, the p38 MAPK.^46^ p38 activation was detected using Western blot, suggesting that it has a role in the downstream transcriptions leading to the synthesis of pro-inflammatory cytokines.

Remarkably, IL-1β production in TDM treated with **FP20** was comparable with the one of the positive control S-LPS, which suggested an important contribution of the NLRP3 inflammasome to the proinflammatory activity of this type of molecules, also reflecting what was observed in the case of **FP18**.^23^ A reduction in IL-1β production in TDM treated with **FP20** after a pre-treatment with NLRP3 inhibitor MCC950 was observed, indicating a role of this protein complex in the mechanism of action of **FP20**. NLRP3 inflammasome activation has been previously described as a mechanism of action of other adjuvants,^47^ and in particular it has been associated with the approved and widely used adjuvants MF59 and Alum.^48^

In vivo data with the OVA antigen confirmed the low toxicity of **FP20** as well as its immunostimulatory ability especially after the first immunization. Taken together, the results described in this work justify further pharmacological development of this type of TLR4 agonists in the context of vaccine adjuvants.

## Experimental Section

### Chemistry

All reagents and solvents were purchased from commercial sources and used without further purifications, unless stated otherwise. Reactions were monitored by thin-layer chromatography (TLC) performed over Silica gel 60 F254 plates (Merck). Flash chromatography purifications were performed on Silica gel 60 60–75 μm from a commercial source or using Biotage Isolera LS Systems.

^1^H and ^13^C NMR spectra were recorded with Bruker Advance 400 with TopSpin software, or with NMR Varian 400 with VnmrJ software. Chemical shifts are expressed in ppm with respect to Me_4_Si; coupling constants are expressed in Hz. The multiplicity in the ^13^C spectra was deducted by APT experiments.

Exact masses were recorded with Agilent 6500 Series Q-TOF LC/MS System. Purity of final compounds was >95% as assessed by quantitative NMR analysis**.**

### Compounds **2a**–**e**

#### 2-Dodecanamido-2-deoxy-α,β-d-glucopyranose

Glucosamine hydrochloride **1** (10 g, 46.5 mmol, 1 eq.) and NaHCO_3_ (10.54 g, 126 mmol, 2.7 eq.) were dissolved in water (120 mL). Then, previously dissolved acyl chloride (11.20 g, 51.2 mmol, 1.1 eq.) in THF (120 mL) was added dropwise to the solution at 0 °C. Reaction was stirred for 5 h at RT; a white precipitate was formed in the reaction flask. Solution was filtered and a white solid was obtained, which was washed with 4 °C water. The solid was resuspended in 75 mL of 0.5 HCl for c.a 30 min and then filtered again and washed with THF. Excess water in the solid was then co-evaporated with toluene under reduced pressure, to obtain the desired products **2a**–**e** as a white powder in 65% yield (11.10 g) as an anomeric mixture. Compounds were used without further purification.

^1^H NMR (400 MHz, DMSO) δ 7.66 (d, *J* = 8.0 Hz, 1H, NHβ), 7.49 (d, *J* = 7.7 Hz, 4H, NHα), 6.44 (d, *J* = 6.2 Hz, 1H, 1-OHβ), 6.39–6.33 (m, 4H, 1-OHα), 4.95–4.91 (m, 5H, H-1α + 6-OHβ), 4.89 (d, *J* = 5.2 Hz, 4H, 4-OHα), 4.79 (d, *J* = 4.8 Hz, 1H, 4-OHβ), 4.59 (d, *J* = 5.1 Hz, 4H, 3-OHα), 4.51 (t, *J* = 5.8 Hz, 1H, 3-OHβ), 4.42 (dt, *J* = 11.5, 5.5 Hz, 5H, H-1β + 6-OHα), 3.67 (dd, *J* = 11.8, 4.6 Hz, 1H, H-3β), 3.62 (dd, *J* = 5.1, 2.1 Hz, 1H), 3.61–3.40 (m, 14H, sugar ring), 3.11 (ddd, *J* = 9.7, 8.2, 5.1 Hz, 4H, H-6α), 3.08–3.03 (m, 2H, H-6β), 2.08 (dt, *J* = 10.9, 7.4 Hz, 10H, CH_2_α chains), 1.47 (q, *J* = 7.0 Hz, 11H, CH_2_β chains), 1.24 (s, 80H, chains bulk), 0.90–0.82 (m, 15H, chain ends).

^13^C NMR (101 MHz, DMSO) δ 173.3, 172.8, 96.1, 91.0, 77.2, 74.7, 72.4, 71.5, 71.3, 70.8, 61.5, 57.5, 54.7, 40.5, 40.3, 40.1, 39.9, 39.7, 39.5, 39.3, 36.1, 35.7, 31.7, 29.5, 29.5, 29.4, 29.4, 29.2, 29.2, 29.1, 25.8, 22.6, 14.4.

HRMS (ESI-Q-TOF): *m*/*z* [M + Na^+^] calculated for C_18_H_35_NNaO_6_^+^: 384.2361. Found: 384.2364.

### Compounds **3a**–**e**

#### 2-Dodecanamido-2-deoxy-6-*O*-*tert*-butyldimethylsilyl-α,β-d-glucopyranose

To a solution of **2a**–**e** (3 g, 8.3 mmol, 1 eq.) and imidazole (850 mg, 12.4 mmol, 1.5 eq.) in dimethyl sulfoxide (166 mL, 0.05 M), a solution of TBDMSCl (1.4 g, 9.1 mmol, 1.1 eq.) in DCM (15 mL) was added dropwise under an inert atmosphere in an ice bath. Subsequently, the solution was allowed to return at room temperature and stirred overnight. The reaction, monitored by TLC (DCM/MeOH 9:1), was then stopped and the solution concentrated under reduced pressure. Then, it was diluted with AcOEt and washed three times with NH_4_Cl. The organic phase thus obtained was dried with Na_2_SO_4_, and the solvent was removed by rotavapor. The crude product thus obtained (3.65 g) was resuspended in EtPet at 0 °C for 30 min. Then, the suspension was filtered under vacuum and the desired compound was recovered as a white solid. After filtration, 3.51 g of compounds **3a**–**e** were obtained, in 85% yield.

^1^H NMR (400 MHz, DMSO) δ 7.62 (d, *J* = 7.9 Hz, 1H; NH), 6.43 (d, *J* = 6.4 Hz, 1H; 1-OH), 4.90 (d, *J* = 6.5 Hz, 1H; 4-OH), 4.77 (d, *J* = 9.1 Hz, 1H; 3-OH), 4.42 (t, *J* = 7.0 Hz, 1H; H-1), 3.86 (d, *J* = 10.8 Hz, 1H; H-6), 3.66 (dd, *J* = 11.0, 4.6 Hz, 1H; H-6), 3.30 (d, *J* = 7.9 Hz, 2H; H-2 + H-3), 3.14–2.98 (m, 2H; H-4 + H-5), 2.06 (t, *J* = 7.4 Hz, 2H CH_2_α chain), 1.48 (s, 2H; CH_2_β chains), 1.24 (s, 20H; chain bulk), 0.94–0.74 (m, 12H 3x chain ends +9x tBu-Si), 0.05 (d, *J* = 3.0 Hz, 6H; Me-Si).

^13^C NMR (101 MHz, DMSO) δ 173.2, 95.9, 77.1, 74.8, 70.8, 63.6, 57.5, 40.6, 40.4, 40.2, 40.0, 39.8, 39.6, 39.4, 36.2, 31.8, 29.5, 29.5, 29.4, 29.4, 29.2, 29.1, 26.4, 25.8, 22.6, 18.6, 14.4, −4.7, −4.7.

HRMS (ESI-Q-TOF): *m*/*z* [M + Na^+^] calculated for C_24_H_49_NNaO_6_Si^+^: 498.3226. Found: 498.3223.

### Compounds **4a**–**e**

#### 1,3-Di-*O*-dodecanoyl-2-dodecanamido-2-deoxy-6-*O*-*tert*-butyldimethylsilyl-β-d-glucopyranose

Compounds **3a**–**e** (2.0 g, 4.2 mmol, 1 eq.) were dissolved in anhydrous THF (84 mL, 0.05 M) under an Ar atmosphere. TEA (2.4 mL, 17.2 mmol, 4.1 eq.) and acyl chloride (2.2 mL, 9.2 mmol, 2.2 eq.) were added dropwise to the solution at −20 °C, and then also 4-dimethylaminopyridine (26 mg, 0.2 mmol, 0.05 eq.) was added. The reaction was slowly allowed to return to 0 °C and stirred over 2 h and then controlled by TLC (EtPet/AcOEt 6:4). Subsequently, the solution was diluted in AcOEt and washed with 1 M HCl. The organic phase thus obtained was dried with Na_2_SO_4_, and the solvent was removed by a rotavapor. The crude product thus obtained (4 g) was purified using Biotage Isolera LS System (Tol/AcOEt 99:1 to 88:12 over 10 CV). After purification, 2.12 g of compounds **4a**–**e** was obtained, in 60% yield.

^1^H NMR (400 MHz, DMSO) δ 7.80 (d, *J* = 9.5 Hz, 1H; NH), 5.56 (d, *J* = 8.9 Hz, 1H; H-1), 5.38 (d, *J* = 5.9 Hz, 1H; 3-OH), 4.92 (dd, *J* = 10.6, 8.6 Hz, 1H; H-3), 3.83 (dd, *J* = 10.4, 5.8 Hz, 2H; H-2 + H-6), 3.76–3.70 (m, 1H; H-6), 3.38 (dd, *J* = 14.3, 8.5 Hz, 2H; H-4 + H-5), 2.30–2.14 (m, 7H; CH_2_α chains), 1.94 (t, *J* = 7.3 Hz, 2H; CH_2_α chain), 1.44 (dd, *J* = 25.9, 6.4 Hz, 10H; CH_2_β chains), 1.24 (d, *J* = 2.4 Hz, 75H; chains bulk), 0.90–0.81 (m, 24H; 9x chain ends +9x tBu-Si), 0.07–(−0.01) (m, 6H; Me-Si).

^13^C NMR (101 MHz, DMSO) δ 174.9, 172.7, 172.3, 171.7, 92.5, 77.5, 75.7, 67.7, 62.54, 52.3, 40.6, 40.4, 40.2, 40.0, 39.8, 39.6, 39.4, 36.1, 34.1, 33.9, 31.8, 31.7, 29.6, 29.5, 29.5, 29.4, 29.4, 29.4, 29.3, 29.2, 29.0, 28.9, 28.8, 26.3, 25.7, 25.0, 24.8, 22.5, 18.5, 14.4, 14.4, −4.7, −4.8.

HRMS (ESI-Q-TOF): *m*/*z* [M + Na^+^] calculated for C_48_H_93_NNaO_8_Si^+^: 862.6567. Found: 862.6569.

### Compounds **5a**–**e**

#### 1,3-Di-*O*-dodecanoyl-2-dodecanamido-2-deoxy-4-*O*-(dibenzyl)phospho-6-*O*-*tert*-butyldimethylsilyl-β-d-glucopyranose

Compounds **4a**–**e** (2.12 g, 2.4 mmol, 1 eq.) and imidazole triflate (1.4 g, 5.4 mmol, 2.25 eq.) were dissolved in DCM (121 mL, 0.02 M) under an inert atmosphere. Dibenzyl *N*,*N*-diisopropylphosphoramidite (1.83 g, 5.3 mmol, 2.2 eq) was added to the solution at 0 °C. The reaction was monitored by TLC (EtPet/acetone 9:1); after 30 min, substrate depletion was detected. Solution was then cooled at −20 °C, and a solution of *meta*-chloroperbenzoic acid (1.66 g, 9.7 mmol, 4 eq.) in 17 mL of DCM was added dropwise. After 30 min, the reaction was allowed to return to RT and left stirring overnight.

After TLC analysis, the reaction was quenched with 15 mL of a saturated NaHCO_3_ solution and concentrated by a rotavapor. The mixture was then diluted in AcOEt and washed three times with a saturated NaHCO_3_ solution and three times with a 1 M HCl solution. The organic phase was recovered and dried with Na_2_SO_4_, and the solvent was removed by rotavapor.

The crude product thus obtained was purified by flash column chromatography (EtPet/acetone 9:1). 2.41 g of pure compounds **5a**–**e** was obtained as a yellow oil in a 91% yield.

^1^H NMR (400 MHz, CDCl_3_) δ 7.34–7.25 (m, 10H; aromatic), 5.61 (d, *J* = 8.7 Hz, 1H; H-1), 5.44 (d, *J* = 9.6 Hz, 1H; NH), 5.16 (dd, *J* = 10.8, 9.1 Hz, 1H; H-3), 5.00 (dd, *J* = 8.1, 2.8 Hz, 2H; CH_2_-Ph), 4.96–4.91 (m, 2H; CH_2_-Ph), 4.53 (q, *J* = 9.2 Hz, 1H; H-4), 4.23 (dt, *J* = 10.8, 9.5 Hz, 1H; H-5), 3.91 (dd, *J* = 11.9, 1.8 Hz, 1H; 1xH-6), 3.78 (dd, *J* = 11.9, 4.6 Hz, 1H; 1xH-6), 3.56 (ddd, *J* = 9.6, 4.4, 1.7 Hz, 1H; H-2), 2.31 (td, *J* = 7.5, 3.5 Hz, 2H; CH_2_α chain), 2.19 (t, *J* = 7.7 Hz, 2H; CH_2_α chain), 2.07–2.01 (m, 2H; CH_2_α chain), 1.61–1.37 (m, 6H; CH_2_β chains), 1.33–1.10 (m, 54H; chain bulk), 0.92–0.83 (m, 18H; 9x chain ends +9x tBu-Si), 0.03–(−0.03) (m, 6H; Me-Si).

^13^C NMR (101 MHz, CDCl_3_) δ 174.4, 172.8, 172.4, 135.5, 128.6, 128.6, 127.9, 127.8, 92.6, 77.3, 77.0, 76.7, 76.2, 76.2, 72.9, 72.9, 69.6, 69.5, 69.5, 61.6, 52.8, 36.8, 34.1, 33.9, 31.9, 29.7, 29.6, 29.5, 29.5, 29.4, 29.4, 29.3, 29.3, 29.1, 29.0, 25.8, 25.6, 24.6, 24.6, 22.7, 18.3, 14.1, −5.2, −5.3.

HRMS (ESI-Q-TOF): *m*/*z* [M + Na^+^] calculated for C_62_H_108_NNaO_11_PSi^+^: 1122.7237. Found: 1123.7234.

### Compounds **6a**–**e**

#### 1,3-Di-*O*-dodecanoyl-2-dodecanamido-2-deoxy-4-*O*-(dibenzyl)phospho-β-d-glucopyranose

Compounds **5a**–**e** (2.41 g, 2.4 mmol, 1 eq.) were dissolved in acetone (48 mL, 0.05 M), and 480 μL (1% v/v) of a 5% v/v solution of H_2_SO_4_ in H_2_O was added at RT. The solution was left stirring for 8 h and monitored by TLC (EtPet/acetone 8:2). After reaction completion, the solution was diluted in AcOEt and washed three times with a saturated NaHCO_3_ solution. The organic phase thus obtained was dried with Na_2_SO_4_, and the solvent was removed by rotavapor. The crude product thus obtained was purified by flash column chromatography (EtPet/acetone 85:15). After purification (2.1 g), compounds **6a**–**e** were obtained as a white solid in a 90% yield.

^1^H NMR (400 MHz, CDCl_3_) δ 7.40–7.27 (m, 10H; aromatics), 5.63 (d, *J* = 8.8 Hz, 1H; H-1), 5.45 (d, *J* = 9.6 Hz, 1H; NH), 5.18 (dd, *J* = 10.7, 9.3 Hz, 1H; H-3), 5.08–4.91 (m, 4H; CH_2_-Ph), 4.54 (q, *J* = 9.5 Hz, 1H; H-4), 4.26 (dd, *J* = 19.9, 9.3 Hz, 1H; H-2), 3.87–3.74 (m, 2H; H-6), 3.47 (d, *J* = 9.7 Hz, 1H; H-5), 2.40–2.24 (m, 2H; CH_2_α chain), 2.10–1.91 (m, 4H; CH_2_α chains), 1.61–1.46 (m, 4H; CH_2_β chains), 1.46–1.33 (m, 2H; CH_2_β chain), 1.33–1.01 (m, 54H; chains bulk), 0.92–0.83 (m, 9H; chains ends).

^13^C NMR (101 MHz, CDCl_3_) δ 174.1, 172.8, 172.5, 129.0, 128.8, 128.7, 128.7, 128.3, 128.0, 92.6, 77.3, 77.0, 76.7, 75.9, 75.9, 72.5, 72.4, 72.1, 72.1, 70.2, 70.2, 70.1, 60.2, 52.8, 36.7, 34.0, 33.7, 31.9, 29.7, 29.6, 29.5, 29.4, 29.4, 29.3, 29.3, 29.3, 29.2, 29.0, 29.0, 25.6, 24.6, 24.5, 22.7, 14.1.

HRMS (ESI-Q-TOF): *m*/*z* [M + Na^+^] calculated for C_59_H_92_NNaO_11_P^+^: 1008.6305. Found: 1008.6309.

### Compounds **FP20**–**24**

#### 1,3-Di-*O*-dodecanoyl-2-dodecanamido-2-deoxy-4-*O*-phospho-β-d-glucopyranose

Compounds **6a**–**e** (50 mg, 0.05 mmol, 1 eq.) were dissolved in a mixture of DCM (2.5 mL) and MeOH (2.5 mL) and put under an Ar atmosphere. The Pd/C catalyst (10 mg, 20% m/m) was then added to the solution. Gases were then removed from the reaction environment, which was subsequently put under a H_2_ atmosphere. The solution was allowed to stir for 2 h, and then H_2_ was removed and reaction monitored by TLC (EtPet/acetone 8:2).

TEA (100 μL, 2.5% v/v) was then added to the reaction, which was stirred for 15 min. The solution was subsequently filtered on syringe filters PALL 4549 T Acrodisc 25 mm with a GF/0.45 μm Nylon to remove the Pd/C catalyst, and solvents were evaporated by a rotavapor. The crude product was resuspended in a DCM/MeOH solution, and IRA 120 H^+^ was added. After 30 min of stirring, IRA 120 H^+^ was filtered, solvents were removed by a rotavapor, the crude was resuspended in DCM/MeOH, and IRA 120 Na^+^ was added. After 30 min stirring, IRA 120 Na^+^ was filtered and solvents were removed by a rotavapor.

The crude product was purified through reverse chromatography employing a C4-functionalized column (PUREZZA-Sphera Plus Standard Flash Cartridge C4—25 μm, size 25 g) in the Biotage Isolera LS System (gradient: H_2_O/THF 70:30 to 15:85 over 10 CV with 1% of an aqueous solution of Et_3_NHCO_3_ at pH 7.4). 45 mg of **FP20–24** was obtained as a white powder in a quantitative yield.

### Compound **FP20**

^1^H NMR (400 MHz, CD_3_OD) δ 5.75 (d, *J* = 8.9 Hz, 1H; H-1), 5.28 (t, *J* = 9.8 Hz, 1H; H-3), 4.28 (q, *J* = 9.7 Hz, 1H; H-4), 4.06 (t, *J* = 9.6 Hz, 1H; H-2), 3.89–3.74 (m, 2H; H-6), 3.62 (t, *J* = 9.2 Hz, 1H; H-5), 2.42–2.25 (m, 4H; CH_2_α chains), 2.09 (t, *J* = 7.6 Hz, 2H; CH_2_α chain), 1.56 (d, *J* = 6.4 Hz, 7H; CH_2_β chains), 1.29 (s, 53H; chain bulk), 0.90 (t, *J* = 6.6 Hz, 9H; chain ends).

^13^C NMR (101 MHz, CD_3_OD) δ 174.7, 173.3, 172.0, 92.2, 76.2, 72.8, 72.2, 72.1, 60.3, 52.8, 48.2, 48.0, 47.8, 47.6, 47.4, 47.2, 47.0, 36.0, 33.6, 33.6, 31.7, 31.7, 29.4, 29.4, 29.4, 29.4, 29.3, 29.2, 29.2, 29.1, 29.1, 29.0, 29.0, 28.9, 28.7, 25.6, 24.4, 22.3, 13.0.

HRMS (ESI-Q-TOF): *m*/*z* [M^–^] calculated for C_42_H_80_NO_11_P^–^: 805.5469. Found: 805.5472.

### Compound **FP21**

^1^H NMR (400 MHz, CD_3_OD) δ 5.78 (d, *J* = 8.8 Hz, 1H; H-1), 5.31 (dd, *J* = 10.6, 9.1 Hz, 1H; H-3), 4.31 (q, *J* = 9.7 Hz, 1H; H-4), 4.09 (dd, *J* = 10.6, 8.8 Hz, 1H; H-2), 3.84 (tdd, *J* = 12.4, 8.2, 3.2 Hz, 3H; H-6), 3.63 (ddd, *J* = 10.0, 4.5, 2.3 Hz, 1H; H-5), 2.45–2.27 (m, 5H; CH_2_α chains), 2.12 (t, *J* = 7.6 Hz, 2H; CH_2_α chain), 1.67–1.51 (m, 8H; CH_2_β chains), 1.31 (s, 74H; chain bulk), 0.92 (t, *J* = 6.5 Hz, 12H; chain ends).

^13^C NMR (101 MHz, CD_3_OD) δ 174.7, 173.3, 172.0, 92.2, 76.2, 72.8, 72.3, 60.7, 60.3, 52.8, 36.1, 33.6, 33.6, 31.7, 31.7, 29.5, 29.4, 29.4, 29.3, 29.3, 29.2, 29.1, 29.1, 28.9, 28.7, 25.6, 24.4, 22.3, 22.3, 13.0.

HRMS (ESI-Q-TOF): *m*/*z* [M^–^] calculated for C_46_H_86_NO_11_P^–^: 859.5949. Found: 859.5951.

### Compound **FP22**

^1^H NMR (400 MHz, CD_3_OD) δ 5.75 (d, *J* = 8.9 Hz, 1H; H-1), 5.28 (dd, *J* = 10.4, 9.3 Hz, 1H; H-3), 4.27 (q, *J* = 9.8 Hz, 1H; H-4), 4.10–4.01 (m, 1H; H-2), 3.86–3.77 (m, 2H; H-6), 3.60 (dd, *J* = 9.8, 3.3 Hz, 1H; H-5), 2.41–2.24 (m, 4H; CH_2_α chains), 2.11–2.06 (m, 2H; CH_2_α chains), 1.55 (dd, *J* = 13.5, 6.8 Hz, 6H; CH_2_β chains), 1.29 (s, 48H; chain bulk), 0.97–0.83 (m, 9H; chain ends).

^13^C NMR (101 MHz, CD_3_OD) δ 174.8, 174.7, 173.9, 173.3, 172.0, 92.2, 91.3, 76.2, 76.2, 72.9, 72.8, 72.8, 72.1, 72.0, 71.4, 70.4, 60.6, 60.3, 52.8, 52.3, 48.2, 48.0, 47.8, 47.6, 47.4, 47.2, 47.0, 46.6, 36.0, 35.7, 33.8, 33.7, 33.6, 33.5, 31.6, 31.6, 31.6, 29.2, 29.2, 29.1, 29.1, 29.1, 29.0, 29.0, 29.0, 28.9, 28.8, 28.7, 25.6, 25.6, 24.7, 24.4, 24.4, 22.3, 13.0, 7.8.

HRMS (ESI-Q-TOF): *m*/*z* [M^–^] calculated for C_36_H_66_NO_11_P^–^: 719.4384. Found: 719.4381.

### Compound **FP23**

^1^H NMR (400 MHz, CD_3_OD) δ 5.78 (d, *J* = 8.8 Hz, 1H; H-1), 5.30 (dd, *J* = 10.6, 9.1 Hz, 1H; H-3), 4.31 (q, *J* = 9.6 Hz, 1H; H-4), 4.09 (dd, *J* = 10.6, 8.8 Hz, 1H; H-2), 3.90–3.78 (m, 2H; H-6), 3.62 (ddd, *J* = 9.9, 4.3, 2.4 Hz, 1H; H-5), 2.46–2.28 (m, 5H; CH_2_α chains), 2.12 (t, *J* = 7.6 Hz, 2H; CH_2_α chains), 1.66–1.50 (m, 7H; CH_2_β chains), 1.31 (s, 70H; chain bulk), 0.92 (t, *J* = 6.6 Hz, 10H; chain ends).

^13^C NMR (101 MHz, CD_3_OD) δ 174.7, 173.3, 172.0, 92.2, 76.2, 72.8, 72.2, 60.3, 36.1, 33.6, 31.7, 29.4, 29.1, 28.7, 25.6, 24.4, 22.3, 13.0.

HRMS (ESI-Q-TOF): *m*/*z* [M^–^] calculated for C_48_H_90_NO_11_P^–^: 887.6262. Found: 887.6265.

### Compound **FP24**

^1^H NMR (400 MHz, CD_3_OD) δ 5.79 (d, *J* = 8.8 Hz, 1H; H-1), 5.31 (dd, *J* = 10.7, 9.0 Hz, 1H; H-3), 4.31 (q, *J* = 9.7 Hz, 1H; H-4), 4.09 (dd, *J* = 10.6, 8.8 Hz, 1H; H-2), 3.90–3.78 (m, 2H; H-6), 3.63 (ddd, *J* = 9.8, 4.1, 2.4 Hz, 1H; H-5), 2.46–2.26 (m, 5H; CH_2_α chains), 2.12 (t, *J* = 7.6 Hz, 2H; CH_2_α chains), 1.58 (dq, *J* = 17.8, 6.6 Hz, 6H; CH_2_β chains), 1.40–1.25 (m, 49H; chain bulk), 0.97–0.87 (m, 10H; chain ends).

^13^C NMR (101 MHz, CD_3_OD) δ 174.7, 173.3, 172.0, 92.1, 76.2, 76.1, 72.8, 72.7, 72.1, 72.0, 60.3, 52.8, 36.0, 33.6, 33.5, 31.7, 31.7, 29.5, 29.4, 29.4, 29.4, 29.3, 29.3, 29.2, 29.2, 29.1, 29.1, 29.0, 29.0, 28.9, 28.8, 25.6, 24.4, 22.4, 13.1.

HRMS (ESI-Q-TOF): *m*/*z* [M^–^] calculated for C_38_H_70_NO_11_P^–^: 747.4697. Found: 747.4701.

### Compound **7**

#### 1,3-Di-*O*-dodecanoyl-2-dodecanamido-2-deoxy-6-*O*-*tert*-butyldimethylsilyl-α-d-glucopyranose

Compound **3a** (2.0 g, 4.2 mmol, 1 eq.) was dissolved in anhydrous THF (84 mL, 0.05 M) under an Ar atmosphere. TEA (2.4 mL, 17.2 mmol, 4.1 eq.), 4-dimethylaminopyridine (1.1 g, 9.2 mmol, 2.2 eq.), and lauroyl chloride (2.2 mL, 9.2 mmol, 2.2 eq.) were added to the solution at 0 °C. The reaction was subsequently heated to 30 °C and stirred over 2 h, and then controlled by TLC (EtPet/AcOEt 6:4). Subsequently, the solution was diluted in AcOEt and washed with 1 M HCl. The organic phase thus obtained was dried with Na_2_SO_4_, and the solvent was removed by a rotavapor. The crude product thus obtained (4 g) was purified using Biotage Isolera LS System (Tol/AcOEt 99:1 to 88:12 over 10 CV). After purification, 1.59 g of compound **7** was obtained, in 45% yield.

^1^H NMR (400 MHz, CDCl_3_) δ 6.13 (d, *J* = 3.6 Hz, 1H, H-1), 5.60 (d, *J* = 8.6 Hz, 1H, NH), 5.16 (dd, *J* = 11.1, 9.2 Hz, 1H, H-3), 4.30 (ddd, *J* = 11.2, 8.7, 3.6 Hz, 1H, H-3), 3.93 (dd, *J* = 9.8, 3.7 Hz, 1H, H-6a), 3.86 (t, *J* = 9.1 Hz, 1H, H-4), 3.75 (dd, *J* = 9.9, 6.6 Hz, 1H, H-6b), 3.72–3.68 (m, 1H, H-5), 2.44–2.27 (m, 4H, CH_2_α chains), 2.08 (td, *J* = 7.4, 2.5 Hz, 2H, CH_2_α chain), 1.72–1.57 (m, 6H, CH_2_β chains), 1.56–1.47 (m, 2H, CH_2_β chains), 1.36–1.19 (m, 53H, chain bulk), 0.91–0.83 (m, 20H, 9x chain ends +9x tBu-Si), 0.10–0.06 (m, 6H, Me-Si).

^13^C NMR (101 MHz, CDCl_3_) δ 175.1, 172.8, 171.4, 135.6, 135.5, 135.5, 135.5, 128.6, 128.6, 127.9, 127.8, 90.2, 73.1, 73.1, 72.9, 72.8, 70.9, 69.6, 69.5, 69.5, 69.4, 61.4, 51.3, 36.5, 34.2, 34.0, 31.9, 29.6, 29.6, 29.5, 29.4, 29.4, 29.3, 29.3, 29.3, 29.2, 29.1, 25.8, 25.5, 24.8, 24.6, 23.8, 22.7, 18.3, 14.1, −5.3, −5.3.

HRMS (ESI-Q-TOF): *m*/*z* [M + Na^+^] calculated for C_48_H_93_NNaO_8_Si^+^: 862.6567. Found: 862.6565.

### Compound **8**

#### 1,3-Di-*O*-dodecanoyl-2-dodecanamido-2-deoxy-4-*O*-(dibenzyl)phospho-6-*O*-*tert*-butyldimethylsilyl-α-d-glucopyranose

Compound **7** (1.59 g, 1.8 mmol, 1 eq.) and imidazole triflate (1.0 g, 4.0 mmol, 2.25 eq.) were dissolved in DCM (90 mL, 0.02 M) under an inert atmosphere. Dibenzyl *N*,*N*-diisopropylphosphoramidite (1.38 g, 4.0 mmol, 2.2 eq) was added to the solution at 0 °C. The reaction was monitored by TLC (EtPet/acetone 9:1); after 30 min, substrate depletion was detected. The solution was then cooled at −20 °C, and a solution of meta-chloroperbenzoic acid (1.24 g, 7.2 mmol, 4 eq.) in 12 mL of DCM was added dropwise. After 30 min, the reaction was allowed to return to RT and left to stir overnight.

After TLC analysis, reaction was quenched with 15 mL of a saturated NaHCO_3_ solution and concentrated by a rotavapor. The mixture was then diluted in AcOEt and washed three times with a saturated NaHCO_3_ solution and three times with a 1 M HCl solution. The organic phase was recovered and dried with Na_2_SO_4_, and the solvent was removed by rotavapor.

The crude product thus obtained was purified by flash column chromatography (EtPet/acetone 9:1). 1.80 g of pure compound **8** was obtained as a yellow oil in 91% yield.

^1^H NMR (400 MHz, CDCl_3_) δ 7.37–7.26 (m, 10H, aromatics), 6.18 (d, *J* = 3.6 Hz, 1H, H-1), 5.53 (d, *J* = 8.7 Hz, 1H, NH), 5.32 (dd, *J* = 11.1, 9.2 Hz, 1H, H-3), 5.01 (dd, *J* = 8.1, 3.3 Hz, 2H, CH_2_-Ph), 4.95 (dd, *J* = 7.7, 0.9 Hz, 2H, CH_2_-Ph), 4.59 (q, *J* = 9.2 Hz, 1H, H-4), 4.31 (ddd, *J* = 11.1, 8.7, 3.7 Hz, 1H, H-2), 3.88–3.74 (m, 3H, H-5, H-6), 2.43–2.35 (m, 2H, CH_2_α chain), 2.20 (t, *J* = 7.7 Hz, 2H, CH_2_α chain), 2.06 (td, *J* = 7.4, 2.4 Hz, 2H, CH_2_α chain), 1.65 (p, *J* = 7.4 Hz, 2H, CH_2_β chain), 1.58–1.38 (m, 5H, CH_2_β chains), 1.37–1.12 (m, 51H, chains bulk), 0.93–0.81 (m, 19H, 9x chain ends +9x tBu-Si), 0.01 (d, *J* = 7.4 Hz, 6H, Me-Si).

^13^C NMR (101 MHz, CDCl_3_) δ 175.1, 172.8, 171.4, 135.6, 135.5, 135.5, 135.5, 128.6, 128.6, 128.2, 127.9, 127.8, 90.2, 73.1, 73.1, 72.9, 72.8, 70.9, 69.6, 69.5, 69.5, 69.4, 61.4, 51.3, 36.5, 34.2, 34.0, 31.9, 29.6, 29.6, 29.5, 29.5, 29.4, 29.4, 29.4, 29.3, 29.3, 29.3, 29.2, 29.2, 29.1, 25.8, 25.5, 24.8, 24.6, 23.8, 22.7, 18.3, 14.1, −5.3, −5.3.

HRMS (ESI-Q-TOF): *m*/*z* [M + Na^+^] calculated for C_62_H_108_NNaO_11_PSi^+^: 1122.7237. Found: 1123.7239.

### Compound **9**

#### 1,3-Di-*O*-dodecanoyl-2-dodecanamido-2-deoxy-4-*O*-(dibenzyl)phospho-α-d-glucopyranose

Compound **8** (1.80 g, 1.6 mmol, 1 eq.) was dissolved in acetone (32 mL, 0.05 M), and 320 μL (1% v/v) of a 5% v/v solution of H_2_SO_4_ in H_2_O was added at RT. The solution was left to stir for 8 h and monitored by TLC (EtPet/acetone 8:2). After reaction completion, the solution was diluted in AcOEt and washed three times with a saturated NaHCO_3_ solution. The organic phase thus obtained was dried with Na_2_SO_4_, and the solvent was removed by a rotavapor. The crude product thus obtained was purified by flash column chromatography (EtPet/acetone 85:15). After purification (1.4 g), compound **9** was obtained as a white solid in 90% yield.

^1^H NMR (400 MHz, CDCl_3_) δ 7.40–7.28 (m, 10H, aromatics), 6.17 (d, *J* = 3.7 Hz, 1H, H-1), 5.45 (d, *J* = 8.9 Hz, 1H, NH), 5.32–5.25 (m, 1H, H-3), 5.09–4.94 (m, 4H, CH_2_-Ph), 4.58 (q, *J* = 9.5 Hz, 1H, H-4), 4.40 (ddd, *J* = 11.0, 8.9, 3.7 Hz, 1H, H-2), 3.84 (dd, *J* = 13.2, 2.8 Hz, 1H, H-6a), 3.78–3.72 (m, 1H, H-6b), 3.72–3.67 (m, 1H, H-5), 2.38 (dd, *J* = 7.9, 7.1 Hz, 2H, CH_2_α chain), 2.05 (dtd, *J* = 15.3, 8.0, 4.3 Hz, 4H, CH_2_α chains), 1.65 (p, *J* = 7.3 Hz, 2H, CH_2_β chain), 1.52 (td, *J* = 8.3, 3.9 Hz, 2H, CH_2_β chain), 1.45–1.36 (m, 2H, CH_2_β chain), 1.35–1.06 (m, 50H, chain bulk), 0.88 (td, *J* = 6.9, 2.5 Hz, 9H, chain ends).

^13^C NMR (101 MHz, CDCl_3_) δ 174.7, 172.7, 171.4, 135.2, 135.2, 135.1, 135.1, 128.9, 128.8, 128.7, 128.7, 128.2, 127.9, 90.4, 77.3, 77.0, 76.7, 72.7, 72.7, 72.1, 72.1, 70.5, 70.5, 70.2, 70.1, 70.1, 70.0, 60.2, 51.1, 36.5, 34.1, 33.8, 31.9, 29.6, 29.6, 29.6, 29.5, 29.5, 29.3, 29.3, 29.3, 29.2, 29.1, 29.0, 25.5, 24.8, 24.6, 22.7, 14.1.

HRMS (ESI-Q-TOF): *m*/*z* [M + Na^+^] calculated for C_59_H_92_NNaO_11_P^+^: 1008.6305. Found: 1008.6308.

### Compound α-**FP20**

#### 1,3-Di-*O*-dodecanoyl-2-dodecanamido-2-deoxy-4-*O*-phospho-α-d-glucopyranose

Compound **9** (50 mg, 0.05 mmol, 1 eq.) was dissolved in a mixture of DCM (2.5 mL) and MeOH (2.5 mL) and put under an Ar atmosphere. The Pd/C catalyst (10 mg, 20% m/m) was then added to the solution. Gases were then removed in the reaction environment, which was subsequently put under a H_2_ atmosphere. The solution was allowed to stir for 2 h, and then H_2_ was removed and reaction monitored by TLC (EtPet/acetone 8:2).

TEA (100 μL, 2% v/v) was then added to the reaction, which was stirred for 15 min. The solution was subsequently filtered on syringe filters PALL 4549 T Acrodisc 25 mm with a GF/0.45 μm Nylon to remove the Pd/C catalyst, and solvents were evaporated by a rotavapor. The crude product was resuspended in DCM/MeOH solution, and IRA 120 H^+^ was added. After 30 min of stirring, IRA 120 H^+^ was filtered, solvents were removed by a rotavapor, the crude product was resuspended in DCM/MeOH, and IRA 120 Na^+^ was added. After 30 min of stirring, IRA 120 Na^+^ was filtered and solvents were removed by a rotavapor.

The crude product was purified through reverse chromatography employing a C4-functionalized column (PUREZZA-Sphera Plus Standard Flash Cartridge C4—25 μm, size 25 g) in the Biotage Isolera LS System (gradient: H_2_O/THF 70:30 to 15:85 over 10 CV with 1% of an aqueous solution of Et_3_NHCO_3_ at pH 7.4). 45 mg of α-**FP20** was obtained as a white powder in quantitative yield.

^1^H NMR (400 MHz, CD_3_OD) δ 6.13 (d, *J* = 3.8 Hz, 1H, H-1), 5.36 (dd, *J* = 11.0, 9.1 Hz, 1H, H-3), 4.42–4.33 (m, 2H, H-2 and H-4), 3.88–3.83 (m, 1H, H-5), 3.83–3.78 (m, 2H, H-6), 2.51 (t, *J* = 7.4 Hz, 2H, CH_2_α chain), 2.47–2.30 (m, 2H, CH_2_α chain), 2.17 (dd, *J* = 9.6, 5.5 Hz, 2H, CH_2_α chain), 1.68 (dt, *J* = 13.9, 6.9 Hz, 2H, CH_2_β chain), 1.64–1.52 (m, 4H, CH_2_β chain), 1.41–1.25 (m, 58H, chain bulk), 0.96–0.89 (m, 9H, chain ends).

^13^C NMR (101 MHz, CD_3_OD) δ 175.1, 173.6, 172.2, 90.1, 73.4, 73.4, 72.3, 72.2, 70.6, 60.6, 50.7, 48.2, 48.0, 47.8, 47.6, 47.4, 47.2, 47.0, 35.5, 33.7, 33.3, 31.7, 31.7, 29.4, 29.4, 29.4, 29.4, 29.4, 29.,3 29.3, 29.2, 29.2, 29.1, 29.0, 29.0, 28.8, 25.6, 24.4, 24.4, 22.3, 13.0.

HRMS (ESI-Q-TOF): *m*/*z* [M^–^] calculated for C_42_H_80_NO_11_P^–^: 805.5469. Found: 805.5463.

### Compound **10**

#### 1,3-Di-*O*-dodecanoyl-2-dodecanamido-2-deoxy-4,6-di-*O*-(dibenzyl)phospho-β-d-glucopyranose

Compound **6a** (2.36 g, 2.4 mmol, 1 eq.) and imidazole triflate (1.4 g, 5.4 mmol, 2.25 eq.) were dissolved in DCM (121 mL, 0.02 M) under an inert atmosphere. Dibenzyl *N*,*N*-diisopropylphosphoramidite (1.83 g, 5.3 mmol, 2.2 eq) was added to the solution at 0 °C. The reaction was monitored by TLC (EtPet/acetone 9:1); after 30 min, substrate depletion was detected. The solution was then cooled at −20 °C, and meta-chloroperbenzoic acid (1.66 g, 9.7 mmol, 4 eq.), dissolved in 17 mL of DCM, was added dropwise. After 30 min, the reaction was allowed to return to RT and left to stir overnight.

After TLC analysis, the reaction was quenched with 15 mL of a saturated NaHCO_3_ solution and concentrated by rotavapor. The mixture was then diluted in AcOEt and washed three times with a saturated NaHCO_3_ solution and three times with a 1 M HCl solution. The organic phase was recovered and dried with Na_2_SO_4_, and the solvent was removed by a rotavapor.

The crude product thus obtained was purified by flash column chromatography (EtPet/acetone 9:1). 2.41 g of pure compound **10** was obtained as a yellow oil in 91% yield.

^1^H NMR (400 MHz, CDCl_3_) δ 7.33–7.18 (m, 21H; aromatics), 5.66 (d, *J* = 8.8 Hz, 1H; H-1), 5.51 (d, *J* = 9.5 Hz, 1H; NH), 5.18 (dd, *J* = 10.6, 9.2 Hz, 1H; H-3), 5.02 (dd, *J* = 10.8, 3.3 Hz, 4H; CH_2_-Ph), 5.00–4.95 (m, 2H; CH_2_-Ph), 4.94–4.88 (m, 2H; CH_2_-Ph), 4.49–4.43 (m, 1H; H-4), 4.42–4.36 (m, 1H; H-6), 4.25 (dd, *J* = 19.8, 9.3 Hz, 1H; H-2), 4.16 (ddd, *J* = 11.8, 7.1, 5.0 Hz, 1H; H-6), 3.74 (dd, *J* = 9.5, 4.2 Hz, 1H; H-5), 2.19 (dt, *J* = 15.9, 7.0 Hz, 5H; CH_2_α chains), 2.07–2.01 (m, 2H; CH_2_α chain), 1.49 (dt, *J* = 14.0, 7.1 Hz, 4H; CH_2_β chains), 1.45–1.36 (m, 2H; CH_2_β chain), 1.34–1.11 (m, 54H; chain bulk), 0.88 (t, *J* = 6.8 Hz, 10H; chain ends).

^13^C NMR (101 MHz, CDCl_3_) δ 174.2, 172.8, 172.2, 135.8, 135.7, 135.4, 135.3, 135.2, 128.7, 128.6, 128.6, 128.6, 128.5, 128.5, 128.5, 128.4, 128.0, 128.0, 128.0, 128.0, 92.4, 74.1, 72.6, 72.5, 72.4, 72.4, 69.9, 69.9, 69.8, 69.7, 69.4, 69.4, 69.3, 65.3, 52.7, 36.7, 33.9, 31.9, 29.6, 29.5, 29.5, 29.5, 29.5, 29.4, 29.4, 29.4, 29.4, 29.3 29.2, 29.2, 29.1, 25.6, 24.6, 24.4, 22.7, 14.1.

HRMS (ESI-Q-TOF): *m*/*z* [M + Na^+^] calculated for C_70_H_105_NNaO_14_P_2_^+^: 1268.6907. Found: 1268.6908.

### Compound **FP200**

#### 1,3-Di-*O*-dodecanoyl-2-dodecanamido-2-deoxy-4,6-di-*O*-phospho-β-d-glucopyranose

Compound **100** (57 mg, 0.05 mmol, 1 eq.) was dissolved in a mixture of DCM (2.5 mL) and MeOH (2.5 mL) and put under an Ar atmosphere. The Pd/C catalyst (10 mg, 20% m/m) was then added to the solution. Gases were then removed in the reaction environment, which was subsequently put under a H_2_ atmosphere. The solution was allowed to stir for 2 h; then, H_2_ was removed and reaction monitored by TLC (EtPet/acetone 8:2).

TEA (100 μL, 2% v/v) was then added to the reaction, which was stirred for 15 min. The solution was subsequently filtered on syringe filters PALL 4549 T Acrodisc 25 mm with a GF/0.45 μm Nylon to remove the Pd/C catalyzer, and solvents were evaporated by a rotavapor. The crude product was resuspended in a DCM/MeOH solution, and IRA 120 H^+^ was added. After 30 min of stirring, IRA 120 H^+^ was filtered, solvents were removed by a rotavapor, the crude product was resuspended in DCM/MeOH, and IRA 120 Na^+^ was added. After 30 min of stirring, IRA 120 Na^+^ was filtered and solvents were removed by a rotavapor. 45 mg of **FP200** was obtained as a white powder in a quantitative yield.

^1^H NMR (400 MHz, CD_3_OD) δ 5.77 (d, *J* = 8.8 Hz, 1H; H-1), 5.32–5.23 (m, 1H; H-3), 4.39 (dd, *J* = 18.9, 9.5 Hz, 1H; H-4), 4.21 (d, *J* = 9.7 Hz, 3H; H-6), 4.10–4.00 (m, 1H; H-2), 3.80 (d, *J* = 9.2 Hz, 1H; H-5), 2.44–2.24 (m, 6H; CH_2_α chains), 2.09 (t, *J* = 7.6 Hz, 2H; CH_2_α chain), 1.55 (dd, *J* = 13.5, 6.9 Hz, 10H; CH_2_β chains), 1.39–1.24 (m, 79H; chain bulk), 0.96–0.82 (m, 33H; chain ends).

^13^C NMR (101 MHz, CD_3_OD) δ 174.9, 173.8, 91.2, 72.9, 72.8, 71.2, 69.0, 68.9, 68.8, 64.7, 64.6, 52.1, 48.2, 48.0, 47.8, 47.6, 47.4, 47.2, 47.0, 36.1, 35.6, 33.8, 33.6, 33.6, 33.4, 31.7, 31.6, 29.3, 29.2, 29.2, 29.2, 29.1, 29.1, 29.0, 29.0, 28.8, 28.8, 25.7, 25.6, 24.7, 24.6, 24.4, 22.4, 22.3, 13.1, 13.0, 7.8.

HRMS (ESI-Q-TOF): *m*/*z* [M^–^] calculated for C_42_H_81_NO_14_P_2_^–^: 885.5132. Found: 885.5133.

## Computational Methods

### Computational Studies of TLR4 in Complex with **β**-**FP20**, **β**-**FP22**, and **β**-**FP24**

#### Macromolecule Preparation

3D coordinates from the X-ray structure of the human (TLR4/MD-2/*Escherichia coli* LPS)2 ectodomain (PDB ID 3FXI)1 were retrieved from the Protein Data Bank (www.rcsb.org). Solvent, ligands, and ions were removed. Hydrogen atoms were added to the X-ray structure using the preprocessing tool of the Protein Preparation Wizard of the Maestro package.^49^ The protein structure went through a restrained minimization under the OPLS3 force field^50^ with a convergence parameter to RMSD for heavy atoms kept default at 0.3 Å.

#### Construction and Optimization of the Ligands

The 3D structures of the FP ligands (**FP20**, **FP22**, and **FP24**) were built with PyMOL molecular graphics and modeling package^51^ using as a template *E. coli* lipid A (PDB ID 3FXI)1 with the builder tool implemented in PyMOL. The resulting structures were first refined at the AM1 level of theory and then optimized at the Hartree–Fock (HF) level (HF/6-311G**) with Gaussian09.^52^

#### All-Atom Parametrization of the Ligands

The parameters of the ligands needed for MD simulations were obtained using the standard Antechamber procedure implemented in Amber16.^53^ The partial charges were derived from the HF calculations and formatted for AmberTools15 and Amber16 with Antechamber,^54^ using RESP charges^55^ and assigning the general Amber force field (GAFF) atom types.^56^ Later, the atom types of the saccharide atoms in FP compounds were changed to the GLYCAM06 force field^57^ atom types, and the atoms constituting the lipid chains to the Lipid14 force field^58^ atom types.

#### MD Simulations of FP Compounds in Water

FP structures were subjected to MD refinement in aqueous solvent, prior to docking. The structures were submitted to all-atom MD simulations during 100 ns in the Amber16 suite.^53^ The simulation box was designed such as the edges were distant by at least 10 Å of any atom. The system was solvated with the TIP3P water molecules model. Na + ions were added to counterbalance the eventual charges of the FP molecules. All the simulations were performed with the same equilibration and production protocol. First, the system was submitted to 1000 steps of the steepest descent algorithm followed by 7000 steps of the conjugate gradient algorithm. A 100 kcal·mol^–1^·A-2 harmonic potential constraint was applied to the ligand. In the subsequent steps, the harmonic potential was progressively lowered (respectively to 10, 5, and 2.5 kcal·mol^–1^·A-2) for 600 steps of the conjugate gradient algorithm each time, and then the whole system was minimized uniformly. Next, the system was heated from 0 to 100 K using the Langevin thermostat in the canonical ensemble (NVT) while applying a 20 kcal·mol^–1^·A-2 harmonic potential restraint on the protein and the ligand. Finally, the system was heated up from 100 to 300 K in the isothermal-isobaric ensemble (NPT) under the same restraint condition as the previous step, followed by simulation for 100 ps with no harmonic restraint applied. At this point, the system was ready for the production run, which was performed using the Langevin thermostat under the NPT ensemble, at a 2 fs time step. Long-range electrostatics were calculated using the particle mesh Ewald method.^59^

#### Docking Calculations

To avoid the limitation of using only one scoring function, AutoDock Vina 1.1.2^32^ and AutoDock 4.2^33^ were used for the docking of the FP compounds (**FP20**, **FP22**, and **FP24**) in the TLR4 agonist X-ray structure from PDB ID 3FXI. Preliminarily docked poses were obtained with AutoDock Vina, and the best-predicted docked poses were redocked with AutoDock 4. The AutoDockTools 1.5.6 program^33^ was used to assign the Gasteiger–Marsili empirical atomic partial charges to the atoms of both the ligands and the receptor. Non-polar hydrogens were merged for the ligands. The structures of the receptor and the ligands were set rigid and flexible, respectively. In AutoDock 4.2, the Lamarckian evolutionary algorithm was selected and all parameters were kept default except for the number of genetic algorithm runs that was set to 100 to enhance the sampling. The box spacing was set to the default values of 1 Å in AutoDock Vina and 0.375 Å in AutoDock 4. The size of the box was set to 33.00, 40.50, and 35.25 Å in the *x*-, *y*-, *z*-axes, respectively, with the box center located equidistant to the mass center of residues Arg90 (MD-2), Lys122 (MD-2), and Arg264 (TLR4), in both docking programs. The structure of the receptor was always kept rigid, whereas the structure of the ligands was set partially flexible by providing freedom to some carefully selected rotatable bonds.

### MD Simulations of TLR4/Ligand Complexes

Selected docked complexes were submitted to all-atom MD simulations during 200 ns in the Amber16 suite.^53^ The protein was described by the ff14SB all-atom force field.^60^ For the FP ligands, the monosaccharide backbone was described using the GLYCAM06 force field,^57^ and the lipid chains with the Lipid14 force field.^58^ The simulation box was designed such as the edges were distant by at least 10 Å of any atom. The systems were solvated with the TIP3P water molecules model. Na+ ions were added to counterbalance the eventual charges of the protein–ligand systems when needed. All the simulations were performed with the same equilibration and production protocol. First, the system was submitted to 1000 steps of the steepest descent algorithm followed by 7000 steps of the conjugate gradient algorithm. A 100 kcal·mol^–1^·A-2 harmonic potential constraint was applied to both the proteins and the ligand. In the subsequent steps, the harmonic potential was progressively lowered (respectively to 10, 5, and 2.5 kcal·mol^–1^·A-2) for 600 steps of the conjugate gradient algorithm each time, and then the whole system was minimized uniformly. Next, the system was heated from 0 to 100 K using the Langevin thermostat in the canonical ensemble (NVT) while applying a 20 kcal·mol^–1^·A-2 harmonic potential restraint on the protein and the ligand. Finally, the system was heated up from 100 to 300 K in the isothermal-isobaric ensemble (NPT) under the same restraint condition as the previous step, followed by simulation for 100 ps with no harmonic restraint applied. At this point, the system was ready for the production run, which was performed using the Langevin thermostat under the NPT ensemble, at a 2 fs time step. Long-range electrostatics were calculated using the particle mesh Ewald method.^59^

#### Analysis

Trajectory analysis was performed using the cpptraj module^61^ of AmberTools15.^62^

RMSD was computed with backbone α-carbons for proteins, and heavy atoms for ligands with respect to the first frame using the rms tool.

The vector tool was used to calculate the angle between two vectors associated with two pairs of atoms. To follow the orientation of the ligands along the simulation, we computed the angle between two arbitrarily selected vectors, one from the α-carbon (CA) of Thr115 to the CA of Phe121, residues located at MD-2 β-sheet 7, and the other from the C1 to the C3 carbons of FP glucosamine group. To follow the orientation of the MD-2 Phe126 side chain, we computed the angle between two arbitrarily selected vectors, one from the CA to the ζ-carbon (CZ) of Phe126 and the other from the CA of Phe126 to the CA of Ser33.

With the distance tool, we tracked interatomic distances. For polar interactions of FP compounds to (TLR4/MD-2)2 in type-A orientation, we computed interatomic distances between the P atom of phosphate group C4 to selected atoms of TLR4 residues (ζ-nitrogen (NZ) of Lys341, NZ of Lys362, and CZ of Arg264), and to selected atoms of MD-2 (η-oxygen (OE) of Tyr102, δ-carbon (CD) of Glu92); between the O atom of the hydroxyl C6 group to selected atoms of TLR4* residues (CD of Gln436*, CA of Ser415*) and MD-2 residues (NZ of Lys122 and CZ of Arg90); and between the C2 glucosamine atom to selected atoms of MD-2 rim residues (CA of Ser120, CA of Ser118, CA of Arg90, and CA of Glu92). For polar interactions of FP compounds to (TLR4/MD-2)2 in type-B orientation, we computed interatomic distances between the P atom of phosphate group C4 to selected atoms of TLR4 residues (NZ of Lys341, and NZ of Lys362), TLR4* residues (CD of Gln436* and CD of Glu439*), and MD-2 residues (NZ of Lys122, and CZ of Arg90), between the O atom of the hydroxyl C6 group to selected atoms of TLR4 residues (NZ of Lys341, NZ of Lys362, and CZ of Arg264), and MD-2 residues (CD of Glu92), and between the C2 glucosamine atom to selected atoms of MD-2 rim residues (CA of Ser120, CA of Ser118, CA of Arg90, and CA of Glu92).

The nativecontacts tool was used to get minimum distances between sets of atoms. For hydrophobic interactions of FA chains of FP compounds to residues within the MD-2 pocket, we computed the minimum distance from any carbon atom of FA chains 1, 2, and 3 of FP compounds to any heavy atom of side chains of Val82, Phe141, and Val113.

Molecular visualization and graphics were generated using Visual Molecular Dynamics (VMD) software,^63^ and PyMOL molecular graphics and modeling package.

### MD Simulations of α-**FP20** and β-**FP20** Self-Assembly

Initial systems were set up. Initial configurations for MD simulations were built using the freely distributed Packmol package.^64^ Two simplistic systems were created, each of them consisting of a water cubic box of (80) Å3 with 128 molecules of either α-FP20 or β-FP20 and Na+ counterions. All the molecules were randomly distributed.

#### MD Simulations

Based on our previously reported studies on the self-aggregation of other FP analogues,^26^ the systems were simulated at 350 K to minimize the simulation time while increasing the assembly speed. Within the NpT ensemble, pressure was handled both isotropically (to trigger the self-assembly). Thus, the systems were first simulated in isotropic conditions at 350 K for 200 ns. The MD protocol used was the same as the one described for the simulations of the docking poses except for the temperature value (350 K instead of 300 K).

#### Analysis

Trajectory analysis was performed using the cpptraj module^61^ of AmberTools15. Molecular visualization and graphics were generated using VMD software^63^ and PyMOL molecular graphics and modeling package.^51^

The area of the bilayer was calculated as follows: area per lipid = (box *X* dimension × box *Y* dimension). The periodic box dimensions were extracted from the trajectory using cpptraj and following the Lipid14 tutorial at the Amber16 website (https://ambermd.org/tutorials/advanced/tutorial16/).

### Cryo-TEM Sample Preparation and Acquisition

Prior to vitrification, the glycolipid **FP20** was dissolved in 100 mM phosphate buffer (pH 7.4) solution with 16% of DMSO to a final concentration of 0.65 mg/mL. 4 μL of the sample were then applied onto a 200-mesh Quantifoil R 2/2 copper grid and vitrified using a LEICA EM GP2 plunge freezer (Leica). 2D images were collected using a JEOL JEM-2200 transmission electron microscope (JEOL Japan) operating at 200 kV in cryo-conditions and equipped with a K2 Summit direct detection camera (GATAN). Different magnifications were tested ranging from 2000× to 30,000×. The images collected at high magnification (30,000×) resulted into a 0.13 nm pixel size at the specimen.

### Cell Cultures

All cell lines were cultured according to the suppliers’ instructions. HEK-Blue cells and RAW-Blue cells (InvivoGen) were cultured in Dulbecco’s modified Eagle’s medium (DMEM) high glucose supplemented with 10% heat-inactivated fetal bovine serum (FBS), 2 mM glutamine, and 100 U/mL of penicillin and streptomycin. THP-1 X-Blue cells (InvivoGen) were cultured in Roswell Park Memorial Institute Medium (RPMI) supplemented with 10% heat-inactivated FBS, 2 mM glutamine, and 100 U/mL of penicillin and streptomycin. Cells were maintained by splitting them three times a week at a density of 0.5 × 10^6^ cells/mL. For experimental procedures, HEK-Blue cells and RAW-Blue cells were plated at a density of 0.4 × 10^4^ cells per well while THP-1 X-Blue were seeded at a density of 0.4 × 10^6^ cells/mL and plated using 200 μL/well (96-well-plate), 1 mL/well (24-well-plate) 1.5 mL/well (12 well-plate), and 3 mL/well (6 well-plate). THP1 cells were differentiated into TDM by adding phorbol 12-myristate 13-acetate (PMA, InvivoGen) at a concentration of 100 ng/mL. After 72 h of incubation with PMA, cell differentiation was assessed by optical microscope inspection and the culture medium was replaced with fresh PMA-free RMPI. Cells were rested for 24 h before exposure to the tested compounds. PBMCs were collected from healthy donors, provided by the Niguarda Hospital blood bank. PBMCs were isolated through density gradient centrifugation (Lympholyte-H; Cedarlane Labs, Burlington, Ontario, Canada). Briefly, buffy coats were diluted 1:1 with phosphate-buffered saline (PBS). Lympholyte-H was used for density gradient centrifugation according to the manufacturer’s instructions. PBMCs were harvested from the interface and washed in PBS. An aliquot of the isolated cells was diluted in trypan blue 0.1% to check for viability and counted. Cells were resuspended in RPMI supplemented with 10% FBS and 100 U/mL of penicillin and streptomycin.

### Cell Stimulation and Treatments

Cells were stimulated with 100 ng/mL of ultrapure Smooth-LPS from *S. minnesota* (S-LPS, Innaxon). MPLA R595 (Re) from *S. minnesota* (Innaxon) was used in the stated concentrations. PAM2CSK4 (InvivoGen) was used as a positive control for TLR2 activation at a concentration of 10 ng/mL. **FP20**–**24**, **FP200**, and **FP20**-α were used in the abovementioned concentrations. MCC950 (Merck) was used in the following concentrations: 0.01, 0.1, 1, and 10 μM to assess NLRP3 activation.

### Cell Reporter Assay

After addition of the compounds to be tested and selected controls, cells were incubated for 18 h. Supernatants were collected and SEAP levels quantified using the QUANTI-Blue assay (InvivoGen). Briefly, 20 μL of supernatants of reporter cells, HEK-Blue, RAW-Blue, or THP-1XBlue, was incubated with 180 μL of the QUANTI-Blue substrate in a 96-well plate for 0.5–4 h at room temperature. SEAP activity, as an indicator of TLR4 or TLR2 activation, was assessed reading the well’s optical density (OD) at 630 nm. The results were normalized with positive control (Smooth-LPS for HEK-Blue hTLR4 cells or PAM2CSK4 for HEK-Blue hTLR2 cells) and expressed as the mean of percentage ± standard error of the mean (SEM) of at least three independent experiments.

### Cytokine Enzyme-Linked Immunosorbent Assay (ELISA)

TNF, IL-1β, and IL-6 levels were measured in supernatants after the indicated timing using the respective sensitive enzyme-linked immunosorbent assay (ELISA) kits (R&D Systems; #DY210-05, #DY201-05, #DY206-05, Minneapolis). The optical density of each well was determined using a microplate reader set to 450 nm (wavelength correction: 570 nm).

### Western Blot Analysis

For cell extracts, cells were washed twice in ice-cold PBS and lysed in radioimmunoprecipitation assay buffer (RIPA) buffer (CST, #9806), supplemented with protease (Roche, Mannheim, Germany) and phosphatase inhibitors (CST ##5870). After centrifugation at 13,000 RCF for 30 min at 4 °C, the supernatants were collected as whole-cell lysates. Cell lysates were resuspended in the Laemmli buffer, denatured for 5 min at 100 °C, and separated on 10% polyacrylamide gels. Proteins were transferred on poly(vinylidene difluoride) (PVDF) filters (Bio-Rad), blocked in 5% w/v BSA TTBS, and incubated with the primary and corresponding secondary antibodies indicated below. Proteins were revealed by chemiluminescence (LiteAblot EXTEND, Euroclone) and detected using Odyssey Fc LI-COR Imaging System. The PVDF membrane filters were incubated with primary anti-phospho-p38 MAPK (Thr180/Tyr182) (D3F9) and anti-β-actin (13E5) rabbit mAb (CST #4970 diluted 1:1000) followed by anti-rabbit IgG and HRP-linked secondary antibody (Cell Signaling #7074, diluted 1:2000). Densitometric analysis was carried out using Image J.

### Immunofluorescence Analysis

TDM (2 × 10^4^ cells/well) were seeded into PhenoPlate 96-well, black, optically clear flat-bottom, poly-d-lysine-coated microplates (6055500, PerkinElmer Inc., Milan, Italy), where they were exposed to PMA. After differentiation, cell culture media were replaced with either fresh RPMI (NT) or RPMI containing 100 ng/mL LPS or 25 μM FP20, respectively. At the end of the time course treatment (0–4 h), cells were fixed with paraformaldehyde 4% (F8775, Sigma-Aldrich, Inc., St. Louis, MO 63103, United States) and permeabilized with 0.5% Triton X-100 solution or fixed and permeabilized with ice-cold 100% methanol (according to Ab manufacturer’s instructions). Then, blocking was performed using 1× PBS/5% BSA/0.3% Triton X-100. Subsequently, cells were labeled with NF-κB p65 XP Rabbit mAb (1:400; 8242, Cell Signaling Technology, Inc.) or Phospho-IRF-3 (Ser386) XP Rabbit mAb (1:400; E7J8G, Cell Signaling Technology, Inc.). Cells were then tagged with PhenoVue Fluor 568 conjugated anti-rabbit secondary antibody (2GXRB568C1, PerkinElmer Inc., Milan, Italy) to allow target visualization. Nuclei were counter-labeled with PhenoVue Hoechst 33342 Nuclear Stain (CP71, PerkinElmer Inc., Milan, Italy). Images were acquired using the Operetta CLS High-Content Analysis System and analyzed by using the Harmony 4.5 software (PerkinElmer Inc., Milan, Italy).

### Murine Immunization Experiments

Animal protocols were approved by CIC bioGUNE’s Animal Research Ethics Board in accordance with Spanish and European guidelines and regulations. Thirty-eight female C57BL/6 J mice were purchased from Charles River Laboratories (Lyon, France). Upon arrival, animals were maintained under 12 h light/dark cycles while receiving food and water ad libitum and were rested for 2 weeks prior to immunization.

MPLA (InvivoGen), FP18, and FP20 were reconstituted in DMSO (Sigma-Aldrich) at a concentration of 1 mg/mL. EndoFit ovalbumin (InvivoGen) was utilized for immunizations. Inoculations were formulated OVA, adjuvant (or vehicle), and PBS to achieve the appropriate dosages. Immunizations began at approximately 9 weeks of age. At day 0, mice received subcutaneous injections of 10 ug OVA with or without 10 μg of adjuvant. Mice were boosted with identical injections at day 21. Bleeding was performed the day before the first and second immunizations. At day 42, mice were euthanized by carbon dioxide followed by cervical dislocation, and blood was collected via intracardiac puncture. Blood collected in serum separator tubes (BD) was centrifuged at 10,000 RPM for 5 min, and serum was stored at −80 °C until use.

Antibody responses were measured by capture ELISA. NUNC plates (Thermo Fisher) were coated overnight with 100 μL of coating buffer, containing 0.5 μg/mL OVA in 0.2 M sodium bicarbonate at pH 9.6. Following four washes with 200 μL of 0.05% Tween-PBS wash buffer in an automatic plate washer (BioTek), plates were blocked for 1 h with filtered 1% BSA-PBS assay diluent. Wells were aspirated, and serial dilutions (twofold) of the sera, starting at 1/100, were applied, followed by an incubation of 1 h. Horseradish peroxidase-conjugated goat anti-mouse secondary antibodies against IgG (Jackson ImmunoResearch), IgG1, IgG2b, IgG2c, and IgG3 (SouthernBiotech) were diluted 1:1000 in assay diluent. After six washes with wash buffer, 100 μL of secondary antibody was added to each well for 45 min. Plates were washed eight times with 200 μL wash buffer, and then two times with 400 μL PBS. One hundred microliters of 3,3′,5,5′-tetramethylbenzidine peroxidase substrate solution (TMB, SeraCare) was added to each well and incubated for 30 min. After stopping the reaction with 100 μL of 2 M sulfuric acid, samples were measured with a microplate reader (BioTek Epoch) at 450 nm.

### Statistical Information

All experimental results represent the mean ± standard error of the mean (SEM) of at least three independent experiments. Western blot experiments and protein amount were evaluated in relation to the housekeeping gene β-actin. The Western blots shown were representative data from at least three independent experiments. For ELISA experiments, means were compared by *t*-tests (two groups) or one-way ANOVA (three or more groups). The Tukey multiple-comparison test following one-way ANOVA was performed to obtain adjusted *P* values. For statistical comparisons of immunization results, the area under the ELISA titration curves was examined by Brown–Forsythe and Welch one-way ANOVA tests with an α of 0.05.

## Supporting Information Available

Computational studies on aggregation, TLR4/MD-2 binding, and molecular dynamics (PDF)
FP20 type-A orientation (PDB)
FP20 type-A orientation involving the MD-2 channel (PDB)
FP20 type-B orientation (PDB)
FP22 type-A orientation (PDB)
FP22 type-A orientation involving the MD-2 channel (PDB)
FP22 type-B orientation (PDB)
FP24 type-A orientation (PDB)
Biological tests in vitro and in vivo, ^1^H NMR and ^13^C NMR spectra of all compounds, qNMR, and molecular formula strings (CSV)

## Supplementary Material

jm2c01998_si_001.pdf

jm2c01998_si_002.pdb

jm2c01998_si_003.pdb

jm2c01998_si_004.pdb

jm2c01998_si_005.pdb

jm2c01998_si_006.pdb

jm2c01998_si_007.pdb

jm2c01998_si_008.pdb

## Abbreviations

AP-1: activator protein
AS: adjuvant systems
CMC: critical micelle concentration
Cryo-EM: cryogenic electron microscopy
DLS: dynamic light scattering
DMAP: *N*,*N*-dimethylaminopyridine
ELISA: enzyme-linked immuno- sorbent assay
FA: fatty acid
HPV: human papillomavirus
IFNs: interferons
IgG: immunoglobulin G
IL-: interleukin-
IRF-3: interferon regulatory factor 3
LBP: LPS-binding protein
LPS: lipopolysaccharide
LUV: large unilamellar vesicles
MAPK: mitogen-activated protein kinase
MD: molecular dynamics
MD-2: myeloid differentiation factor 2
MPLA: monophosphoryl lipid A
MyD88: myeloid differentiation factor 88
NF-κB: nuclear factor kappa-light- chain enhancer of activated B cells
NLRP3: NLR family pyrin domain containing 3
NT: non-treated
OVA: ovalbumin
PMA: phorbol 12-myristate 13-acetate
PRRs: pattern recognition receptors
RMSD: root-mean-square deviation
S-LPS: smooth lipopolysaccharide
SEAP: secreted embryonic alkaline phosphatase
SEM: standard error of the mean
SUVs: small unilamellar vesicles
TBDMS: *tert*-butyldimethylsilyl
TDM: THP-1-derived macrophages
TEA: triethylamine
THF: tetrahydrofuran
TLRs: toll-like receptors
TNF: tumor necrosis factor
TRAM: TRIF-related adaptor molecule
TRIF: TIR domain-containing adaptor inducing IFN-β
