# Supplementary material for: New Glucosamine-Based TLR4 Agonists: Design, Synthesis, Mechanism of Action, and In Vivo Activity as Vaccine Adjuvants
Source: J Med Chem. 2023 Feb 2;66(4):3010–29. doi: 10.1021/acs.jmedchem.2c01998 (PMC9969399; doi:10.1021/acs.jmedchem.2c01998)
Supplement: Supplementary file 1 — jm2c01998_si_001.pdf [file jm2c01998_si_001.pdf]

Supporting Information

New glucosamine-based TLR4 agonists: design, synthesis, mechanism of action and in vivo activity as vaccine adjuvants

Alessio Romerio,[1a] Nicole Gotri,[1a] Ana Rita Franco[1a], Valentina Artusa,[1] Mohammed Monsoor Shaik,[1] Samuel T. Pasco,[2] Unai Atxabal,[2] Alejandra Matamoros-Recio,[3] Marina Mínguez-Toral,[3] Juan Diego Zalamea,[2] Antonio Franconetti,[2] Nicola G.A. Abrescia,[2,4] Jesus Jimenez-Barbero,[2,4,5,6] Juan Anguita,[2,4] Sonsoles Martín-Santamaría,[3] Francesco Peri[1*]

[a] *first co-authors*

[1] *Department of Biotechnology and Biosciences, University of Milano-Bicocca, Piazza della Scienza, 2; 20126, Milano, Italy*

[2] *Center for Cooperative Research in Biosciences (CIC bioGUNE), Basque Research and Technology Alliance (BRTA), 48160 Derio, Bizkaia, Spain.*

[3] *Centro de Investigaciones Biológicas Margarita Salas CSIC, C/ Ramiro de Maeztu, 9. 28040-Madrid, Spain.*

[4] *Ikerbasque, Basque Foundation for Science, Plaza Euskadi 5, 48009 Bilbao, Bizkaia, Spain.*

[5] *Department of Organic Chemistry, II Faculty of Science and Technology, EHU-UPV, 48940 Leioa, Spain.*

[6] *Centro de Investigación Biomédica En Red de Enfermedades Respiratorias, 28029 Madrid, Spain.*

Corresponding Author Email: francesco.peri@unimib.it

Index

Table of Figures and Tables 2

Chemistry 6

Computational Studies 7

1. Computational studies of the aggregation of α-FP20 and β-FP20 in water 7
2. Computational studies of the TLR4 binding of β-FP20, β-FP22, and β-FP24. 9
3. Molecular dynamics simulations of $(TLR4/MD\text{-}2)_2$ in complex with β-FP20, β-FP22, and β-FP24. 12

Biological Characterization 26

In vitro Supporting Data 26

In vivo Supporting Data 27

Bibliography 28

NMR Spectra 29

Compound FP20 29

Compound FP21 30

Compound FP22 31

Compound FP23 32

Compound FP24 33

Compound α-FP20 34

Compound 2a 35

Compound 3a 36

Compound 4a 37

Compound 5a 38

Compound 6a 39

Compound 7 40

Compound 8 41

Compound 9 42

Compound 10 43

Compound FP200 44

Purity Assessments 45

Compound FP20 45

Compound FP21 46

Compound FP22 47

Compound FP23 48

Compound FP24 49

SMILES 50

Table of Figures and Tables

Figure S1. Representation of the evolution of the α-FP20- and □-FP20-water mixtures over the simulation time (200 ns). Compounds FP20 are represented in CPK colored sticks (α-FP20 in pink and □-FP20 in green), Na^+ ions in violet spheres and the water molecules in cyan lines. 17

Figure S2. Area of the α-FP20 and □-FP20 bilayers after the self-assembly process in water. 18

Figure S3. Arrangement of the α-FP20 and □-FP20 molecules in the membrane layers, after simulation time (200 ns). (A) Lateral (on the left) and top (on the right) views of the parallel and perpendicular arrangements of the FP20 compounds. (B) A close view on the positioning of the carbonyl groups of the acyl chain at the anomeric carbon (marked with arrows). Compounds FP20 are represented in CPK colored sticks (α-FP20 in pink and □-FP20 in green), Na^+ ions in violet spheres, and the water molecules participating in water bridges with FP20 compounds, are in CPK colored wires. The rest of water molecules are hidden for clarity. 19

Figure S4. Case of docking of FP20 and FP22 onto the (TLR4/MD-2)$_2$ complex in type-A orientation with interaction of C1 fatty acid (FA) chain with MD-2 channel. On the left, 3D structure of human (TLR4/MD-2)$_2$ dimer colored as in **Figure 6**. On the right, predicted binding mode of **FP20** (green sticks) and **FP22** (blue sticks) in type-A orientation and C1 FA chain inserted onto the MD-2 channel delimited by Phe126. There is a front view (top) and top view (bottom) depicted, as well as details of the interactions with residues of MD-2 (grey sticks) and TLR4* (yellow sticks). Only a few type-A poses presented this interaction with MD-2 channel, likely because in this binding pose the glucosamine moiety appears more embedded into the MD-2 pocket compared to type-A binding pose with no FA chain in the channel. 22

Table S1. Orientation of the FP ligands and interaction with the MD-2 channel, as predicted by re-docking calculations. Calculations with AutoDock 4 predicted best docking clusters of type-A and type-B binding for **FP20** and **FP22**, and of type-B and type-C binding poses for **FP24**. 22

Figure S5. MD simulations of ligands FP20, FP22, and FP24 selected binding modes corresponding to those of Figure 6. Details of the averaged interactions between ligands FP20 (green sticks), FP22 (blue sticks), and FP24 (magenta sticks) with (TLR4/MD-2)$_2$ over simulation time. For each binding mode there is a front view (top) and top view (bottom) depicted, as well as details of the interactions with residues of TLR4 (dark blue sticks), MD-2 (grey and pink sticks), and TLR4* (yellow sticks). Note that FP24 type-A orientation corresponds to input docking type-C orientation whose representative interactions were soon lost and new interactions were formed that were comparable to those of docking type-A. 24

Figure S6. MD simulations of ligands FP20 and FP22 in type-A orientation with interaction of C1 FA chain with the MD-2 channel corresponding to docking predicted binding modes of Figure S4. (A) Details of the averaged interactions between ligands FP20 (green sticks) and FP22 (blue sticks) with (TLR4/MD-2)$_2$ over simulation time. For each binding mode there is a front view (top) and top view (bottom) depicted, as well as details of the interactions with residues of TLR4 (dark blue sticks), MD-2 (grey and pink sticks), and TLR4* (yellow sticks). (B) Minimum distance computed from any C atom of FA chains 1, 2 and 3 of FP compounds to any heavy atom of side chain of MD-2 Val82 to monitor the placement of the FA chains with respect to the MD-2 channel. Plotted minimum distance throughout the simulation time are a running average with a window of 5 ns (in our case, 50 frames). Distances of less than 5Å indicate interaction with MD-2 channel. Distances greater than 5Å point to a loss of said interaction. Chain A (chA) and chain B (chB) correspond to those of the (TLR4/MD-2/FP)$_2$ complex. FP20 and FP22 remove their C1 FA chain from the MD-2 channel in the first quarter of the simulation to place it within the pocket, and their averaged interactions become similar to those FP20 and FP22 starting with type-A orientation with no interaction with the MD-2 channel (Figure S5). 25

Figure S7. RMSD of chains A and B of TLR4, MD-2 and ligands FP20, FP22 and FP24 in type-A orientation along the MD simulations. FP20_ch and FP22_ch refer to the starting docking poses were C1 FA chain is placed at the MD-2 channel. RMSD was computed with backbone alpha carbons for proteins, and heavy atoms for ligands with respect to the first frame. Plotted RMSD values are a

running average with a window of 5 ns (in our case, 50 frames). Chains A and B correspond to those of the $(TLR4/MD\text{-}2/FP)_2$ complex. Note that FP24 plots correspond to input docking type-C orientation whose representative interactions were soon lost and new interactions were formed that were comparable to those of docking type-A orientation. 26

Figure S8. RMSD of chains A and B of TLR4, MD-2 and ligands FP20, FP22 and FP24 in type-B orientation along the MD simulations. RMSD was computed with backbone alpha carbons for proteins, and heavy atoms for ligands with respect to the first frame. Plotted RMSD values are a running average with a window of 5 ns (in our case, 50 frames). Chains A and B correspond to those of the $(TLR4/MD\text{-}2/FP)_2$ complex. 27

Figure S9. Orientation of FP molecules within the MD-2 pocket along the MD simulations. (A) Representation of the angle between two arbitrarily selected vectors, one from the α-carbon (CA) of Thr115 to the CA of Phe121, residues located at MD-2 β-sheet 7, and the other from the C1 to the C3 carbons of FP glucosamine group, used to follow the orientation of the ligands along the simulation. MD-2 is represented in semi-transparent grey cartoon and FP24 in CPK coloring with carbon atoms colored in pink. (B) Computed angle throughout the simulation time for complexes starting with type-A orientation (solid lines) and starting with type-B orientation (dashed lines). Plotted values are a running average with a window of 5 ns (in our case, 50 frames). Angle between 0 and 90 degrees is characteristic of the type-B binding (agonist-like) as observed in the PDB ID 3FXI (TLR/MD-2/*E. coli* $LPS)_2$ complex; angle between 90 and 180 degrees is characteristic of the type-A binding mode (antagonist-like) as observed in the PDB ID 2E59 (MD-2/lipid-IVa) complex. Chain A and B correspond to those of the $(TLR4/MD\text{-}2/FP)_2$ complex. 28

Figure S10. Polar interactions of FP compounds to $(TLR4/MD\text{-}2)_2$ in type-A orientation along the MD simulations. From top to bottom, interactions are divided between distances of P atom of phosphate C4 group to selected atoms of TLR4 and MD-2 residues, of O atom of hydroxyl C6 group to selected atoms of TLR4* and MD-2 residues, and of ester and amide groups (represented by C2 glucosamine atom) to selected atoms of MD-2 rim residues. Chains A (chA) and B (chB) correspond to those of the $(TLR4/MD\text{-}2/FP)_2$ complex. Note that FP24 plots correspond to input docking type-C orientation whose representative interactions were soon lost and new interactions were formed that were comparable to those of docking type-A orientation. 30

Figure S11. Polar interactions of FP compounds to $(TLR4/MD\text{-}2)_2$ in type-B orientation along the MD simulations. From top to bottom, interactions are divided between distances of P atom of phosphate C4 group to selected atoms of TLR4, MD-2 and TLR4* residues, of O atom of hydroxyl C6 group to selected atoms of TLR4 and MD-2 residues, and of ester and amide groups (represented by C2 glucosamine atom) to selected atoms of MD-2 rim residues. Chains A (chA) and B (chB) correspond to those of the $(TLR4/MD\text{-}2/FP)_2$ complex. 31

Figure S12. Hydrophobic interactions of fatty acid (FA) chains of FP compounds to residues within the MD-2 pocket in type-A orientation along the MD simulations. Minimum distance computed from any C atom of FA chains 1, 2 and 3 of FP compounds to any heavy atom of side chain of MD-2 Val82, Phe151 and Val113 to monitor the placement of the FA chains within the MD-2 pocket. (A) Front view and (B) top view of the representation of some of the calculated distances in yellow dashed lines where MD-2 appears in semi-transparent grey cartoon and FP20 in CPK coloring with carbon atoms colored in green. (C) Plotted minimum distances throughout the simulation time are a running average with a window of 5 ns (in our case, 50 frames). Chain A (chA) and chain B (chB) correspond to those of the $(TLR4/MD\text{-}2/FP)_2$ complex. Note that FP24 plots correspond to input docking type-C orientation whose representative interactions were soon lost and new interactions were formed that were comparable to those of docking type-A. 33

Figure S13. Hydrophobic interactions of FA chains of FP compounds to residues within the MD-2 pocket in type-B orientation along the MD simulations. Minimum distance computed from any C atom of FA chains 1, 2 and 3 of FP compounds to any heavy atom of side chain of MD-2 Val82, Phe151 and Val113 to monitor the placement of the FA chains within the MD-2 pocket (see Fig. S13.A and .B). Plotted minimum distances throughout the simulation time are a running average with a window of 5 ns (in our case, 50 frames). Chain A (chA) and chain B (chB) correspond to those of the $(TLR4/MD\text{-}2/FP)_2$ complex. 35

Figure S14. Orientation of MD-2 Phe126 side chain along the MD simulations. (A) Representation of the angle between two arbitrarily selected vectors, one from the α-carbon (CA) to the ζ-carbon (CZ) of Phe126, and the other from the CA of Phe126 to the CA of Ser33. Agonist MD-2 from PDB ID 3FXI and antagonist MD-2 from PDB ID 2E59 are represented in semi-transparent teal and pink cartoons respectively. Computed angle throughout the simulation time for complexes at ligand type-A orientation (type-C for FP24, that turned to type-A during MD simulations) (B) and starting at ligand type-B orientation (C). Plotted values are a running average with a window of 5 ns (in our case, 50 frames). Values for the angle between 0 and 100 degrees are typical of MD-2 bound to agonist-like ligands, and values between 100 and 180 degrees are typical of MD-2 bound to antagonist-like ligands. Chain A and B correspond to those of the $(TLR4/MD\text{-}2/FP)_2$ complex. 36

Figure S15. Three-dimensional conformations of the FP20, FP22, and FP24 compounds in water. The active ligands FP20 and FP22 adopt cylindrical shape, whereas FP24 displays an inverted-cone conformation. 37

Figure S16. Cell viability (A) THP-1 derived macrophages (TDM) were treated with the shown concentrations of FP20-24 for 16-18 hours. (B) PBMCs were treated with shown concentrations of FP20-22 for 16-18 hours. In (A) and (B) cell viability was tested using MTT assay. 100% cell viability was attributed to the negative control (NT). Results are shown as mean ± SEM of at least three independent experiments. 38

Figure S17. Western blot analysis of p38 activation. Differentiated THP1-XBlueTM cells were treated with 25 μM of FP20 for 0 to 3 hours. p38 was detected by western blot and its relative amount was calculated in respect to housekeeping protein β-actin. Densitometric analysis was carried out using Image J. Data are expressed as mean ± SEM of at least three independent experiments. (Treated Vs Non- treated: *P<0.05; **P<0.01; ***P<0.001; ****P<0.0001). 38

Figure S18. Liver transaminases of C57BL/6 mice immunized with FP18 and FP20 (A) Aspartate transaminase (AST) and (B) Alanine transaminase (ALT) relative to the OVA control group in mice serum 0-, 21- and 42-days post-immunization. 39

Chemistry

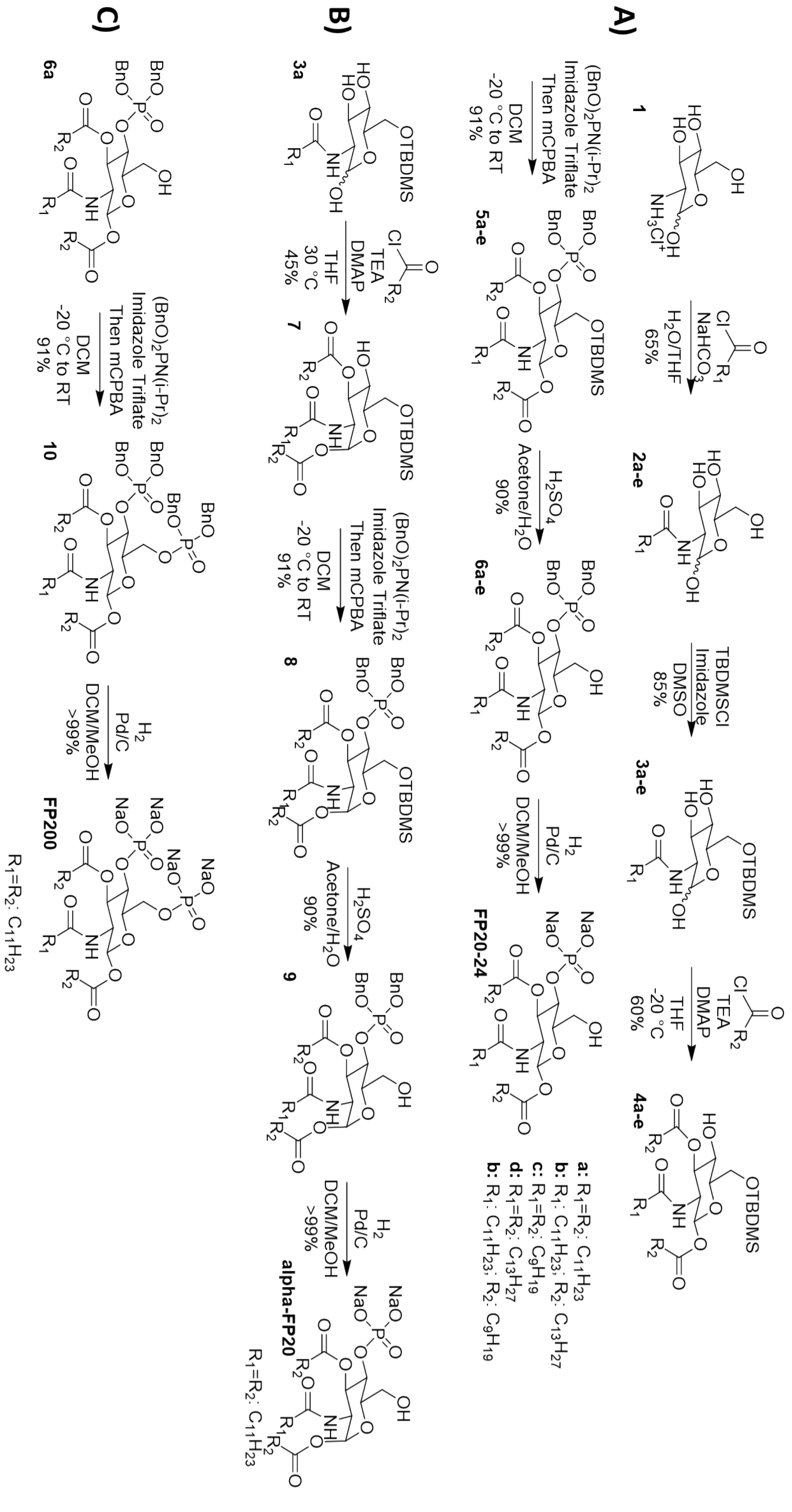

Scheme S1 (A) Synthetic pathway to compounds **FP20-24**, (B) to compound α-FP20, which can be obtained starting from intermediate **3a**, (C) to compounds **FP200**, which can be obtained starting from intermediate **6a**.

Computational Studies

1. Computational studies of the aggregation of α-FP20 and β-FP20 in water

To understand at the atomistic level the different aggregation phenomenon observed for **α-FP20** and **β-FP20,** we performed molecular dynamics (MD) simulations of each compound in water. Starting from a random mixture of either **α-FP20** or **β-FP20** molecules, Na^+ counterions, and explicit water molecules (see Computational Methods for details), the two systems were observed to self-organize into a bilayer (**Figure S1**). The bilayer area was established as the parameter to monitor the evolution of the self-aggregation and determine the equilibrated state of the systems. The corresponding area profiles decreased along the aggregation period (first 150 ns of MD simulations) and then, remained constant until the end of the simulations, pointing to a self-aggregation process by the tendency of the glycolipid molecules to protect their hydrophobic parts from water (see **Figure S2**, where the systems reached an equilibrated state after 150 ns of simulation). Importantly, the calculated area was greater for **β-FP20** compared to **α-FP20** (**Figure S2**). Since the area of the bilayer value allows the prediction of the better/poorer packing of lipid chains,[1] these results point out that the lipid chains are less packed in the case of the **β-FP20** molecules.

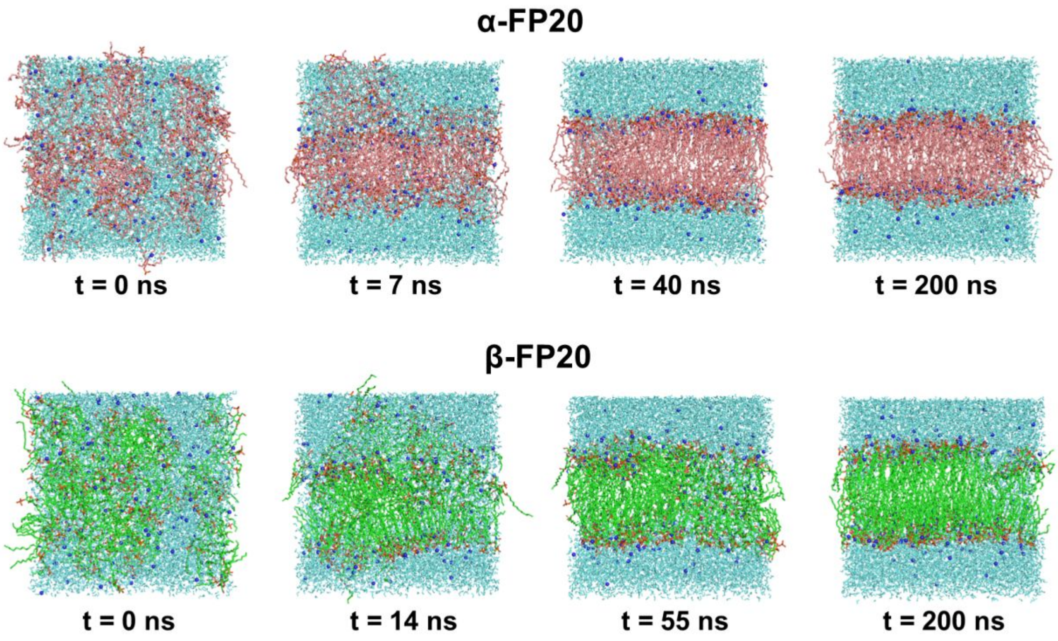

Figure S1. Representation of the evolution of the α-FP20- and β-FP20-water mixtures over the simulation time (200 ns). Compounds **FP20** are represented in CPK colored sticks (**α-FP20** in pink and **β-FP20** in green), Na^+ ions in violet spheres and the water molecules in cyan lines.

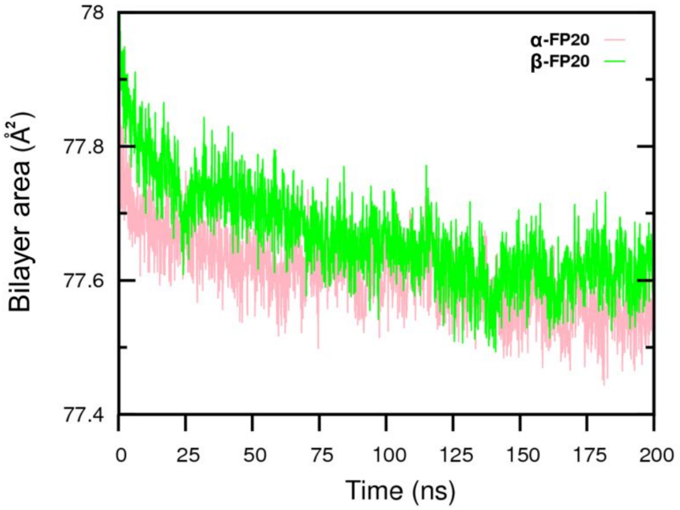

Figure S2 Area of the α-FP20 and β-FP20 bilayers after the self-assembly process in water.

We further analyzed the arrangement of the glycolipids in the bilayer and observed a common tendency for both **α-FP20** and **β-FP20** compounds: in each monolayer, **FP20** molecules were regrouped in assemblies of up to six molecules disposed with the saccharide rings packed in parallel, being stabilized by hydrogen bonds and ionic interactions (**Figure S3A**). These parallel assemblies were further arranged in perpendicular to each other, stabilized through water bridges and interactions between phosphate groups through Na^+ ionic interactions (**Figure S3A**). Although we did not find any difference regarding the assembly of the glycolipids, we observed that in the case of the **α-FP20** molecules, the carbonyl group of the acyl chain at the anomeric carbon (*i.e.,* at position 1 of the saccharide ring) was always pointing to the same face in all the molecules that formed the same arrangement (**Figure S3B**). Thus, this positioning of the carbonyl group could contribute to the ordering of the bilayer, driven by entropic factors, suggesting that the **α-FP20** compound induces a more ordered phase in the **FP20** layers, which favours more compact lipid packing.

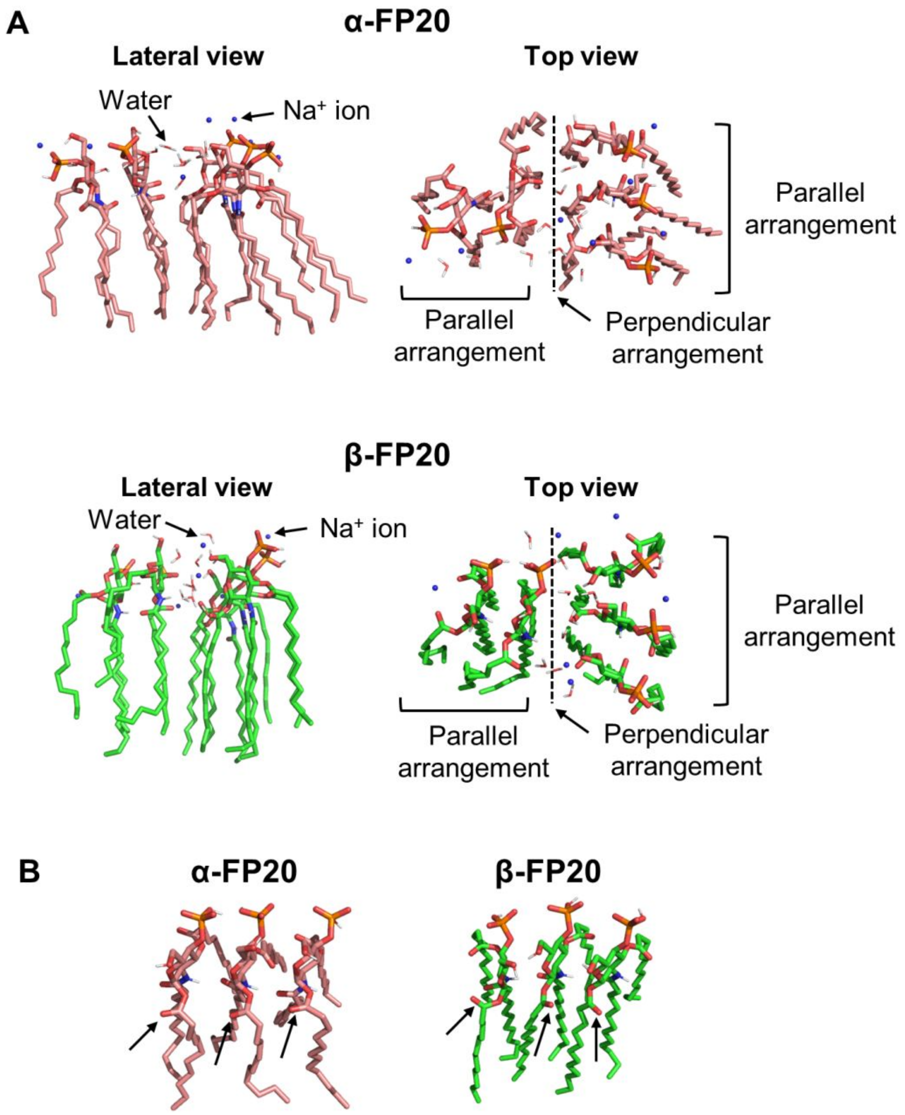

Figure S3. Arrangement of the α-FP20 and β-FP20 molecules in the membrane layers, after simulation time (200 ns). (A) Lateral (on the left) and top (on the right) views of the parallel and perpendicular arrangements of the FP20 compounds. **(B)** A close view on the positioning of the carbonyl groups of the acyl chain at the anomeric carbon (marked with arrows). Compounds FP20 are represented in CPK colored sticks (**α-FP20** in pink and **β-FP20** in green), Na^+ ions in violet spheres, and the water molecules participating in water bridges with FP20 compounds, are in CPK colored wires. The rest of water molecules are hidden for clarity.

2. Computational studies of the TLR4 binding of β-FP20, β-FP22, and β-FP24.

Molecular docking. Docking with AutoDock Vina 1.1.2[2] was used as a first approach to explore plausible binding modes, yielding poses with favourable binding energies with the fatty acid (FA) chains inserted into the hydrophobic pocket of MD-2 and the saccharide moiety positioned at the MD-2 rim. Phosphate and hydroxyl groups were found to establish polar interactions with MD-2 rim residues, especially with the side chains of Arg90, Glu92, and Tyr102, and TRL4 Arg264. The ester and amide groups of the FA chains were found to interact with MD-2 Ser120 and Lys122 backbones. For all the explored ligands, most docked poses could be classified into two main binding orientations differing by 180º rotation along the lipid chains axis: binding orientation type-A (**Figure 6**), corresponding to the lipid IVa antagonist-like orientation and the phosphate pointing towards residue Arg264 of TLR4, and binding orientation type-B (**Figure 6**), corresponding to the lipid A agonist-like

orientation in the TLR4-bound LPS, with the phosphate pointing towards residues from partner TLR4 (designated as TLR4*), and coincident with the binding modes for other reported FP compounds.[3,4]

Additionally, for **FP20** and **FP24** some docked poses were predicted to have binding orientation dubbed type-C where the saccharide moiety is shifted upward towards the partner TLR4* allowing the formation of polar interactions between the Gln436* and Glu439* side chains and the phosphate and the hydroxyl groups of the FP ligands (**Figure 6**). This type-C biding pose is similar to that for the *Escherichia coli* LPS in the X-ray crystallographic structure in complex with TLR4 (PDB ID 3FXI[5]): two FA chains bind into the MD-2 pocket and the third acyl chain is predicted to interact with the MD-2 channel, delimited by residues Leu80 and Phe126, reported to act as on-off switch.[5] According to the X-ray crystallographic structure 3FXI, this channel is occupied by one FA chain and stabilizes hydrophobic interactions with lipophilic TLR4* residues like Phe440*, Leu444*, and Phe463*.[5] Also, it was observed that this C-type binding orientation would leave space for the binding of a second FP molecule into MD-2 .

For each **FP** compound and each binding orientation, selected docked poses obtained with Vina[2] were used as starting geometries for re-docking with AutoDock 4.[6] Of the three binding modes above described, AutoDock 4 predicted best docking clusters of type-A and type-B binding for **FP20** and **FP22**, and of type-B and type-C binding poses for **FP24 (Figure 6**). Said clusters were visually inspected and compared to one another to assess the specific features of each ligand/receptor interaction.

Docked poses for **type-A binding** were very similar for **FP20** and **FP22** compounds (**Figure 6**), while for compound **FP24** type-A poses were not predicted. The phosphate group established ionic interactions and H-bonds with the TLR4 Arg264 side chain, the hydroxyl group at C6 stayed accessible to the solvent pointing towards Lys122 side chain, and the ester carbonyl of C1 with the NH from Lys122 backbone, and the NH amide group of C2 formed hydrogen bonds with the carbonyl group from Ser120 backbone, and the CO ester group of C3 established H-bond with the NH group from Ser120 backbone located at the MD-2 rim. The FA chains were inserted in a linear fashion into the MD-2 pocket, establishing van der Waals interactions with the side chain of hydrophobic residues. In particular, C1 FA chain interacted with Leu54, Phe121, Ile124, Phe126 and Ile153 side chains, C2 FA chain with Ile46, Ile52, Leu61, Phe119, Phe121 and Phe151 side chains, and C3 FA chain with Ile61, Ile63, Leu74, Phe76, Ile94 and Phe104 side chains. In the case of FP20 FA chains, composed of 12 carbons, were predicted to be more bent (case of C1 and C2 acyl chains) and inserted deeper in the pocket (case of C3 tail interacting with deeply buried Leu71) compared to those of **FP22** which are shorter (composed 10 carbons). Both **FP20** and **FP22** also presented few type-A poses with the C1 FA chain inserted in the channel delimited by Phe126 (**Table S1**), although the glucosamine moiety appeared more embedded into the MD-2 pocket (**Figure S4**).

Table S1. Orientation of the FP ligands and interaction with the MD-2 channel, as predicted by re-docking calculations. Calculations with AutoDock 4 predicted best docking clusters of type-A and type-B binding for **FP20** and **FP22**, and of type-B and type-C binding poses for **FP24**.

Binding mode	β-FP20	β-FP22	β-FP24
type-A	Few poses with the C1 FA chain in the MD-2 channel.	Few poses with the C1 FA chain in the MD-2 channel.	
type-B	50% of the docking poses placed C3 FA chain the MD-2 channel	Few poses with the C3 FA chain in the MD-2 channel.	None of the poses placed any FA in the MD-2 channel
type-C			All poses with C1 FA chain in the MD-2 channel.

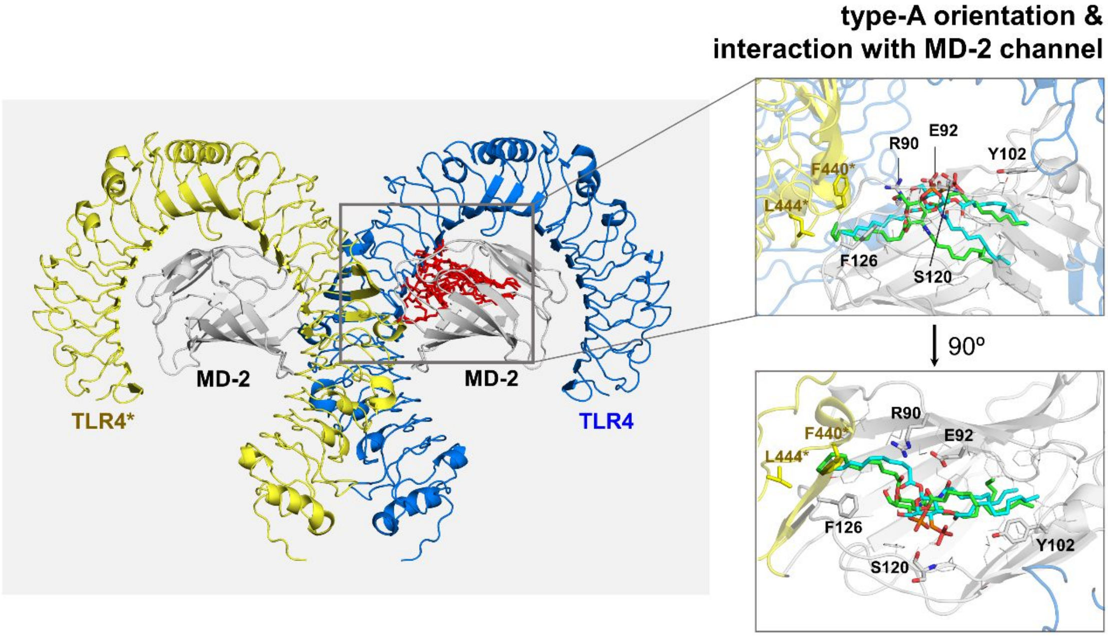

Figure S4. Case of docking of FP20 and FP22 onto the (TLR4/MD-2)$_2$ complex in type-A orientation with interaction of C1 fatty acid (FA) chain with MD-2 channel. On the left, 3D structure of human (TLR4/MD-2)$_2$ dimer (PDB ID 3FXI) colored as in **Figure 6**. On the right, predicted binding mode of **FP20** (green sticks) and **FP22** (blue sticks) in type-A orientation and C1 FA chain inserted onto the MD-2 channel delimited by Phe126. There is a front view (top) and top view (bottom) depicted, as well as details of the interactions with residues of MD-2 (grey sticks) and TLR4* (yellow sticks). Only a few type-A poses presented this interaction with MD-2 channel, likely because in this binding pose the glucosamine moiety appears more embedded into the MD-2 pocket compared to type-A binding pose with no FA chain in the channel.

Type-B binding is characterized by an 180º rotation along the FA chains axis compared to type-A binding, and was predicted for the three FP compounds **FP20**, **FP22**, and **FP24** (**Figure 6**). However, unlike type-A, type-B binding mode presented different ligand/receptor interactions for each compound. Nonetheless, the C1 and C2 FA chains were placed in the same region of the MD-2 pocket for the three **FP** molecules, where hydrophobic interactions were established among the acyl chains and the side chains of residues Tyr65, Leu71, Leu74, Phe76, Ile94, Phe104, Val113, Ile117 and Leu146 side chains for C1 FA, and residues Ile44, Ile46, Leu61, Ile63, Tyr65, Phe76, Val135 and Leu149 said chains for C2 FA. The compounds were buried deeper in the MD-2 pocket in comparison to the type-A binding. Interestingly, **FP24** acyl chains appeared more compact as C3 FA chain established van der Waals interactions near residues Ile52 and Leu61, located close to its

neighbour C2 FA chain too, while C3 FA chains of **FP20** and **FP22** were distributed more spread along the pocket.

Polar interactions in type-B binding poses were diverse (**Figure 6**). **FP20** phosphate group formed an ionic interaction with Arg90 side chain located at the MD-2 rim, and OH group at C6 established a hydrogen bond with Glu92 side chain. **FP20** ester and amide groups were embedded into the hydrophobic pocket of MD-2 and were observed later in the MD simulations to establish polar interactions (see below MD simulations section). **FP22** type-B binding pose was shifted towards TLR4 compared to **FP20** type-B binding pose, and the **FP22** phosphate group formed a hydrogen bond with Glu92 side chain. **FP22** hydroxyl group was pointing towards TLR4 Arg264 amine group, and the carbonyl group of the ester in **FP22** C1 FA chain established a hydrogen bond with the OH group of MD-2 Tyr102. **FP24** type-B binding pose displayed no specific polar interaction for the phosphate group but the hydroxyl group at C6 was in the correct orientation for potentially forming a hydrogen bond with TLR4 Arg264 side chain. The carbonyl group of C3 FA chain of **FP24** also established a hydrogen bond with the NH group from the Ser120 at the MD-2 rim.

Regarding the binding of C3 FA chain into the MD-2 channel delimited by the Phe126, in the case of **FP20**, around 50% of the docked poses presented the C3 FA chain inserted into the channel. In the case of **FP22**, only few poses were predicted with the C3 FA located at the MD-2 channel and, in the case on **FP24**, none of the binding poses presented the C3 FA chain into the channel (**Table S1**).

An LPS-like binding orientation or **type-C orientation** was predicted for compound FP24, in which hydrogen bonds were formed between the TLR4* residues Gln436* and Glu439* side chains, and **FP24** phosphate and hydroxyl group respectively (**Figure 6**). Besides, C1 FA chain was inserted in the MD-2 pocket establishing van der Waals interactions with MD-2 residues Ile80, Val82, Leu87 and Phe126, and TLR4* residues Leu444* and Phe440*. Interestingly, the chain predicted to be inserted into the MD-2 channel was C1 FA chain for **FP24** in type-C binding pose which coincides with that of type-A orientation for **FP20** and **FP22** (**Table S1** and **Figure S4**). **FP24** C2 and C3 FA chains in this type-C binding pose established hydrophobic interactions with amino acids Val24, Ile32, Val48, Ile52, Leu54, Phe119, Phe121, Phe151 and Ile153, located lining one half of the MD-2 pocket. Overall, the docking predictions point to a different behaviour for **FP24** in comparison to **FP20** and **FP22**.

3. Molecular dynamics simulations of $(TLR4/MD\text{-}2)_2$ in complex with β-FP20, β-FP22, and β-FP24.

Best poses from the docking calculations were selected to perform MD simulations, and to assess the stability of the docked complexes. These selected complexes were eight in total: two type-A poses for **FP20** and **FP22**, one with the three FA chains inserted into MD-2 pocket (**Figure 6**) and one with the C1 FA chain placed at the MD-2 channel (**Figure S4**); one type-B pose for **FP20**, **FP22**,

and **FP24**; and one type-C pose for **FP24**. Starting from the docked TLR4/MD-2/FP complexes, we constructed the eight (TLR/MD-2/FP)$_2$ models which were submitted to 200 ns MD simulations (**Figure S5** and **Figure S6**). The root-mean-square deviation (RMSD) was monitored along the simulation time and confirmed the stability of the molecules within the systems (**Figure S7** and **Figure S8**).

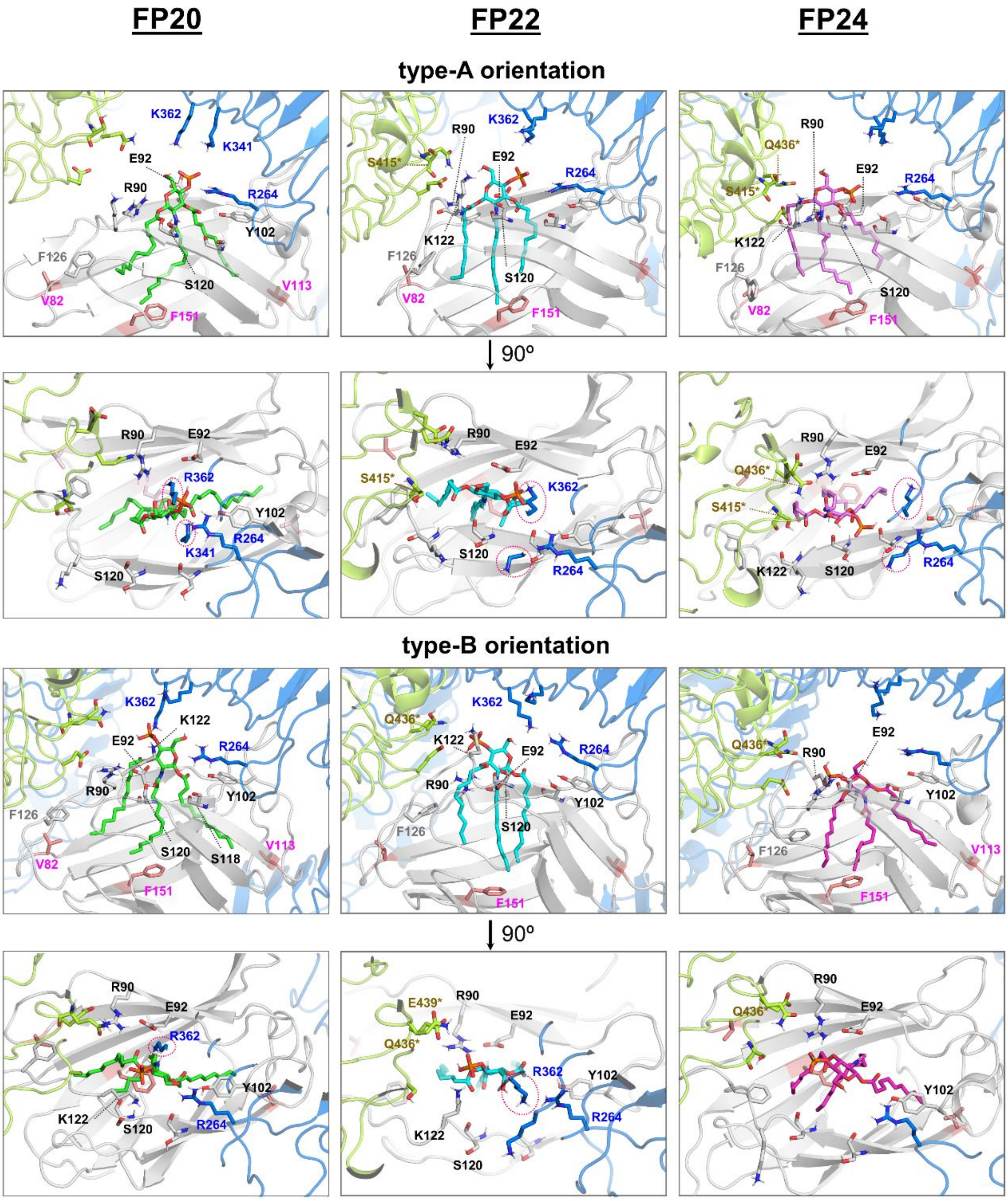

Figure S5. MD simulations of ligands FP20, FP22, and FP24 selected binding modes corresponding to those of Figure 6. Details of the averaged interactions between ligands FP20 (green sticks), FP22 (blue sticks), and FP24 (magenta sticks) with (TLR4/MD-2)$_2$ (PDB ID 3FXI) over simulation time . For each binding mode there is a front view (top) and top view (bottom) depicted, as well as details of the interactions with residues of TLR4 (dark blue sticks), MD-2 (grey and pink sticks), and TLR4* (yellow sticks). Note that FP24 type-A orientation corresponds to input docking type-C orientation whose representative interactions were soon lost and new interactions were formed that were comparable to those of docking type-A.

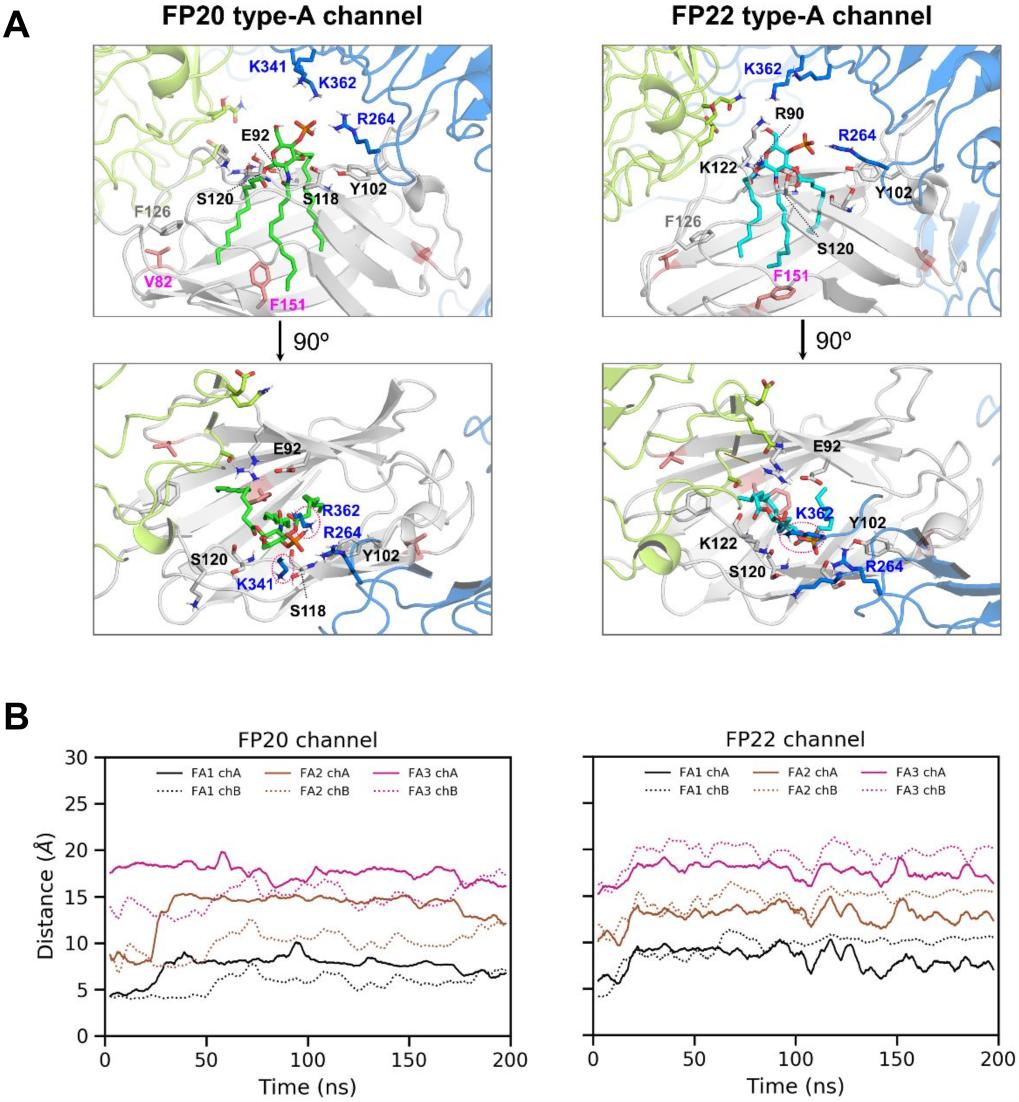

Figure S6. MD simulations of ligands FP20 and FP22 in type-A orientation with interaction of C1 FA chain with the MD-2 channel corresponding to docking predicted binding modes of Figure S4. (A) Details of the averaged interactions between ligands **FP20** (green sticks) and **FP22** (blue sticks) with $(TLR4/MD\text{-}2)_2$ (PDB ID 3FXI) over simulation time. For each binding mode there is a front view (top) and top view (bottom) depicted, as well as details of the interactions with residues of TLR4 (dark blue sticks), MD-2 (grey and pink sticks), and TLR4* (yellow sticks). **(B)** Minimum distance computed from any C atom of FA chains 1, 2 and 3 of FP compounds to any heavy atom of side chain of MD-2 Val82 to monitor the placement of the FA chains with respect to the MD-2 channel. Plotted minimum distance throughout the simulation time are a running average with a window of 5 ns (in our case, 50 frames). Distances of less than 5Å indicate interaction with MD-2 channel. Distances greater than 5Å point to a loss of said interaction. Chain A (chA) and chain B (chB) correspond to those of the $(TLR4/MD\text{-}2/FP)_2$ complex. **FP20** and **FP22** remove their C1 FA chain from the MD-2 channel in the first quarter of the simulation to place it within the pocket, and their averaged interactions become similar to those **FP20** and **FP22** starting with type-A orientation with no interaction with the MD-2 channel (**Figure S5**).

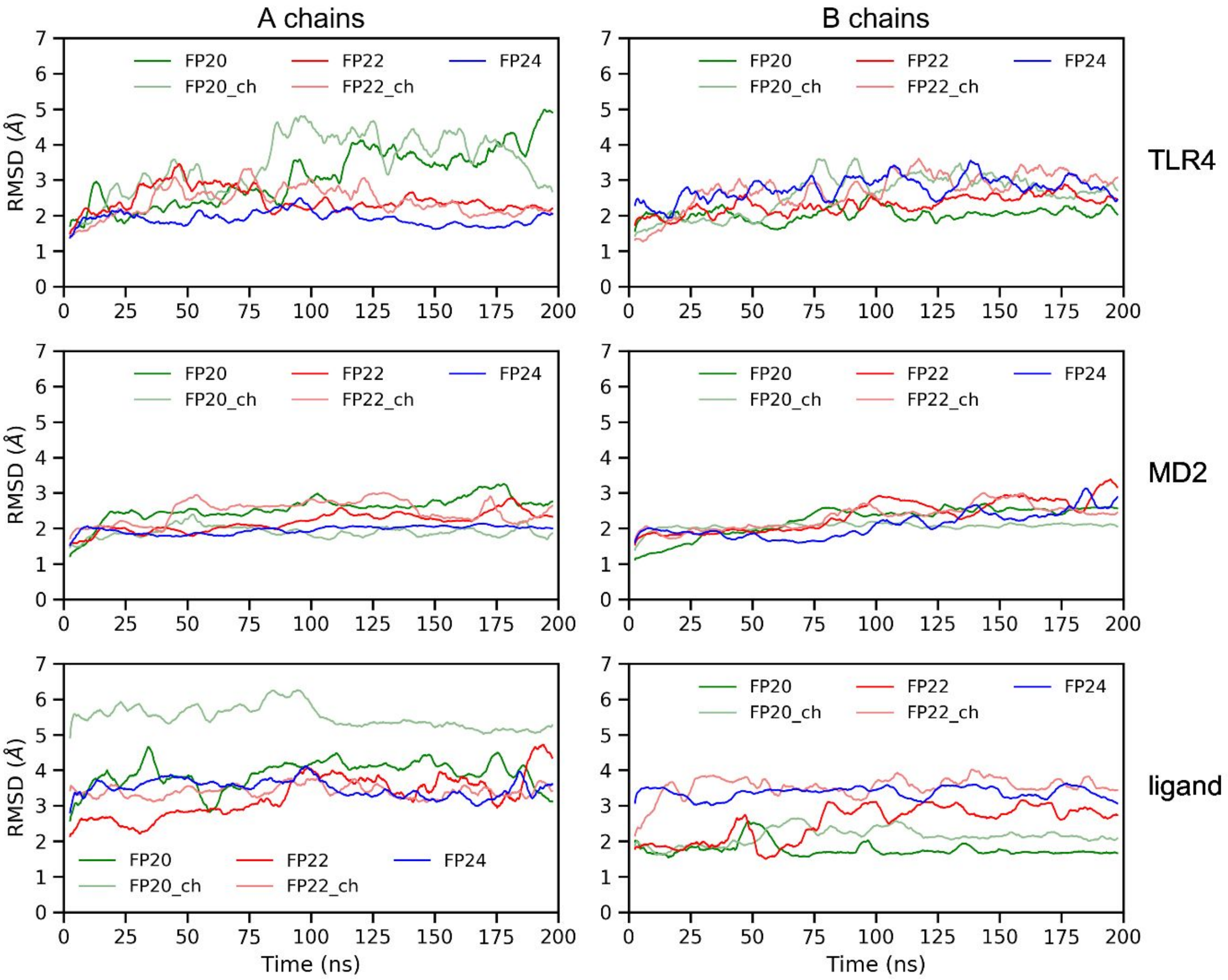

Figure S7. RMSD of chains A and B of TLR4, MD-2 and ligands FP20, FP22 and FP24 in type-A orientation along the MD simulations. FP20_ch and FP22_ch refer to the starting docking poses were C1 FA chain is placed at the MD-2 channel. RMSD was computed with backbone alpha carbons for proteins, and heavy atoms for ligands with respect to the first frame. Plotted RMSD values are a running average with a window of 5 ns (in our case, 50 frames). Chains A and B correspond to those of the $(TLR4/MD\text{-}2/FP)_2$ complex. Note that **FP24** plots correspond to input docking type-C orientation whose representative interactions were soon lost and new interactions were formed that were comparable to those of docking type-A orientation.

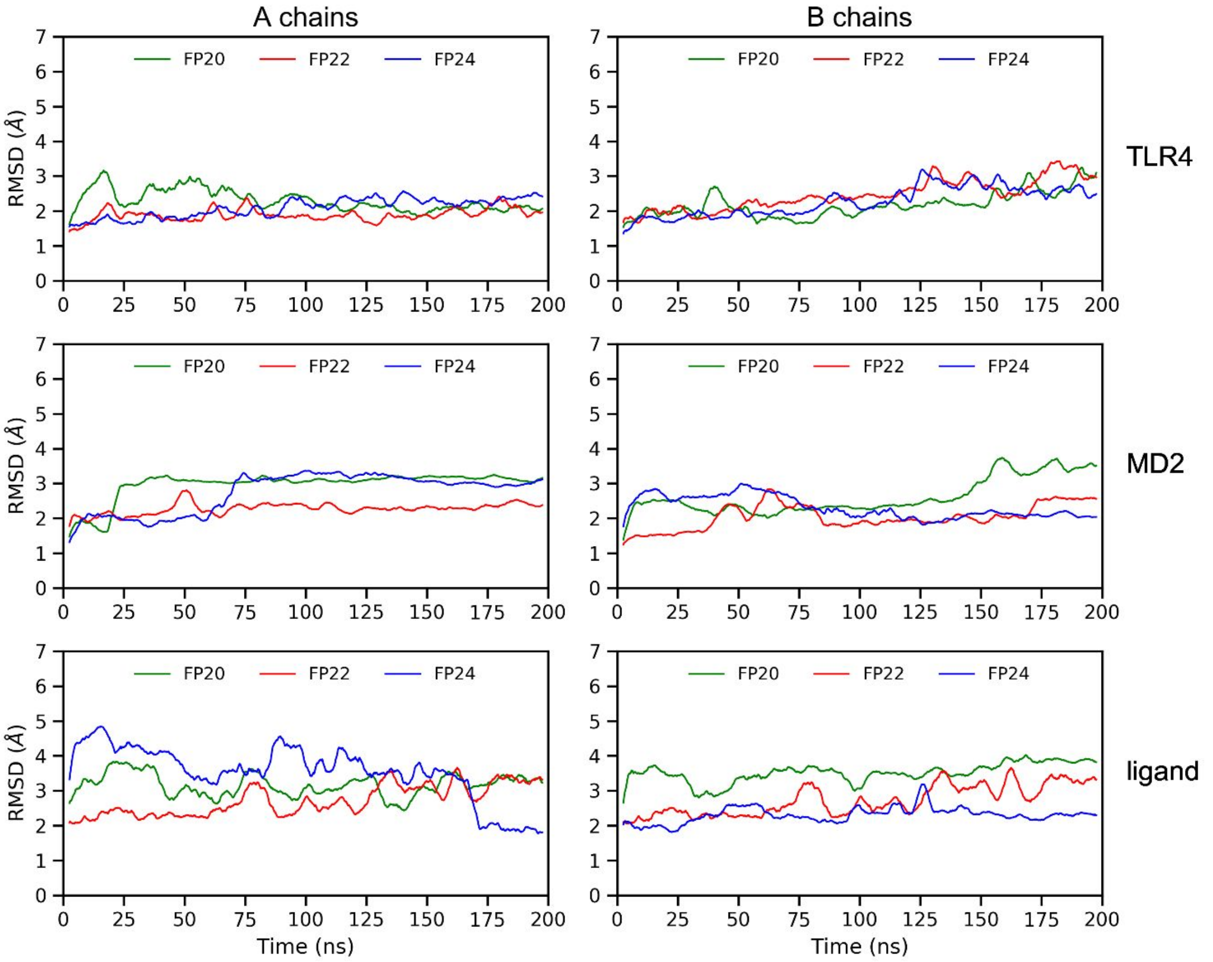

Figure S8. RMSD of chains A and B of TLR4, MD-2 and ligands FP20, FP22 and FP24 in type-B orientation along the MD simulations. RMSD was computed with backbone alpha carbons for proteins, and heavy atoms for ligands with respect to the first frame. Plotted RMSD values are a running average with a window of 5 ns (in our case, 50 frames). Chains A and B correspond to those of the $(TLR4/MD\text{-}2/FP)_2$ complex.

Further analyses were then performed. For FP20 and FP22 starting in type-A orientation with C1 FA inserted onto the MD-2 channel, the interaction with the channel was not retained and the final structures after the MD simulations became similar to those of FP20 and FP22 starting with type-A orientation with no interaction with the MD-2 channel (**Figure S6**). Henceforth we will only address simulations of type-A poses for FP20 and FP22 starting with the three FA chains inserted into the MD-2 pocket. The orientation of the FP molecules along the simulation was assessed, observing that the FP compounds did not undergo orientation flip, *i.e.*, that the ligands did not change from type-A orientation to type-B or vice versa (**Figure S9**). This result suggests that both types of binding may be possible.

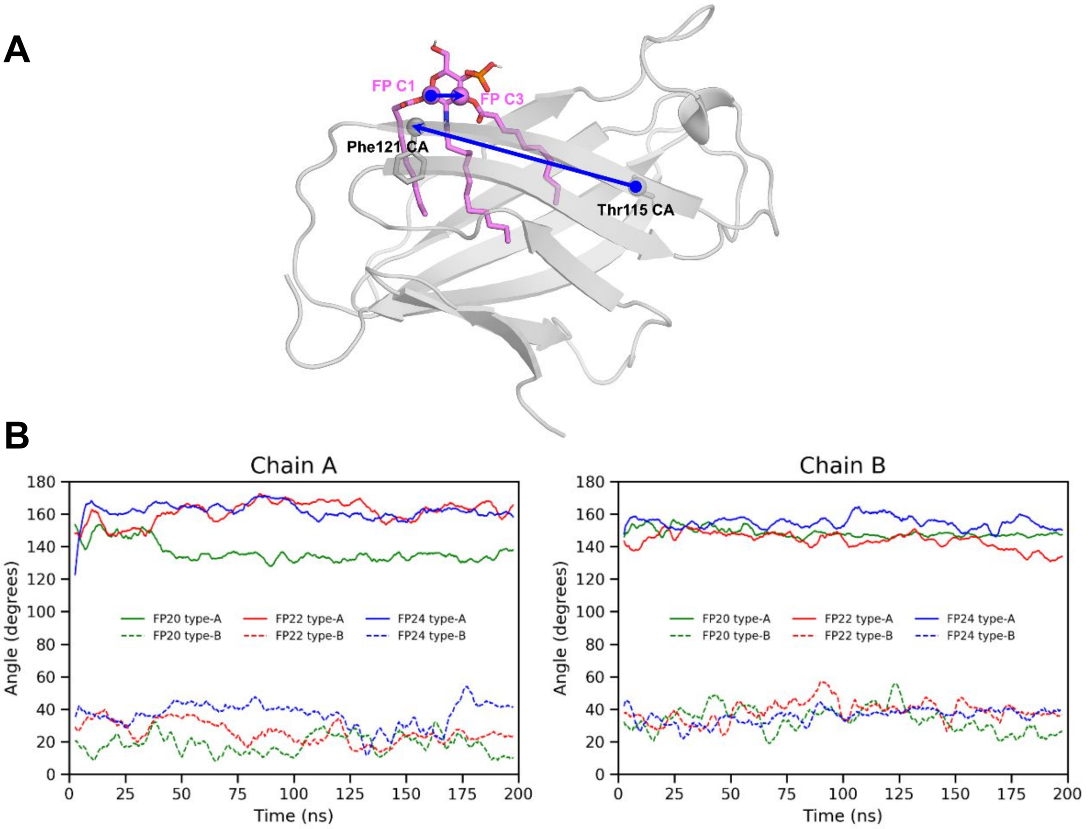

Figure S9. Orientation of FP molecules within the MD-2 pocket along the MD simulations. (A) Representation of the angle between two arbitrarily selected vectors, one from the α-carbon (CA) of Thr115 to the CA of Phe121, residues located at MD-2 β-sheet 7, and the other from the C1 to the C3 carbons of FP glucosamine group, used to follow the orientation of the ligands along the simulation. MD-2 is represented in semi-transparent grey cartoon and **FP24** in CPK coloring with carbon atoms colored in pink. **(B)** Computed angle throughout the simulation time for complexes starting with type-A orientation (solid lines) and starting with type-B orientation (dashed lines). Plotted values are a running average with a window of 5 ns (in our case, 50 frames). Angle between 0 and 90 degrees is characteristic of the type-B binding (agonist-like) as observed in the PDB ID 3FXI (TLR/MD-2/*E. coli* LPS)$_2$ complex; angle between 90 and 180 degrees is characteristic of the type-A binding mode (antagonist-like) as observed in the PDB ID 2E59 (MD-2/lipid-IVa) complex. Chain A and B correspond to those of the (TLR4/MD-2/FP)$_2$ complex.

The interactions identified for the docked complexes were monitored along the simulation time (**Figure S10** and **Figure S11**). Overall, most interactions were maintained for **FP20** and **FP22** poses with types A and B orientations and, additionally, new interactions with TLR4 Lys362 were formed. Specifically, in complexes from type-A orientation, the phosphate group established stable ionic and hydrogen bond interactions with the ammonium group of Lys362. Additionally, **FP20** phosphate group also establishes transient interactions with Lys341 side chain. Reversely, in complexes with type-B orientation, it is the hydroxyl group of the **FP** compounds the one that establishes hydrogen bonds with the Lys362 ammonium group. And again, only **FP20** showed an extra interaction between the phosphate group and the ammonium from the Lys362 side chain. However, the (TLR4/MD-2/**FP24**)$_2$ complexes behaved differently during the MD simulations, since the important interactions with either TLR4 Lys341 or Lys362 were lost on any orientation from 100 ns of the simulation (**Figure S10** and **Figure S11**). Another significant feature regarding **FP24** was discovered only through MD simulations. Type-C orientation, predicted by docking, contains hydrogen bonds between the phosphate and the hydroxyl group of FP24 and the TLR4* Gln436* and Glu439* side chains, respectively. However, these interactions were lost at the beginning of the MD simulation (at 5 ns), and **FP24** binding mode type-C evolved to type-A where the phosphate group is pointing towards

Arg264 (Figure S5). Hereafter, in the simulation analysis, we will address **FP24** type-C orientation as type-A.

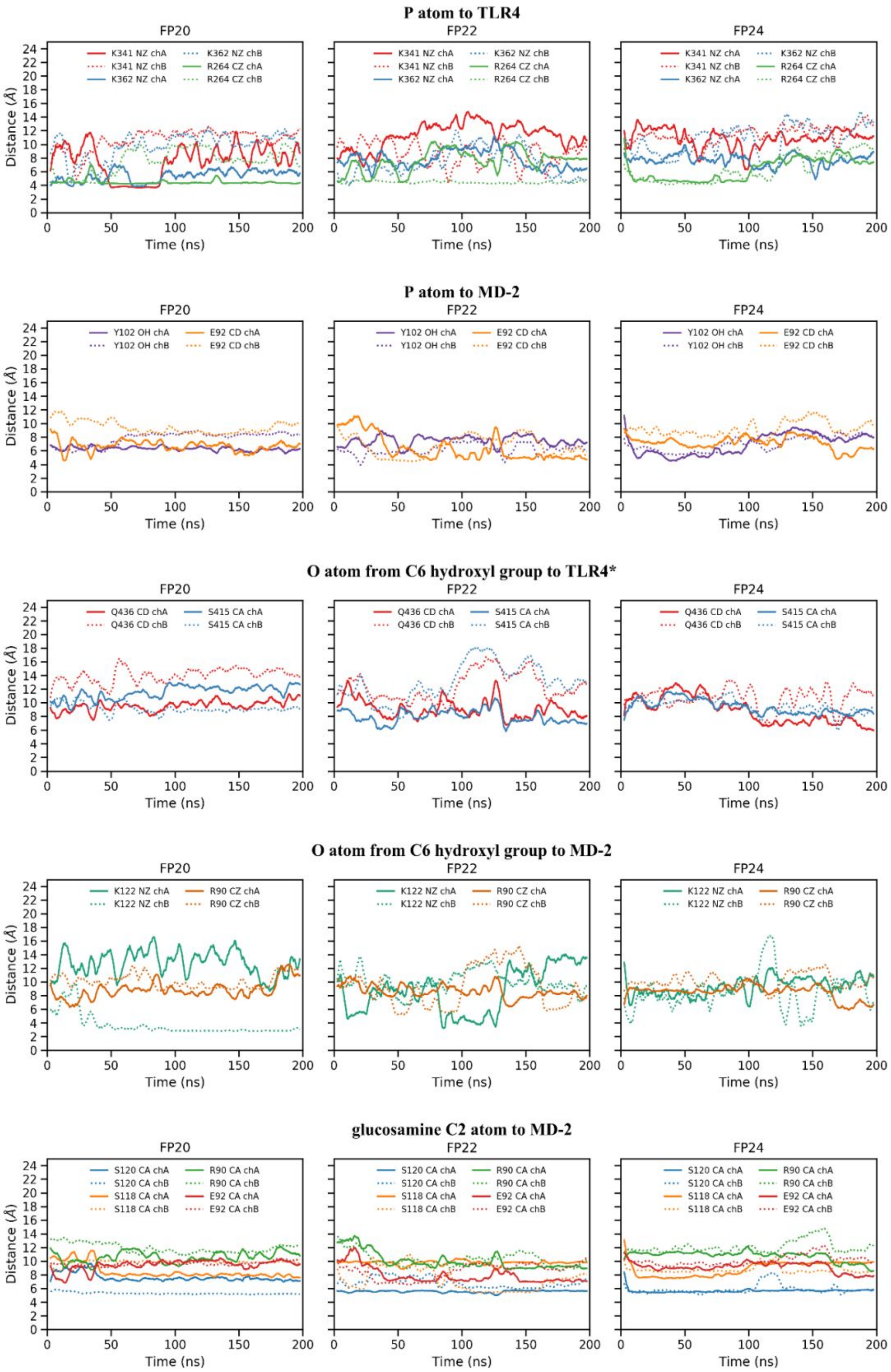

Figure S10. Polar interactions of FP compounds to $(TLR4/MD\text{-}2)_2$ in type-A orientation along the MD simulations. From top to bottom, interactions are divided between distances of P atom of phosphate C4 group to selected atoms of TLR4 and MD-2 residues, of O atom of hydroxyl C6 group to selected atoms of TLR4* and MD-2 residues, and of ester and amide groups (represented by C2 glucosamine atom) to selected atoms of MD-2 rim residues. Chains A (chA) and B (chB) correspond to those of the $(TLR4/MD\text{-}2/FP)_2$ complex. Note that **FP24** plots correspond to input docking type-C orientation whose representative interactions were soon lost and new interactions were formed that were comparable to those of docking type-A orientation.

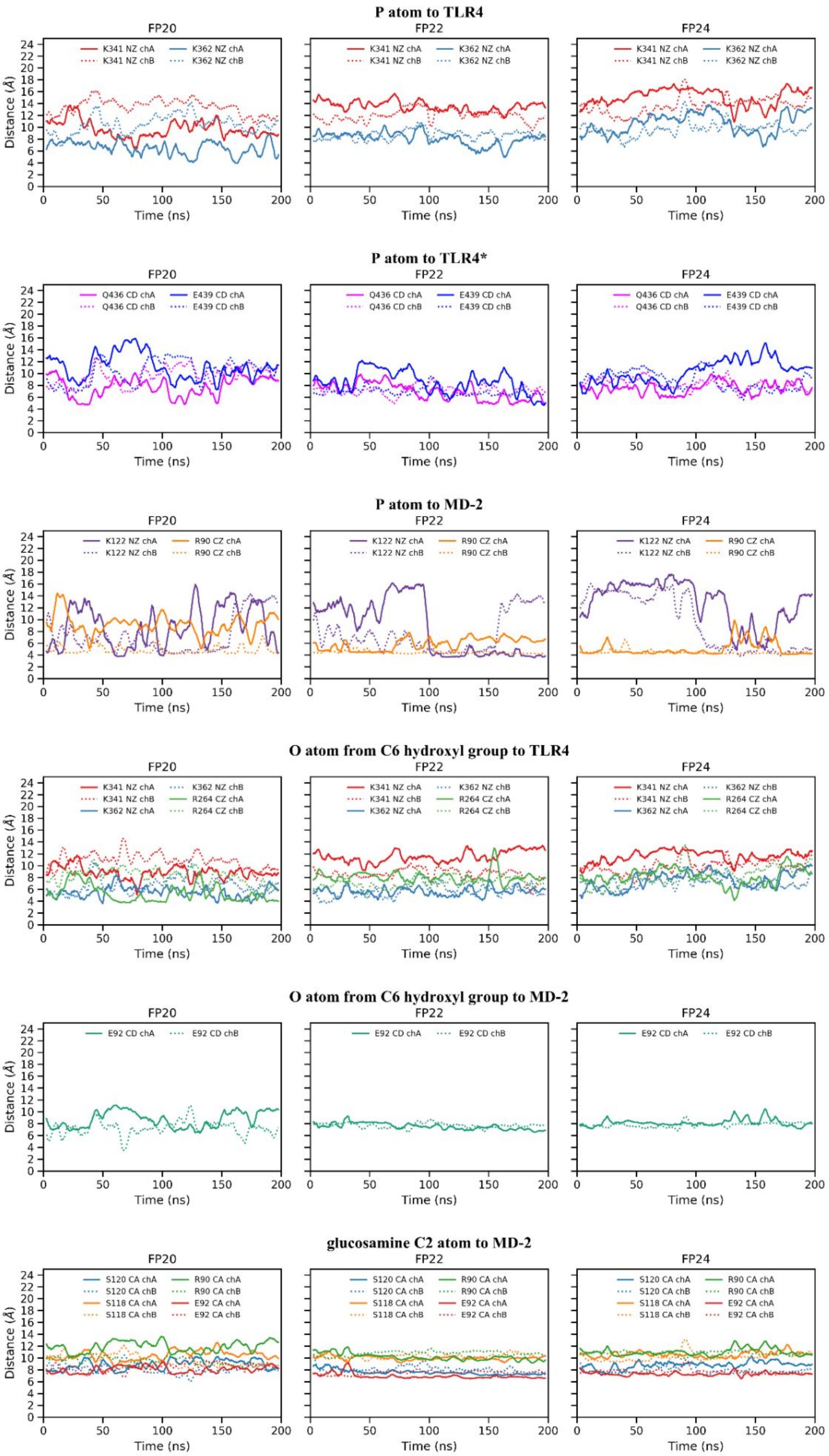

Figure S11. Polar interactions of FP compounds to $(TLR4/MD\text{-}2)_2$ in type-B orientation along the MD simulations. From top to bottom, interactions are divided between distances of P atom of phosphate C4 group to selected atoms of TLR4, MD-2 and TLR4* residues, of O atom of hydroxyl C6 group to selected atoms of TLR4 and MD-2 residues, and of ester and amide groups (represented by C2 glucosamine atom) to selected atoms of MD-2 rim residues. Chains A (chA) and B (chB) correspond to those of the $(TLR4/MD\text{-}2/FP)_2$ complex.

In detail, regarding polar interactions, in the type-A orientation (**Figure S5** and **Figure S10**), the predicted ionic interaction between the phosphate and the guanidinium group of TLR4 Arg264 was firmly formed and stabilized for **FP20** and **FP22** although it became less stable for **FP24** from 100 ns on. As mentioned above, the **FP20** OH group interacted with Lys362 side chain while hydroxyl groups of **FP22** and **FP24** were in closer contact with TRL4* Gln436 side chain instead. The sugar oxygen and the carbonyl group of FA chain C1 were in close contact with the Arg90 guanidinium group at the MD-2 rim (**Figure S5**). Another polar interaction involved a hydrogen bond between the carbonyl group of FA chain C3 and the hydroxyl group of Tyr102 for FP20. Although this interaction was lacking for **FP22** and **FP24**, these ligands established a hydrogen bonding network between carbonyl at FA chain C1, the amide at FA chain C2, and the carbonyl of FA chain C3, with the backbone of Lys122 and Ser120, as predicted by AutoDock. These interactions were also transiently formed for **FP20**.

Regarding the apolar interactions in type-A orientation, **FP** compounds were found located closer to the MD-2 rim side at β-sheet 7 comprising residues from Ile117 to Lys122. FA chains remained within the MD-2 pocket placed in a linear fashion (**Figure S5**). Only in **FP20** binding, FA chain C3 interacted with the farther side of the pocket lined by deeply buried Val133 (**Figure S5** and **Figure S12**). **FP22** and **FP24** FA chains were less spread out and did not make contact with Val113. There were no insertions of FA chains in the MD-2 channel. All in all, **FP20** compound ended up located closer to TLR4 compared to **FP22** and **FP24**.

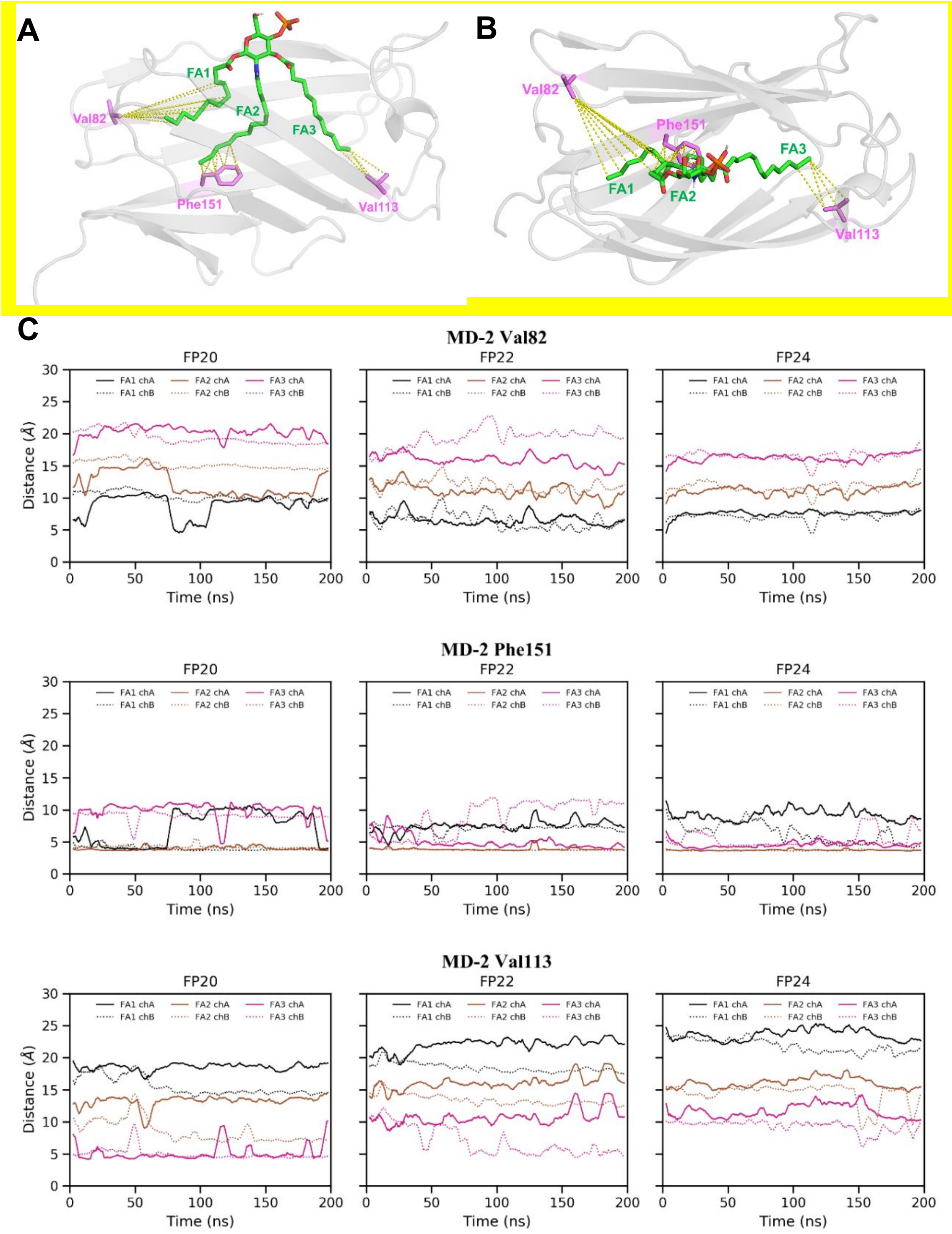

Figure S12. Hydrophobic interactions of fatty acid (FA) chains of FP compounds to residues within the MD-2 pocket in type-A orientation along the MD simulations. Minimum distance computed from any C atom of FA chains 1, 2 and 3 of FP compounds to any heavy atom of side chain of MD-2 Val82, Phe151 and Val113 to monitor the placement of the FA chains within the MD-2 pocket. **(A)** Front view and **(B)** top view of the representation of some of the calculated distances in yellow dashed lines where MD-2 appears in semi-transparent grey cartoon and FP20 in CPK coloring with carbon atoms colored in green. **(C)** Plotted minimum distances throughout the simulation time are a running average with a window of 5 ns (in our case, 50 frames). Chain A (chA) and chain B (chB) correspond to those of the $(TLR4/MD\text{-}2/FP)_2$ complex. Note that FP24 plots correspond to input docking type-C orientation whose representative interactions were soon lost and new interactions were formed that were comparable to those of docking type-A.

In detail, regarding polar interactions, in type-B orientation (**Figure S5** and **Figure S11**), **FP20** and **FP22** compounds were shifted upwards becoming less buried in the MD-2 pocket compared to the corresponding predicted binding modes of AutoDock. Meanwhile, **FP24** remained more enclosed within the MD-2 pocket (**Figure S5**). Overall, it was found again that **FP20** is more tightly anchored

to TLR4 compared to **FP22** and **FP24** (see below for detailed interactions). Hydroxyl group of **FP20** and **FP22** formed stable hydrogen bonds with guanidinium group of Arg264 as predicted by AutoDock for **FP22**, and with the side chain of the above mentioned TLR4 Lys362. These interactions were not observed for **FP24** binding. The phosphate group of **FP20** made ionic interactions reinforced by hydrogen bonds with TLR4 Lys362 and Lys341 side chains, and MD-2 Lys122 side chain. In the case of **FP22**, the phosphate group interacted with side chain of Gln436* of TLR4* MD-2 Lys122 and Arg90. As for **FP24**, its phosphate group was found to establish ionic interactions with Arg90 at the MD-2 rim. Regarding the FA chains interactions in type-B orientation, as predicted by docking, the carbonyl group of FA chain C1 formed a hydrogen bond with MD-2 Tyr102 for all the compounds. Also, for all the compounds, carboxylate of FA chain C3 and amide of FA chain C2 were forming hydrogen bonds with side chains of Arg90 and Glu92. In orientation B, **FP** compounds were found located in the middle of the MD-2 rim instead of to one side as occurs for orientation A.

As in type-A orientation, in type-B orientation no FA chain was found located at the MD-2 channel (**Figure S5**). In this case, both **FP20** and **FP24** placed their FA chain C1 in the farther side of the pocket lined by Val113 (**Figure S5** and **Figure S13**). Also, FA chain C3 of **FP20** was closer to Val82, which is neighbours to Phe126, compared to the same FA chain of FP24, meaning that FP20 was more spread out within the pocket and its FA chains were inserted in a linear fashion. **FP22** FA chains were also inserted linearly but could not come as deep. Meanwhile **FP24** FA chains were often bent, especially FA chain C2, the longest of the three FA chains of **FP24**. It indicates that the length and shape of the FA chains confer important properties to the FP compounds that affect its placing into the pocket and, in the case of **FP24**, hinder many potential polar interactions that occur for **FP20** and **FP22**.

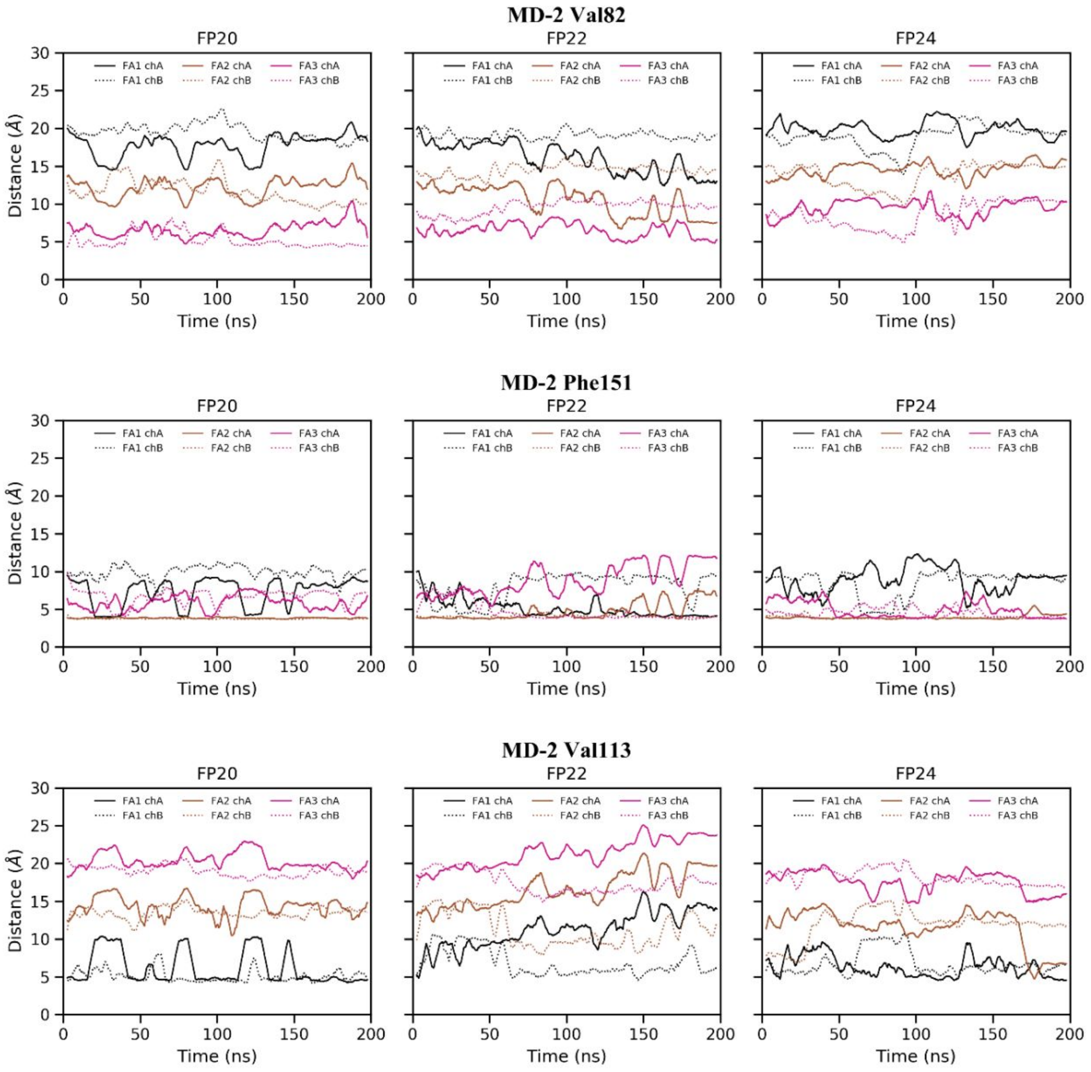

Figure S13. Hydrophobic interactions of FA chains of FP compounds to residues within the MD-2 pocket in type-B orientation along the MD simulations. Minimum distance computed from any C atom of FA chains 1, 2 and 3 of FP compounds to any heavy atom of side chain of MD-2 Val82, Phe151 and Val113 to monitor the placement of the FA chains within the MD-2 pocket (see Fig. S13.A and .B). Plotted minimum distances throughout the simulation time are a running average with a window of 5 ns (in our case, 50 frames). Chain A (chA) and chain B (chB) correspond to those of the $(TLR4/MD\text{-}2/FP)_2$ complex.

We also monitored the conformation of the Phe126 of MD-2, the on-off switch, and confirmed that retained the agonist conformation throughout the simulations for the three ligands in both orientations (**Figure S14A**). **FP20** and **FP22** retained the Phe126 agonist conformation in both MD-2 chains of the $(TLR4/MD\text{-}2/ligand)_2$ complex only in the type-B binding mode, whereas **FP24** only in type-A (type-C at the beginning of the simulations) (**Figure S14B** and **C**). Again, this indicates a different behavior of **FP24** with respect to **FP20** and **FP22**, but also that $(TLR4/MD\text{-}2/ligand)_2$ complex consistently retains the agonist conformation only for **FP20** and **FP22** when they bind in agonist-like conformation (type-A binding mode).

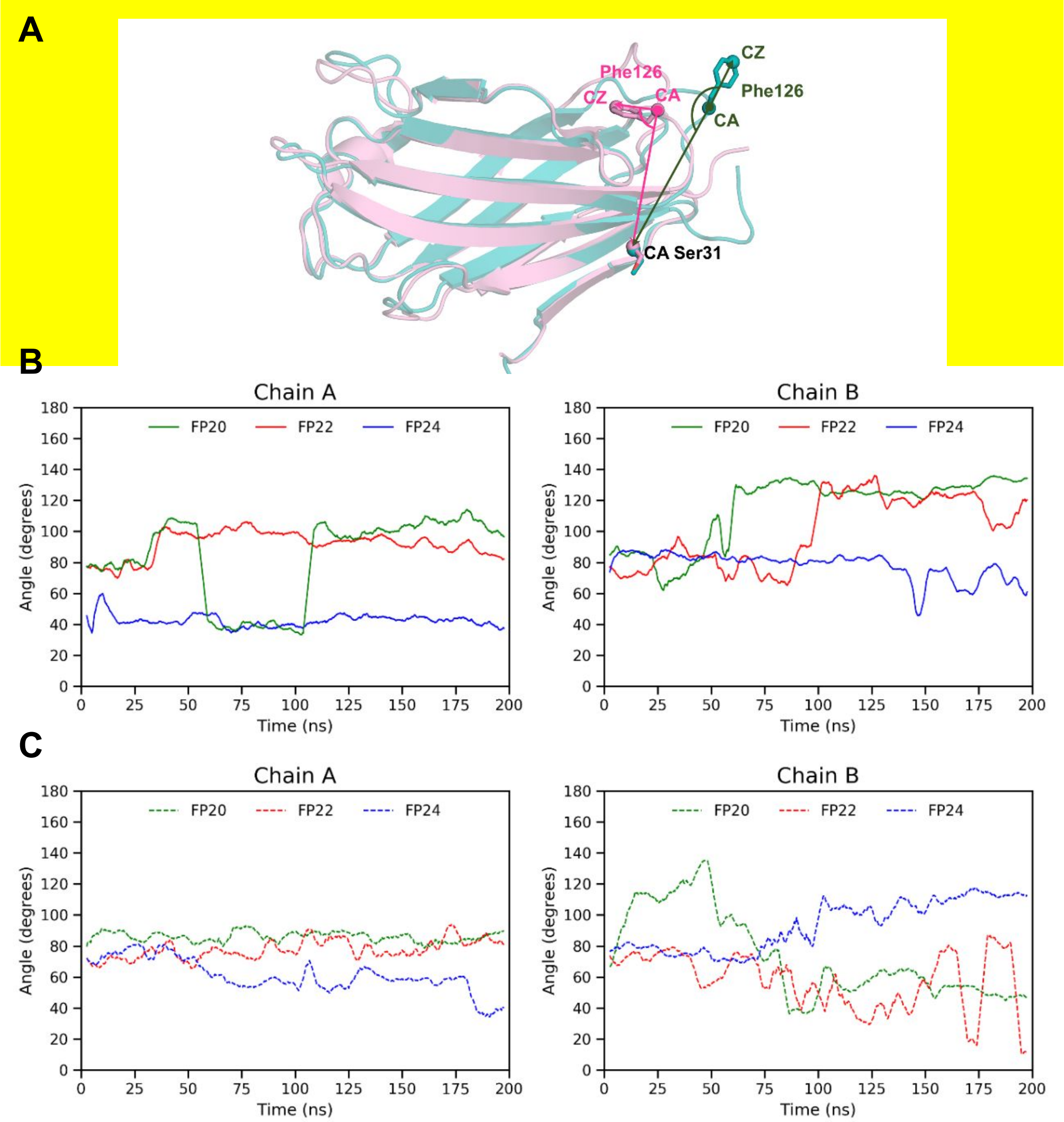

Figure S14. Orientation of MD-2 Phe126 side chain along the MD simulations. (A) Representation of the angle between two arbitrarily selected vectors, one from the α-carbon (CA) to the ζ-carbon (CZ) of Phe126, and the other from the CA of Phe126 to the CA of Ser33. Agonist MD-2 from PDB ID 3FXI and antagonist MD-2 from PDB ID 2E59 are represented in semi-transparent teal and pink cartoons respectively. Computed angle throughout the simulation time for complexes at ligand type-A orientation (type-C for FP24, that turned to type-A during MD simulations) **(B)** and starting at ligand type-B orientation **(C)**. Plotted values are a running average with a window of 5 ns (in our case, 50 frames). Values for the angle between 0 and 100 degrees are typical of MD-2 bound to agonist-like ligands, and values between 100 and 180 degrees are typical of MD-2 bound to antagonist-like ligands. Chain A and B correspond to those of the (TLR4/MD-2/FP)$_2$ complex.

Shape of glycolipids FP20, FP22, and FP24. As observed from the docking calculations and the MD simulations, FP24 behaved differently than FP20 and FP22, in agreement with the fact that FP24 is not active in TLR4. However, from the simple look to the chemical structure it is not evident the structure-activity relationship. When superposing the docked poses of these three FP compounds, it was observed that FP24 was reaching similar regions of the MD-2 pocket as FP20, due to the ability of the FA chain at C2 to reach for the deeper regions of MD-2. The presence of the long FA chain at the central position of the glycolipid made us wonder about the shape of these three FP analogues. Interestingly, it has been proposed that the shape of the lipid A component may be a key determinant of the TLR4 activation by the corresponding LPS. In general terms, it has been proposed

that lipids A that adopt a conical conformation are more active than lipids A showing a cylindrical shape.[7,8] This shape-activity relationship prompted us to study the shapes of the FP20, FP22, and FP24 compounds. To that end, we performed 100 ns of MD simulations in water of each compound and analyzed the molecular shape over simulation time. The active compounds **FP20** and **FP22** adopted a cylindrical shape, whereas the non-active **FP24** displayed an inverted-cone shape which has not been reported to date (**Figure S13**). Although the cylindrical shape of lipid A has previously been associated with a poor TLR4 activation, we propose here, based on our computational studies, that in the case of monosaccharide-based and hypo-acylated (*i.e.,* three acyl chains rather than four or more) glycolipids, the cylindrical shape may be associated with a higher activation capacity than the inverted-cone shape. Therefore, we propose that the shape of the studied FP compounds, in addition to other factors, such as the presence of a single phosphate group and its positioning at the pyranose ring,[4] influences their biological activity.

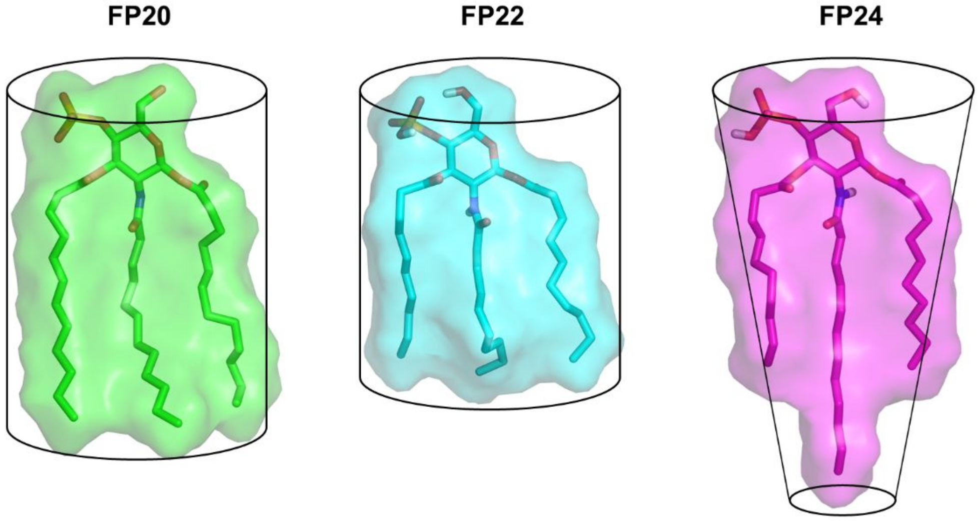

Figure S15. Three-dimensional conformations of the FP20, FP22, and FP24 compounds in water. The active ligands **FP20** and **FP22** adopt cylindrical shape, whereas **FP24** displays an inverted-cone conformation.

Biological Characterization

In vitro Supporting Data

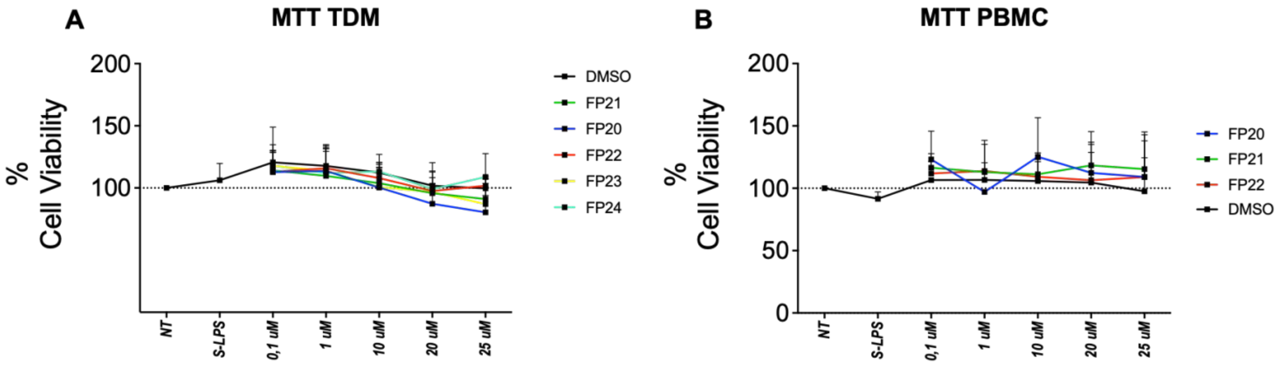

Figure S16. Cell viability (A) THP-1 derived macrophages (TDM) were treated with the shown concentrations of FP20-24 for 16-18 hours. **(B)** PBMCs were treated with shown concentrations of FP20-22 for 16-18 hours. In **(A)** and **(B)** cell viability was tested using MTT assay. 100% cell viability was attributed to the negative control (NT). Results are shown as mean ± SEM of at least three independent experiments.

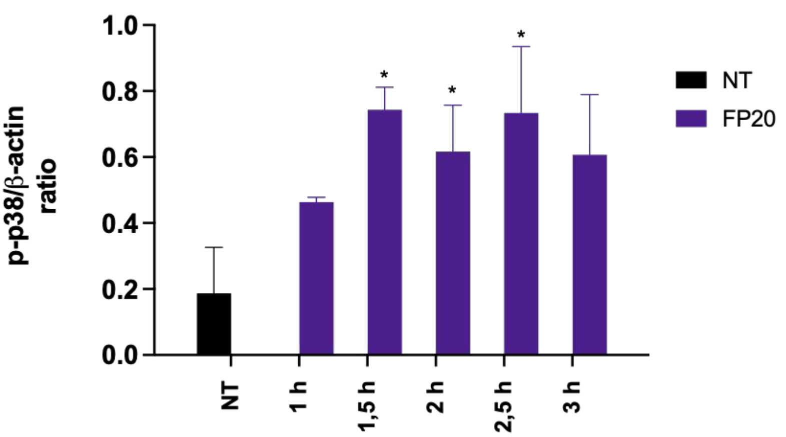

Figure S17. Western blot analysis of p38 activation. Differentiated THP1-XBlueTM cells were treated with 25 μM of FP20 for 0 to 3 hours. p38 was detected by western blot and its relative amount was calculated in respect to housekeeping protein β-actin. Densitometric analysis was carried out using Image J. Data are expressed as mean ± SEM of at least three independent experiments. (Treated Vs Non- treated: *P<0.05; **P<0.01; ***P<0.001; ****P<0.0001).

In vivo Supporting Data

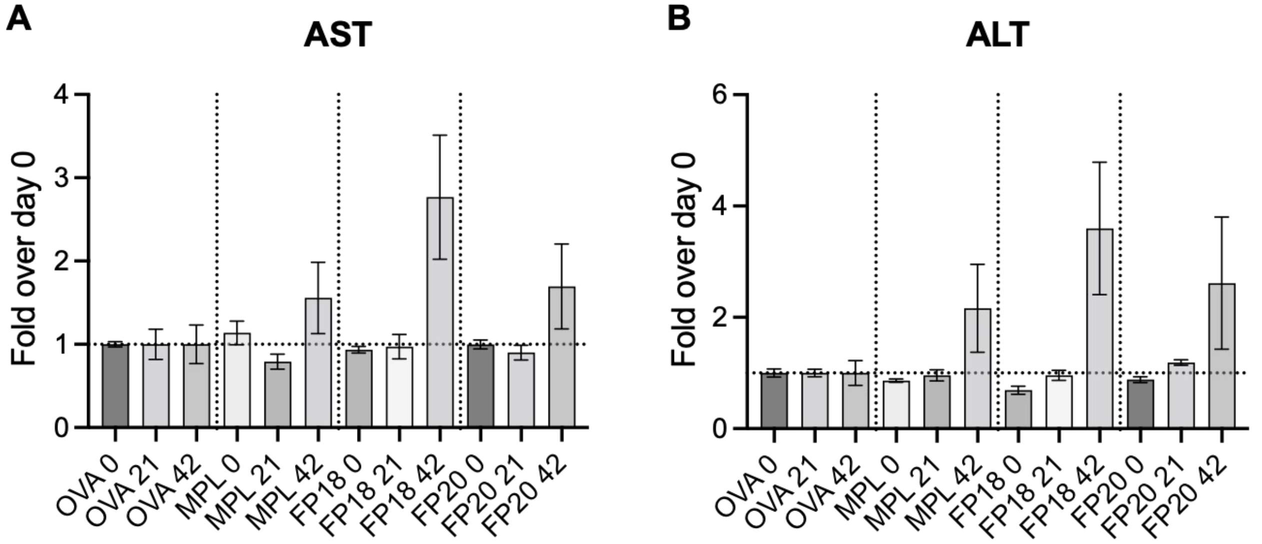

Figure S18. Liver transaminases of C57BL/6 mice immunized with FP18 and FP20 (A) Aspartate transaminase (AST) and **(B)** Alanine transaminase (ALT) relative to the OVA control group in mice serum 0-, 21- and 42-days post-immunization.

Bibliography

(1) Ma, H.; Cummins, D. D.; Edelstein, N. B.; Gomez, J.; Khan, A.; Llewellyn, M. D.; Picudella, T.; Willsey, S. R.; Nangia, S. Modeling Diversity in Structures of Bacterial Outer Membrane Lipids. *J. Chem. Theory Comput.* **2017**, *13* (2), 811–824. DOI: 10.1021/acs.jctc.6b00856.

(2) Oleg, T.; Arthur J., O. AutoDock Vina: Improving the Speed and Accuracy of Docking with a New Scoring Function, Efficient Optimization, and Multithreading. *J. Comput. Chem.* **2010**, *31*(2):455-461. DOI:10.1002/jcc.21334.

(3) Cochet, F.; Facchini, F. A.; Zaffaroni, L.; Billod, J. M.; Coelho, H.; Holgado, A.; Braun, H.; Beyaert, R.; Jerala, R.; Jimenez-Barbero, J.; Martin-Santamaria, S.; Peri, F. Novel Carboxylate-Based Glycolipids: TLR4 Antagonism, MD-2 Binding and Self-Assembly Properties. *Sci. Rep.* **2019**, *9* (1), 919. DOI: 10.1038/S41598-018-37421-W.

(4) Facchini, F. A.; Minotti, A.; Luraghi, A.; Romerio, A.; Gotri, N.; Matamoros-Recio, A.; Iannucci, A.; Palmer, C.; Wang, G.; Ingram, R.; Martin-Santamaria, S.; Pirianov, G.; De Andrea, M.; Valvano, M. A.; Peri, F. Synthetic Glycolipids as Molecular Vaccine Adjuvants: Mechanism of Action in Human Cells and in Vivo Activity. *J. Med. Chem.* **2021**, *64* (16), 12261–12272. DOI: 10.1021/acs.jmedchem.1c00896.

(5) Park, B. S.; Song, D. H.; Kim, H. M.; Choi, B. S.; Lee, H.; Lee, J. O. The Structural Basis of Lipopolysaccharide Recognition by the TLR4-MD-2 Complex. *Nature* **2009**, *458* (7242), 1191–1195. DOI: 10.1038/nature07830.

(6) Morris, G. M.; Ruth, H.; Lindstrom, W.; Sanner, M. F.; Belew, R. K.; Goodsell, D. S.; Olson, A. J. AutoDock4 and AutoDockTools4: Automated Docking with Selective Receptor Flexibility. *J. Comput. Chem.* **2009**, *30* (16), 2785–2791. DOI: 10.1002/JCC.21256.

(7) Netea, M. G.; Van Deuren, M.; Kullberg, B. J.; Cavaillon, J. M.; Van Der Meer, J. W. M. Does the Shape of Lipid A Determine the Interaction of LPS with Toll-like Receptors? *Trends Immunol.* **2002**, *23* (3), 135–139. DOI: 10.1016/S1471-4906(01)02169-X.

(8) Seydel, U.; Hawkins, L.; Schromm, A. B.; Heine, H.; Scheel, O.; Koch, M. H. J.; Brandenburg, K. The Generalized Endotoxic Principle. *Eur. J. Immunol.* **2003**, *33* (6), 1586–1592. DOI: 10.1002/EJI.200323649.

NMR Spectra

Compound FP20

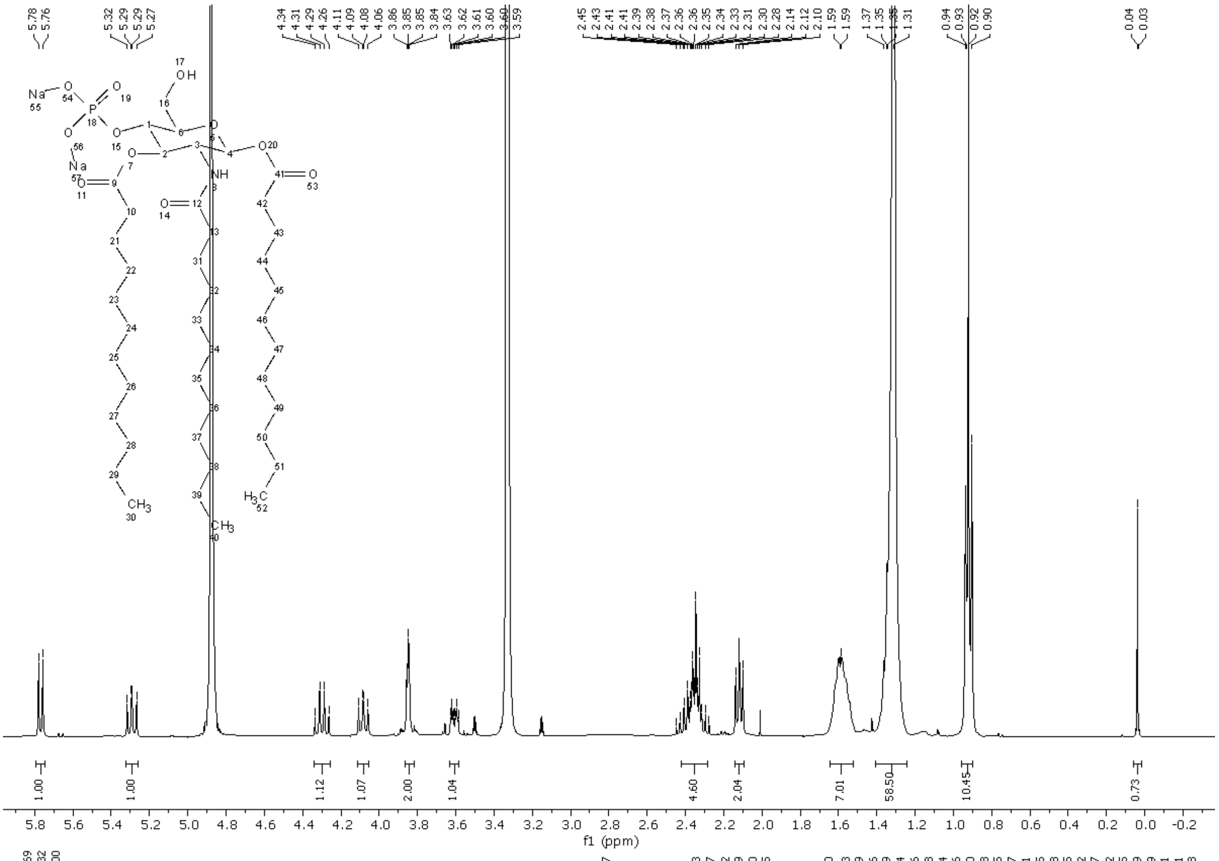

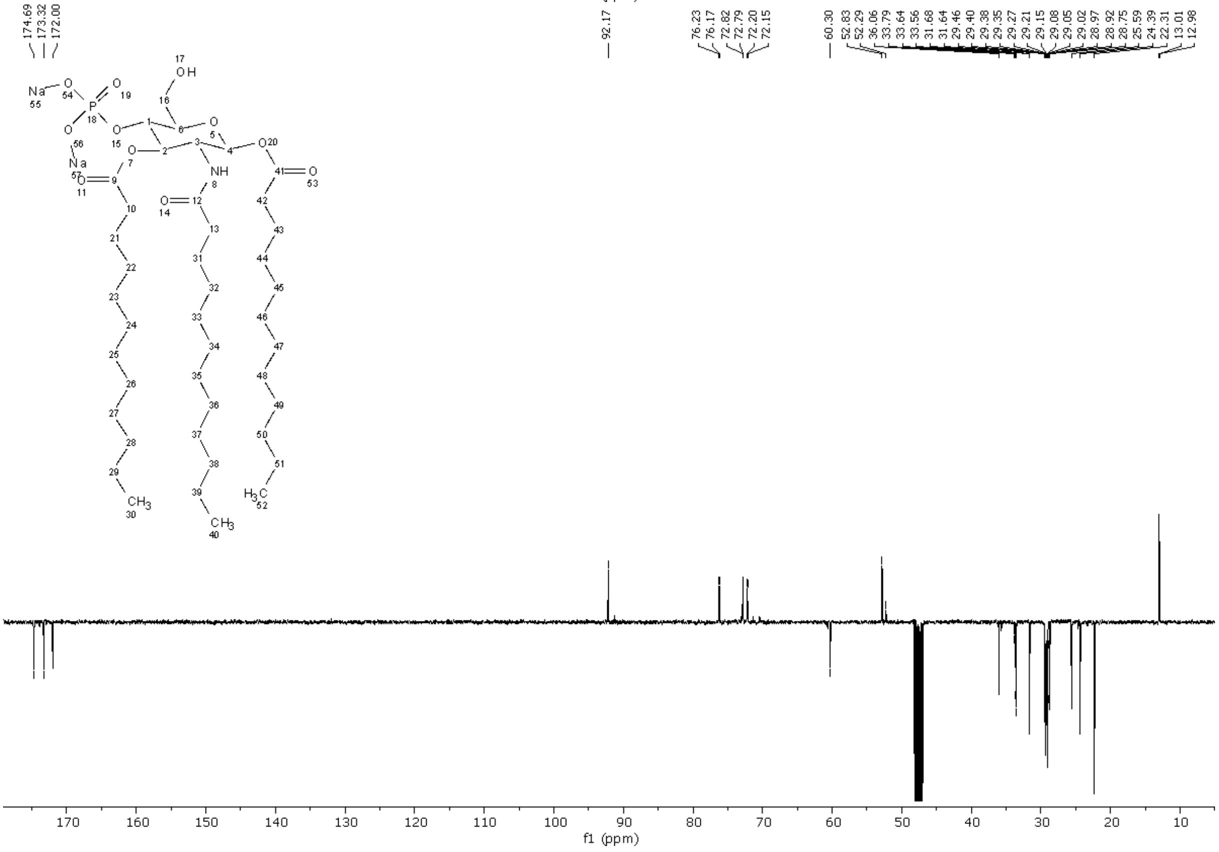

Compound FP21

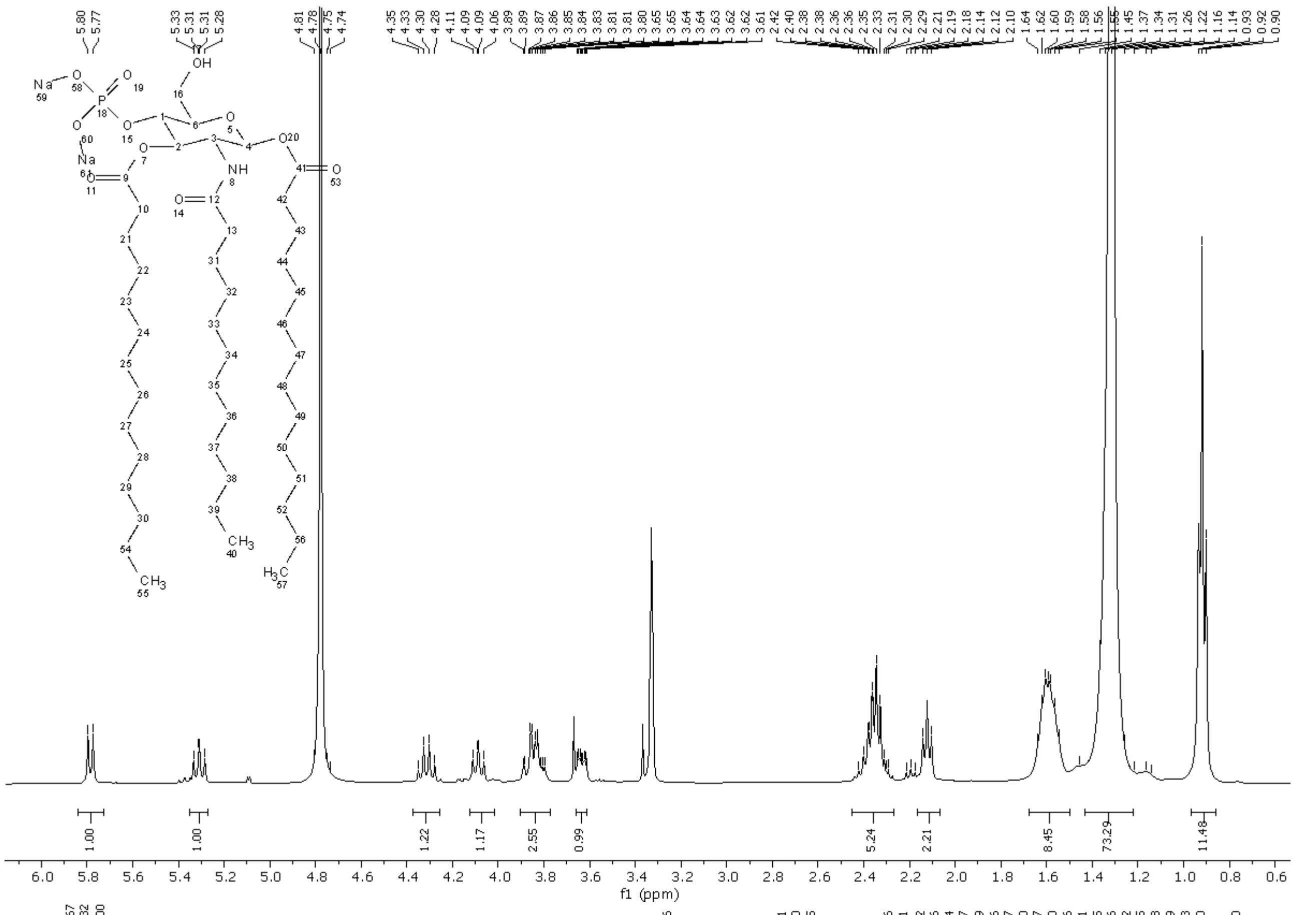

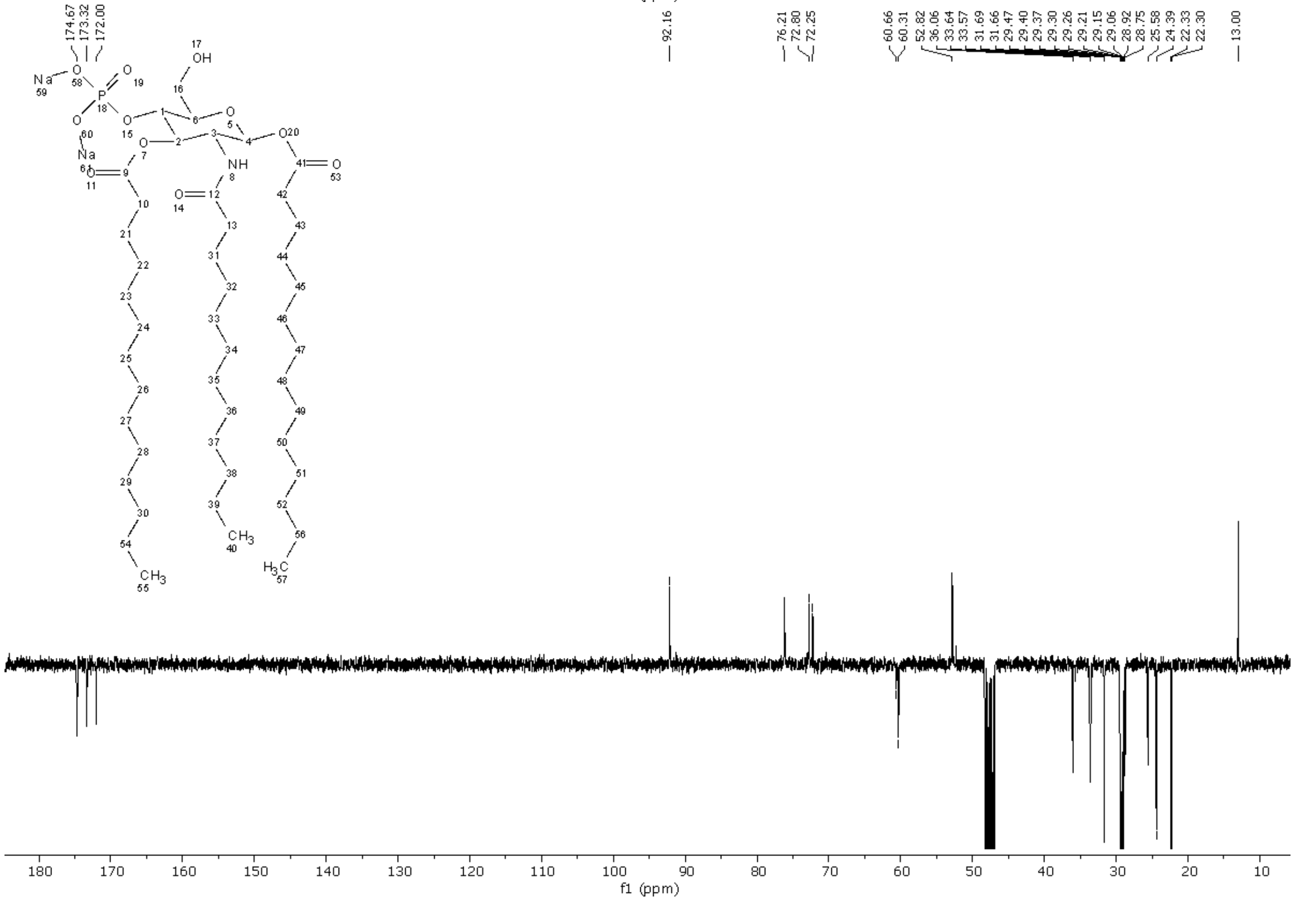

Compound FP22

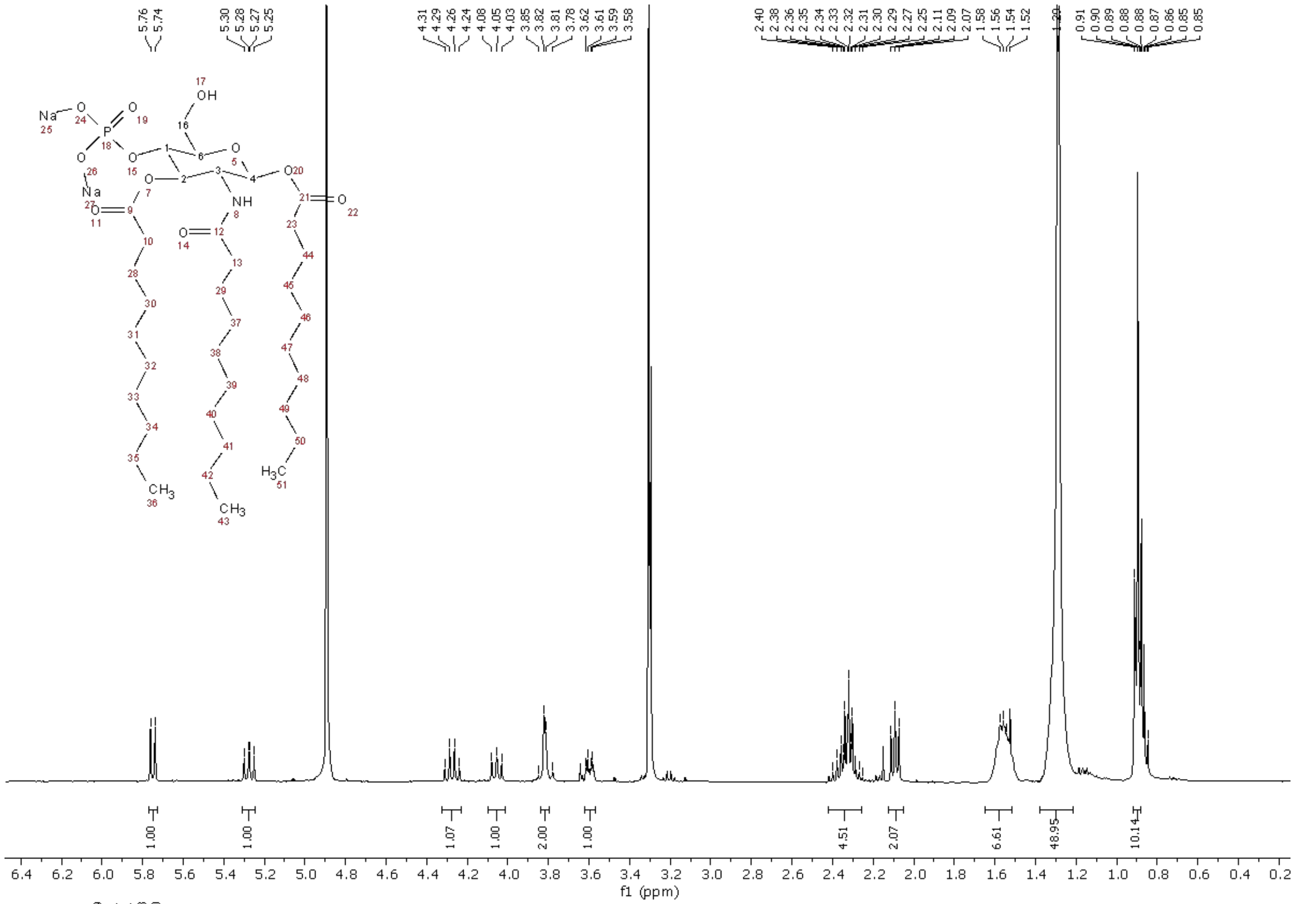

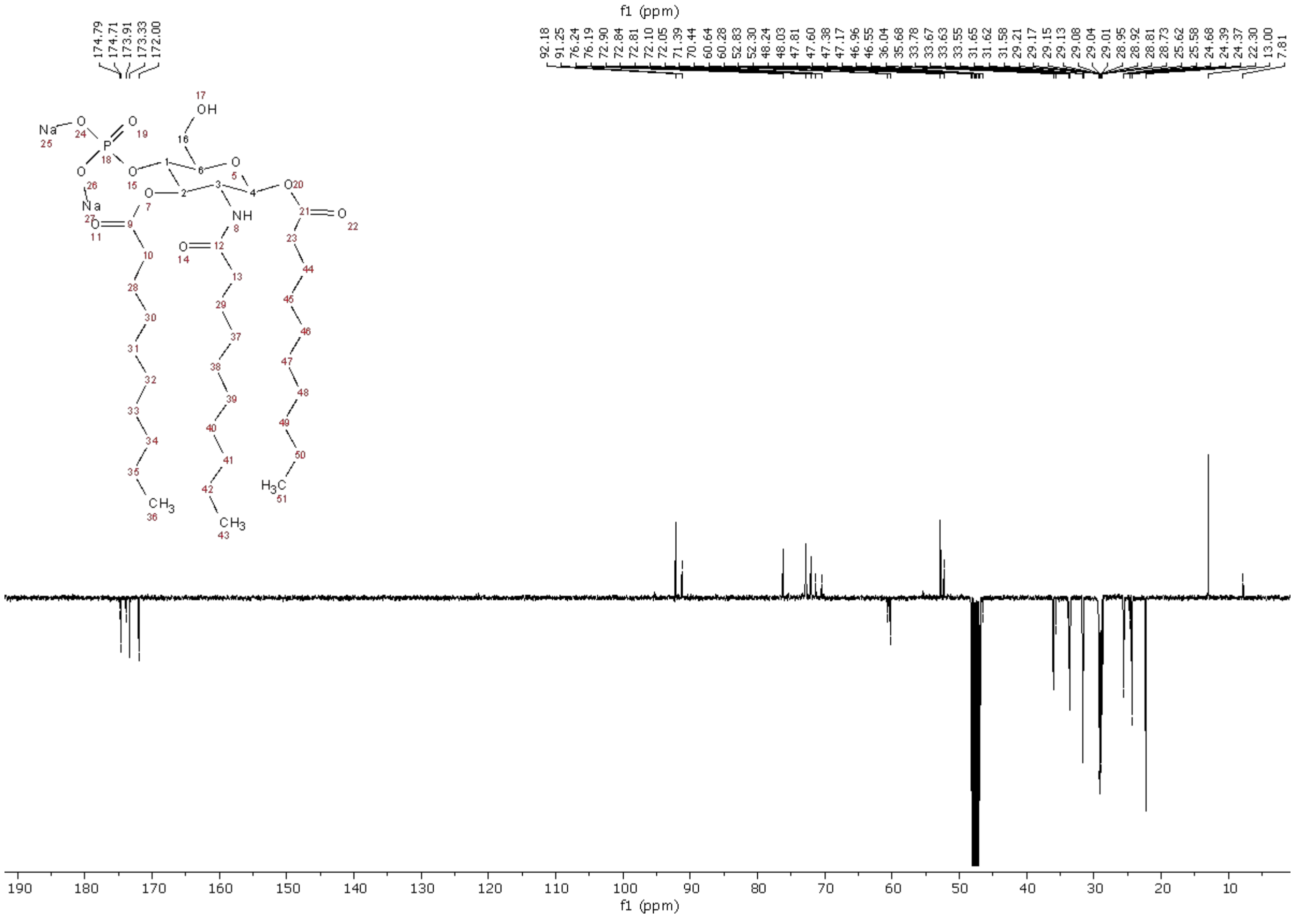

Compound FP23

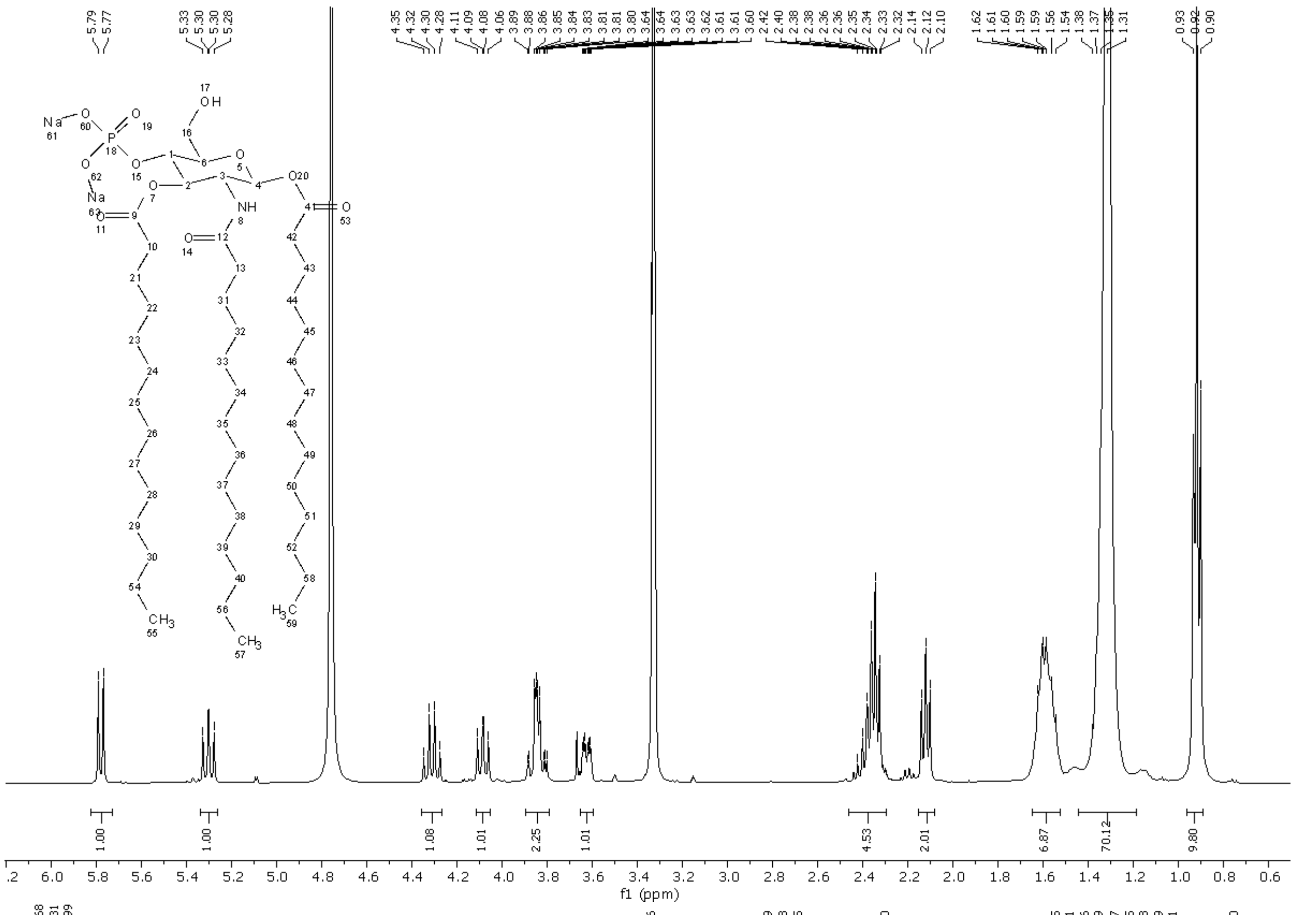

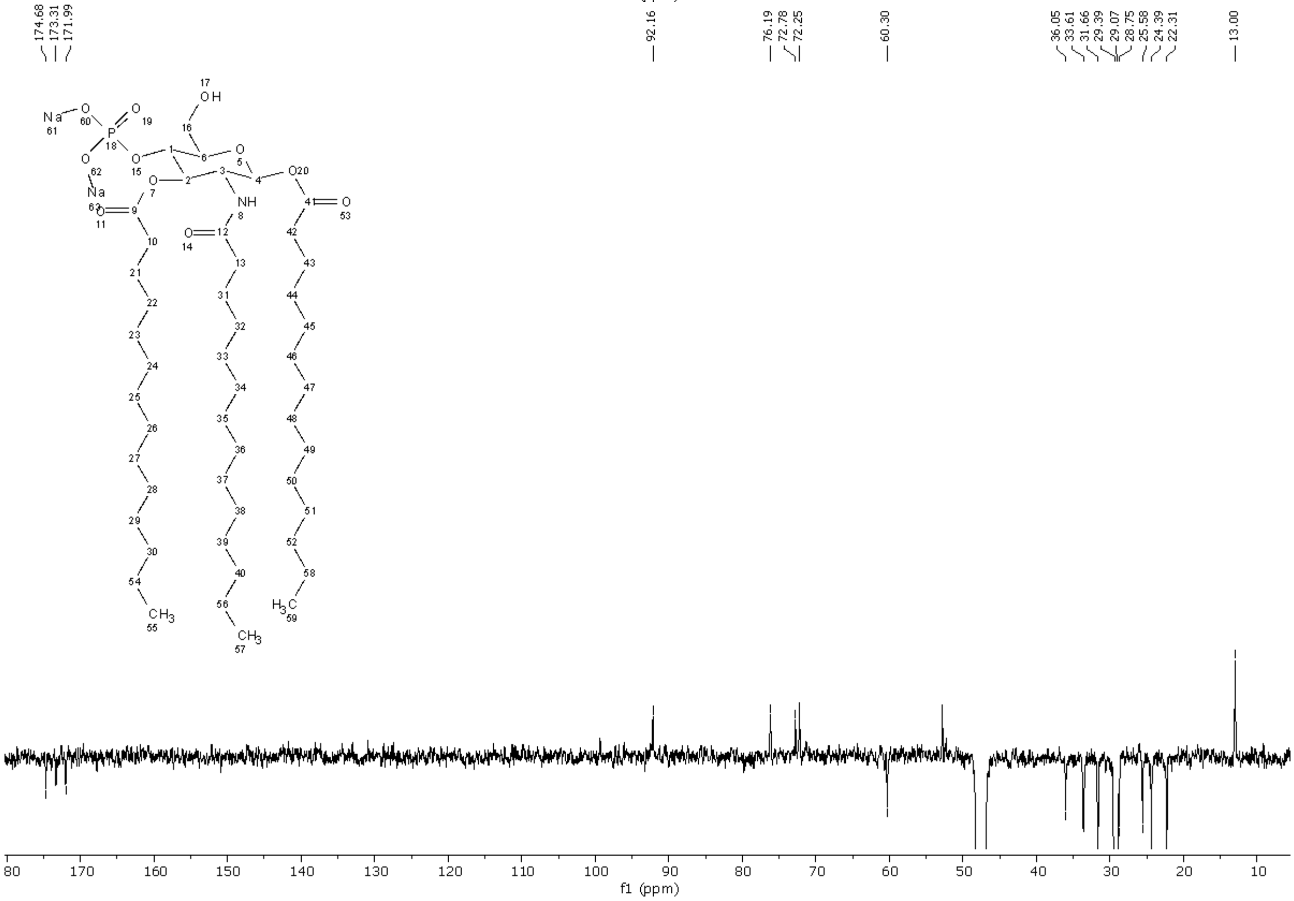

Compound FP24

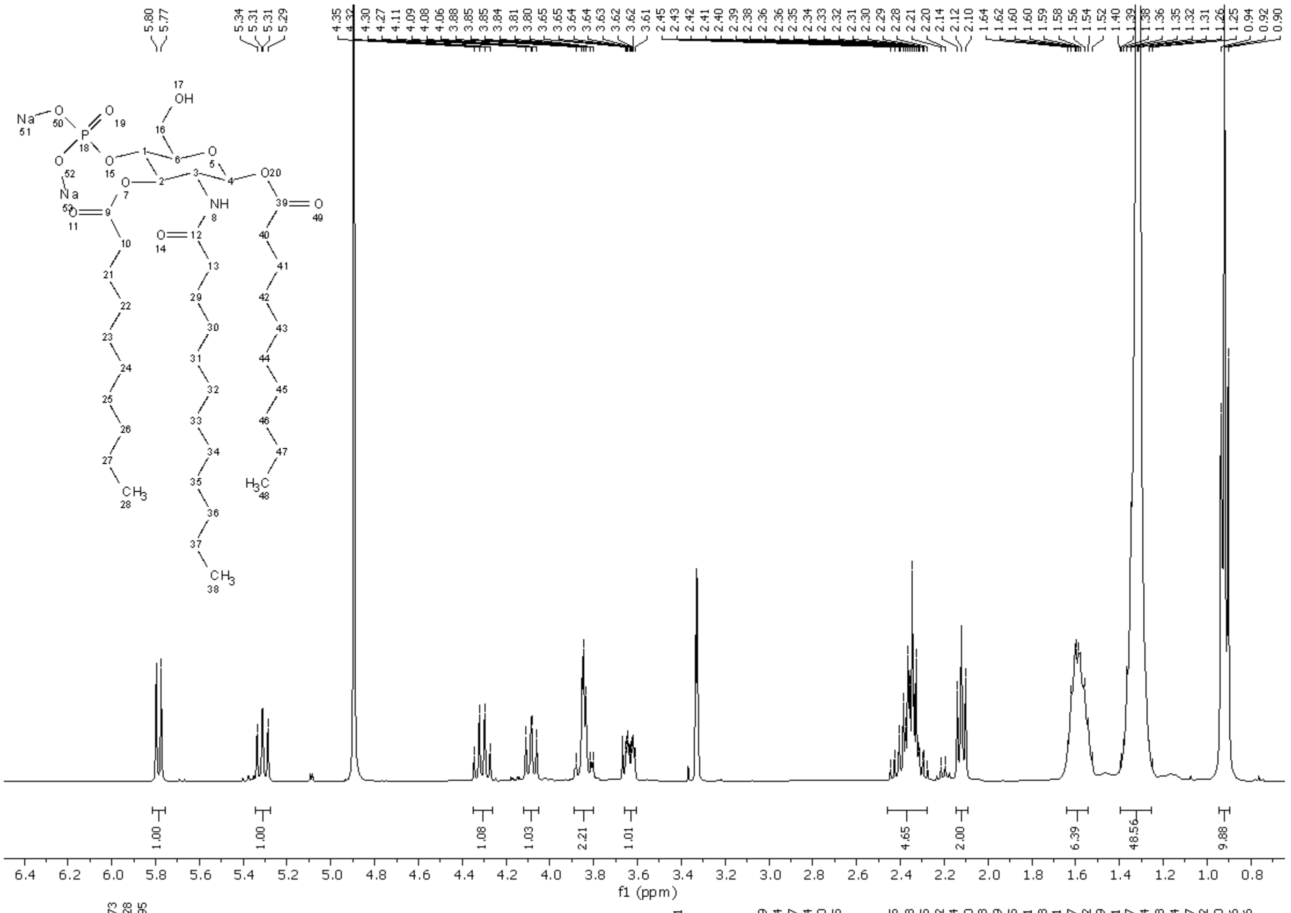

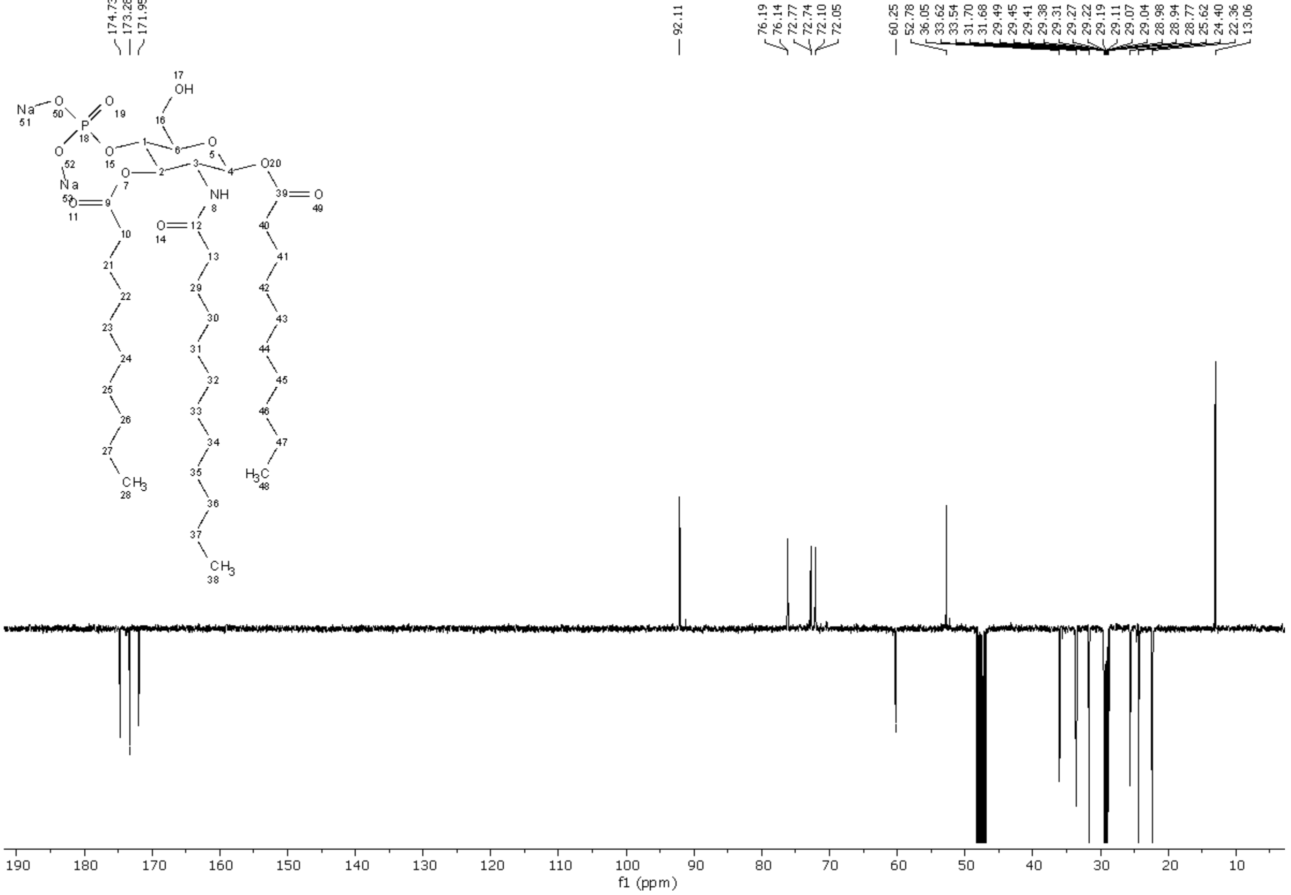

Compound α-FP20

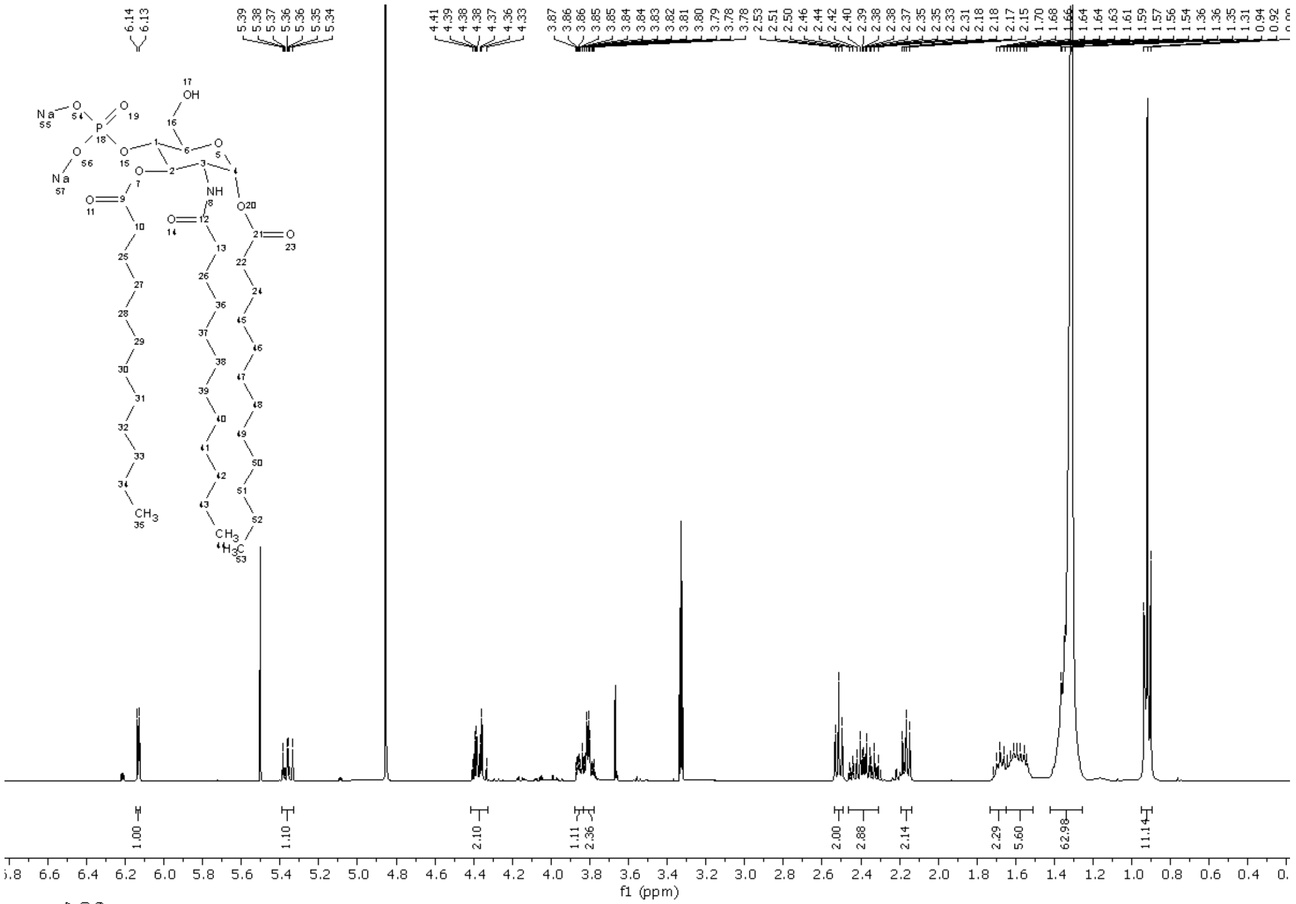

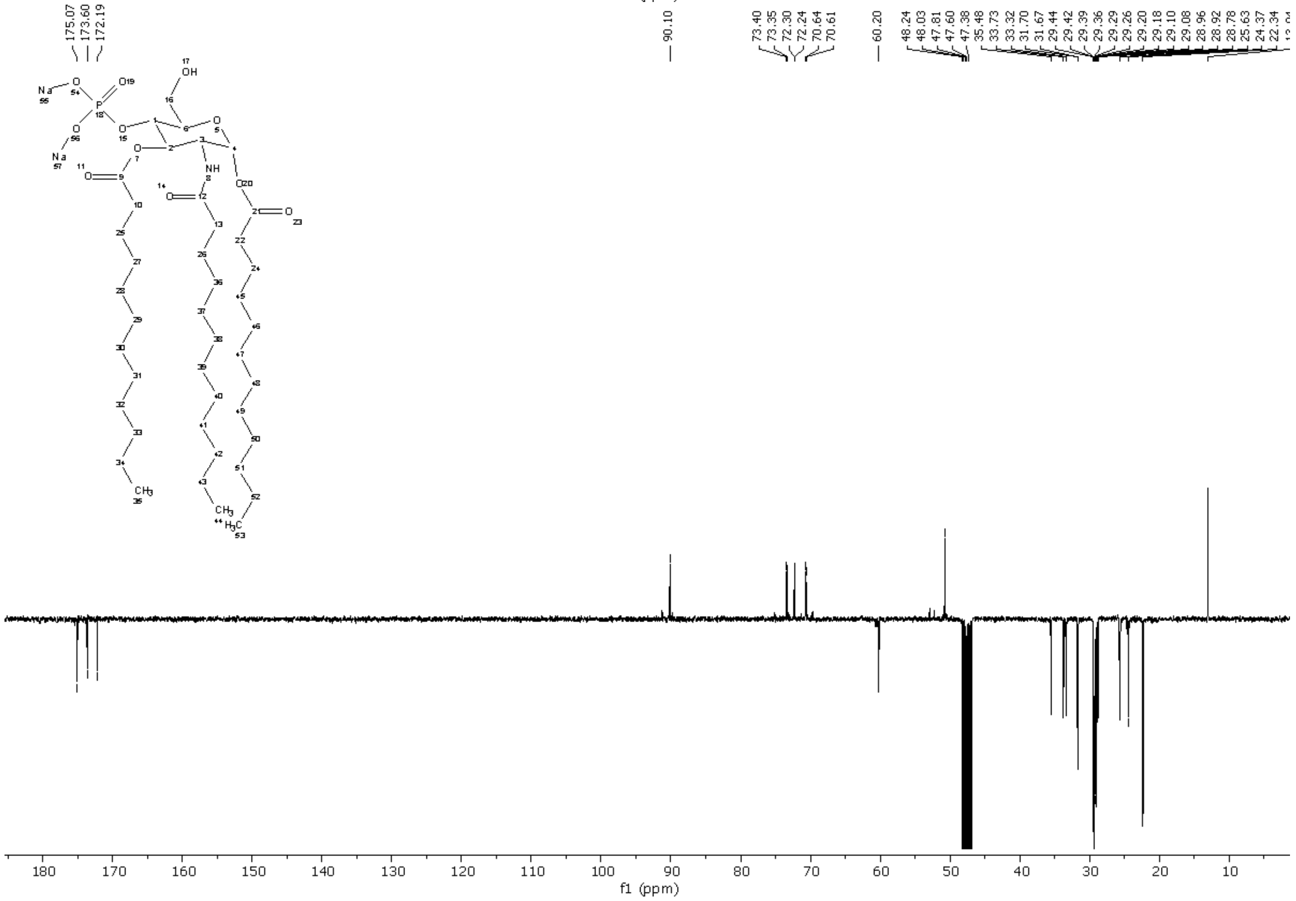

Compound 2a

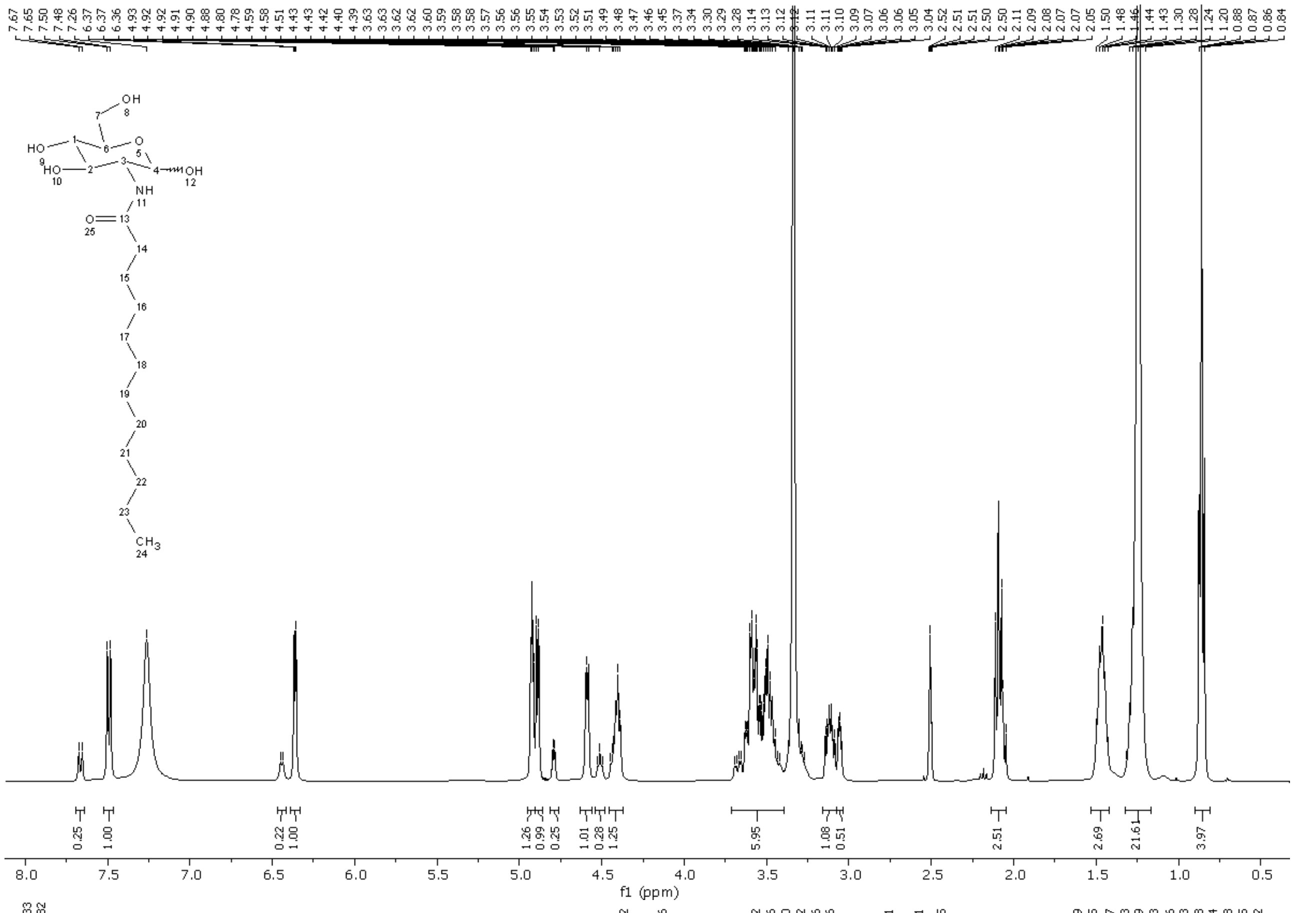

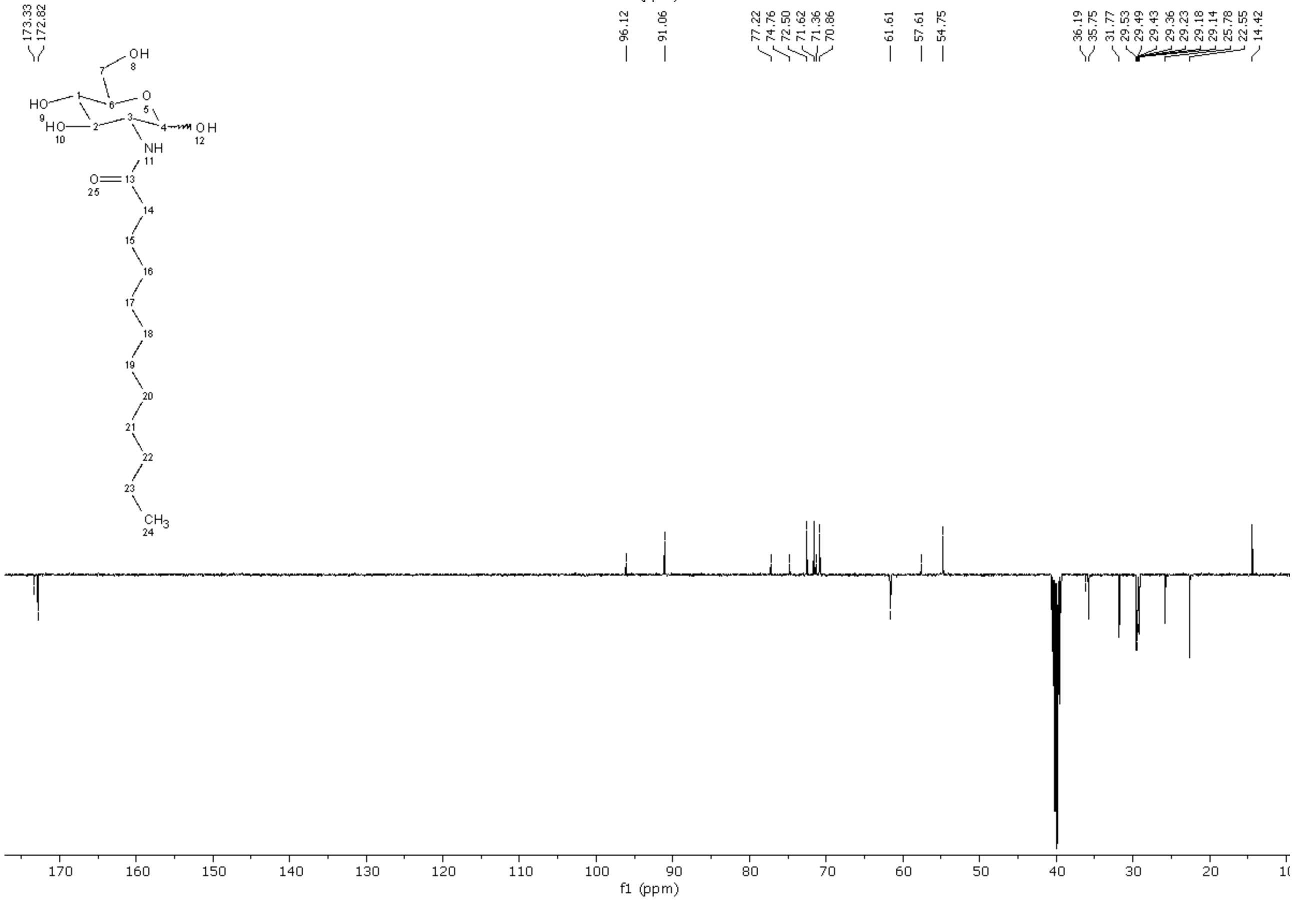

Compound 3a

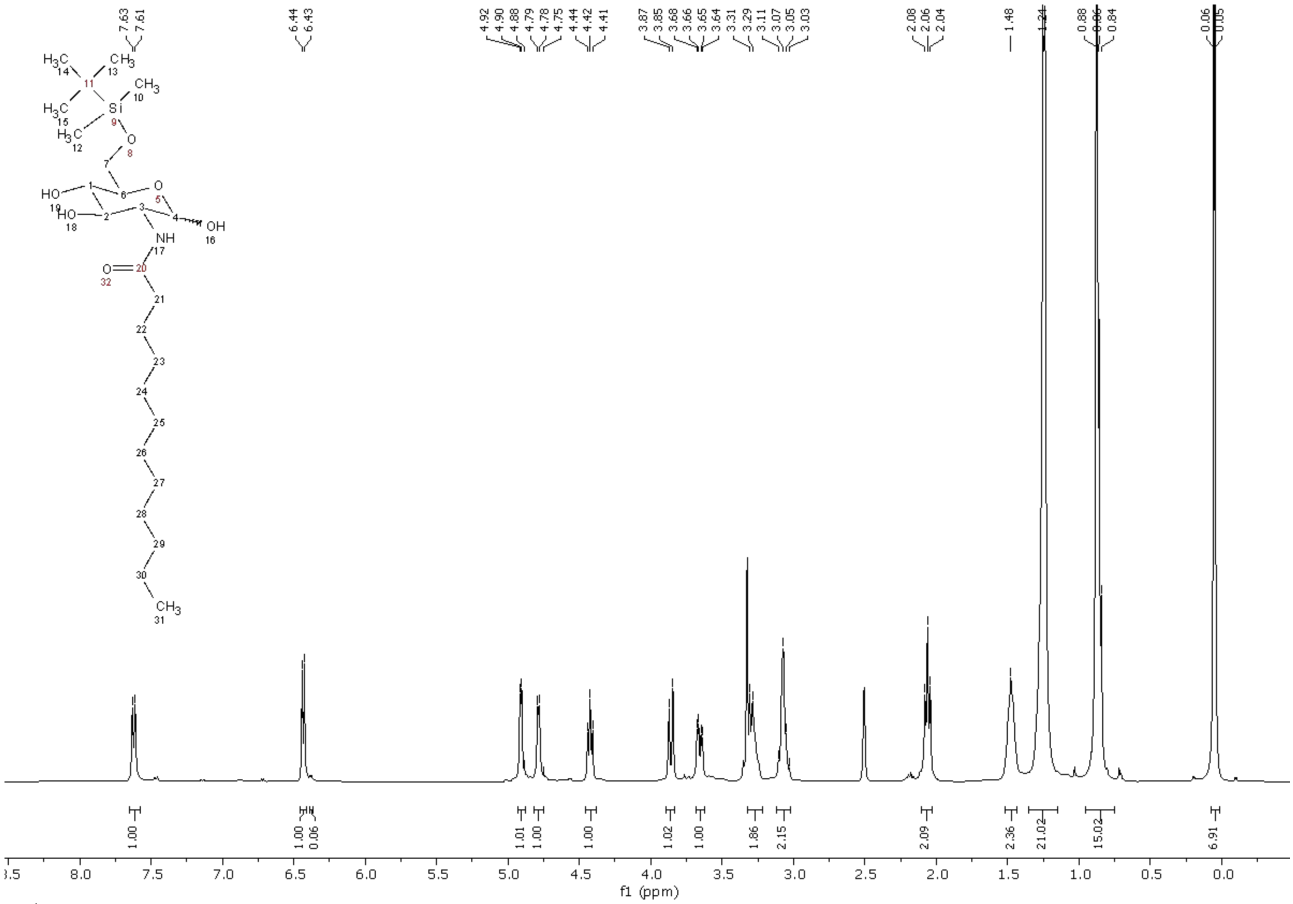

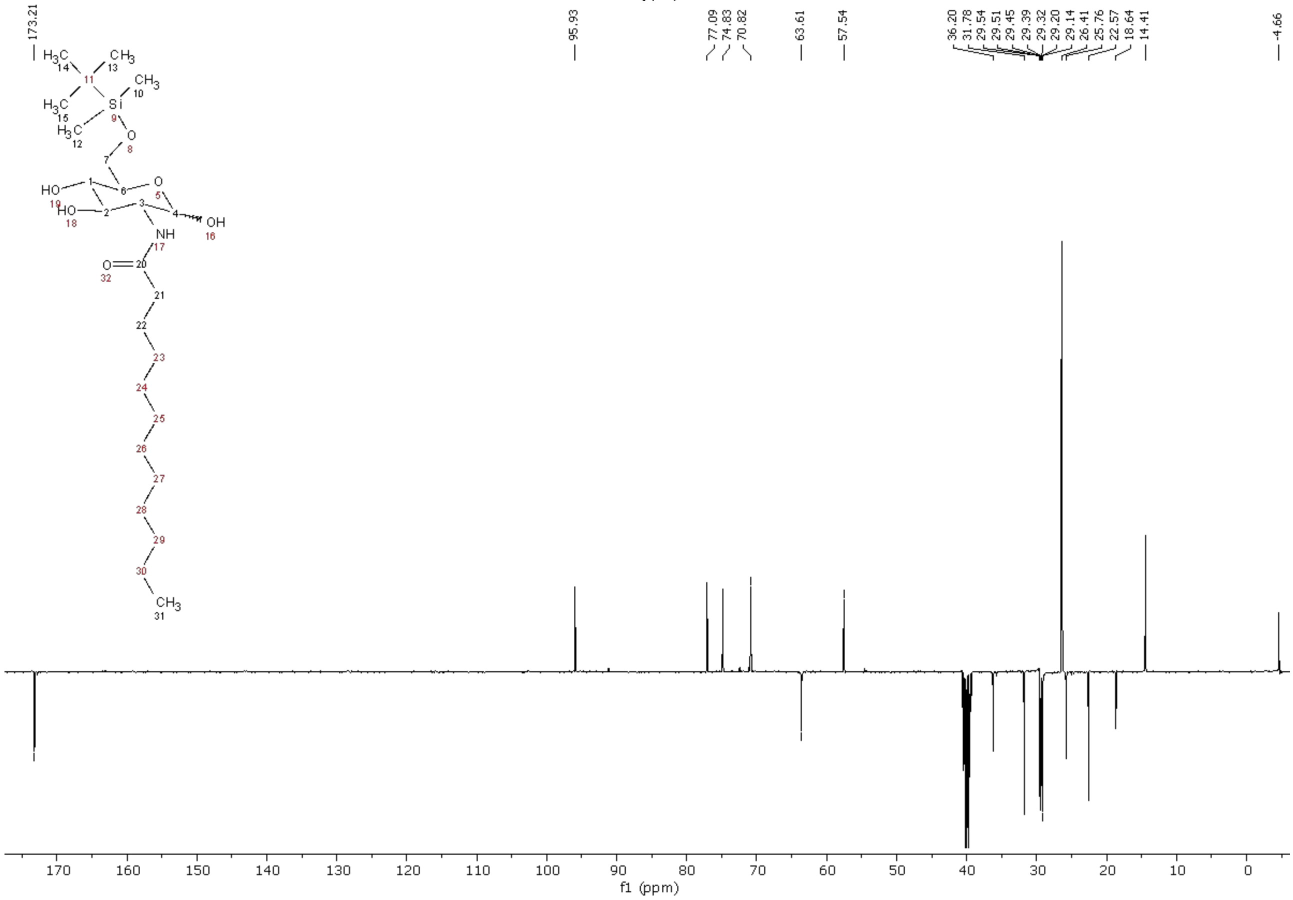

Compound 4a

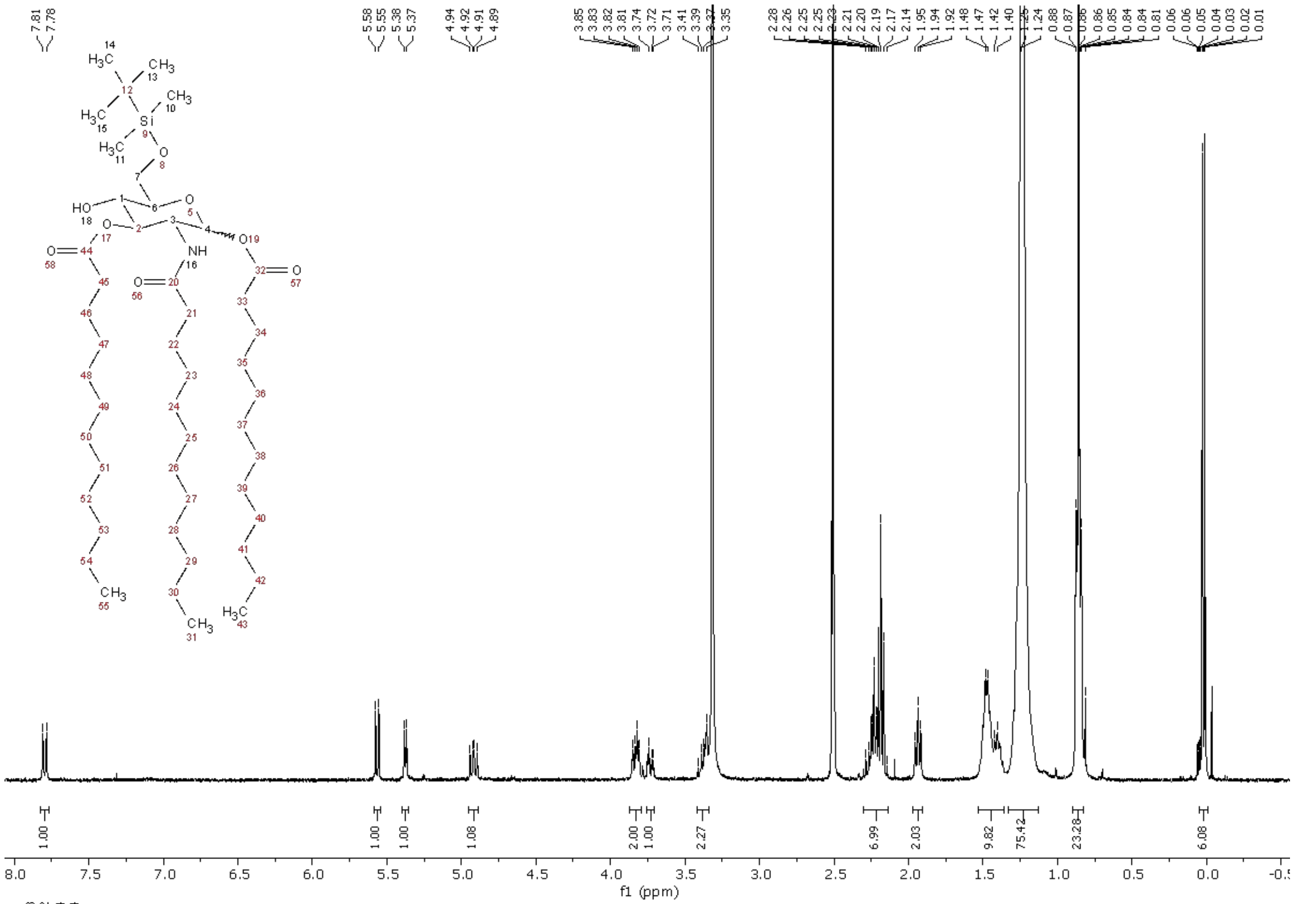

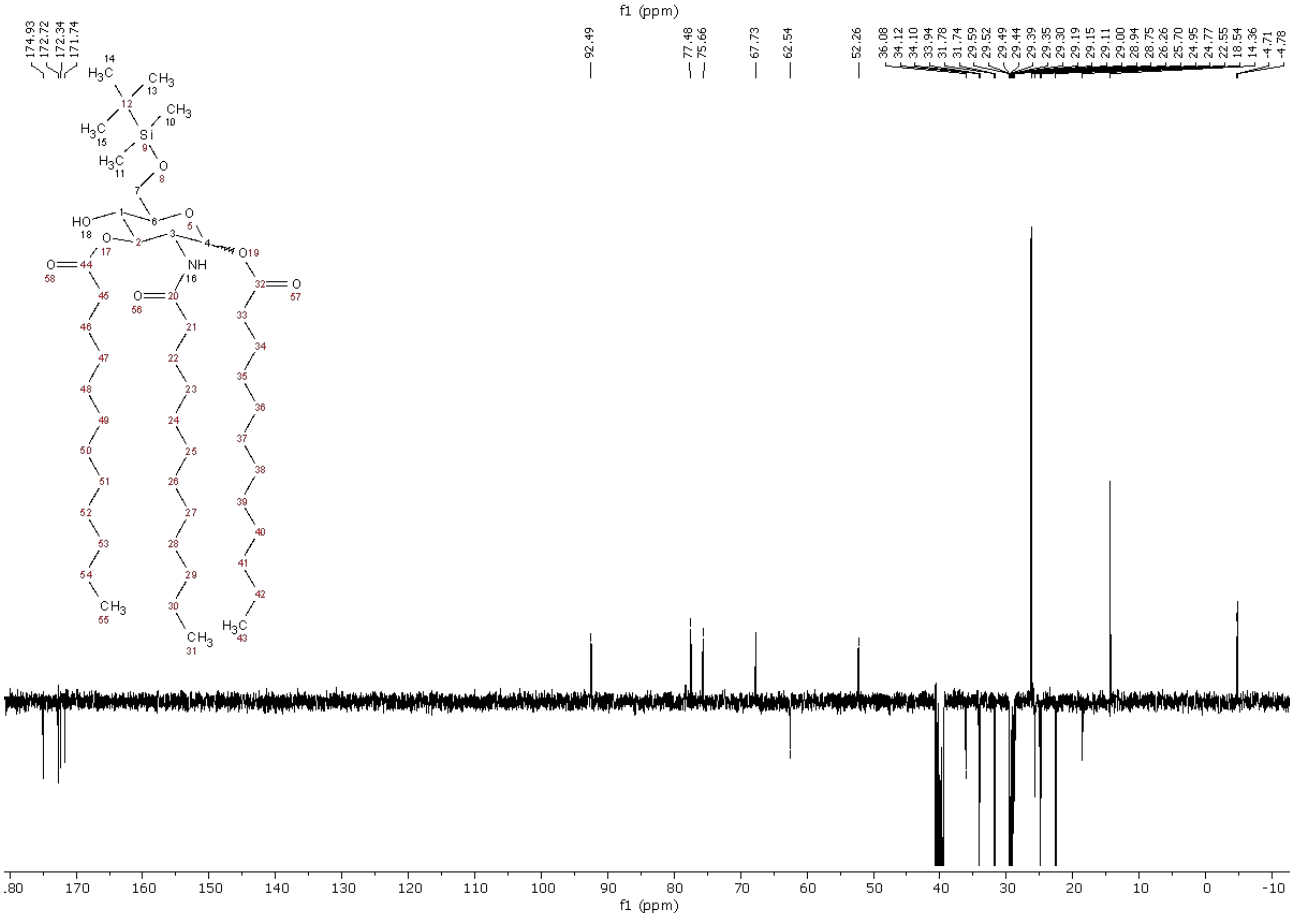

Compound 5a

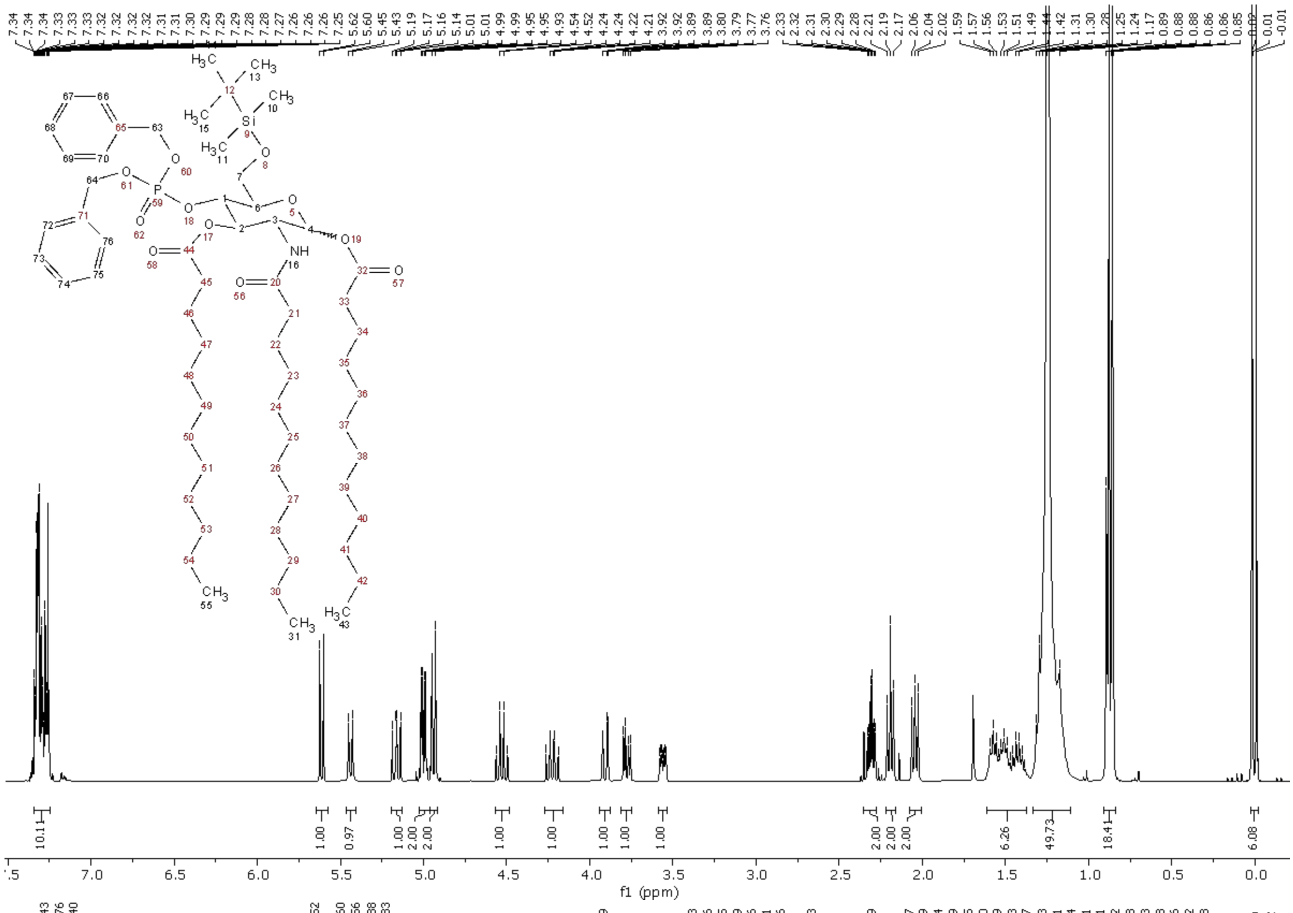

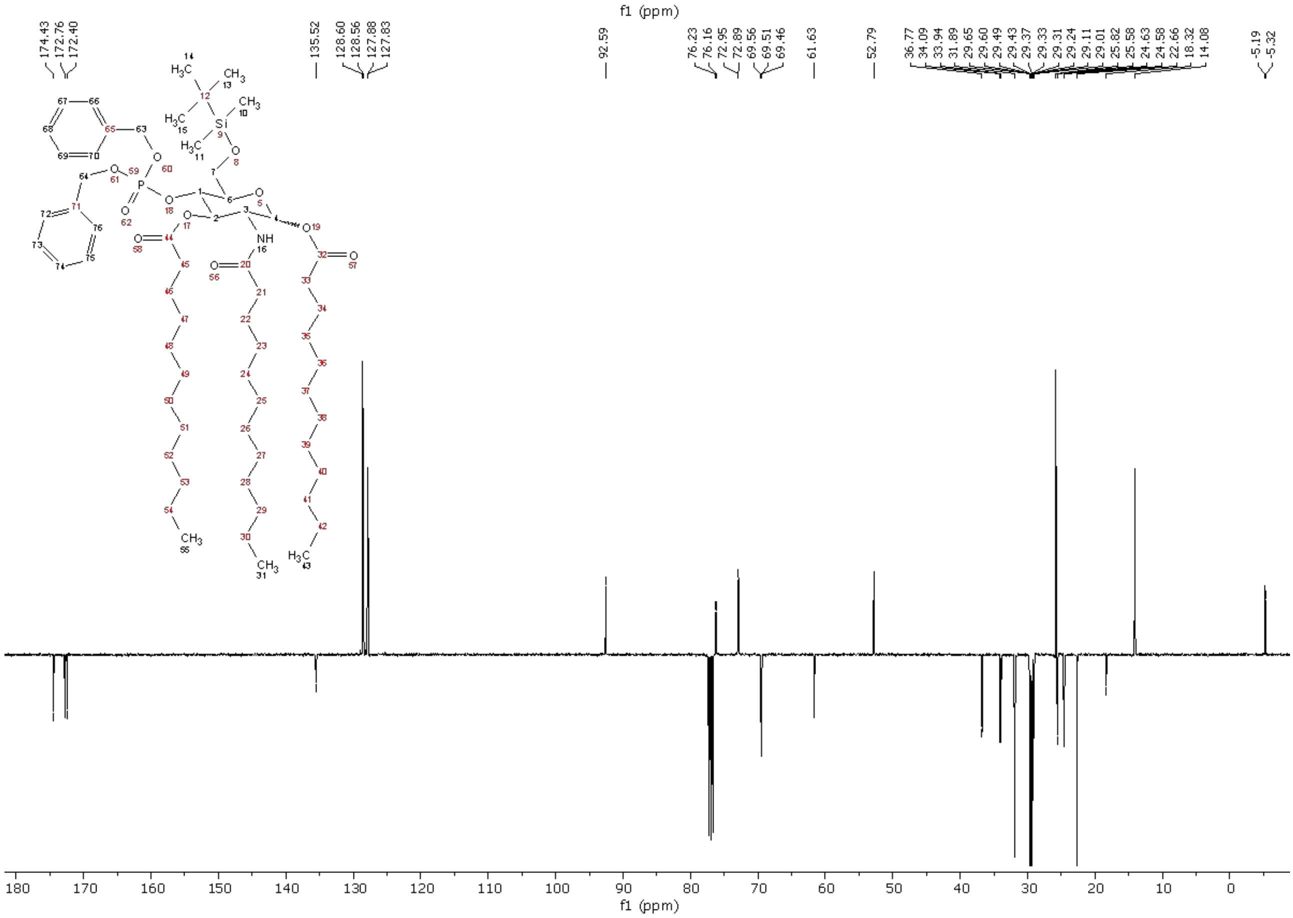

Compound 6a

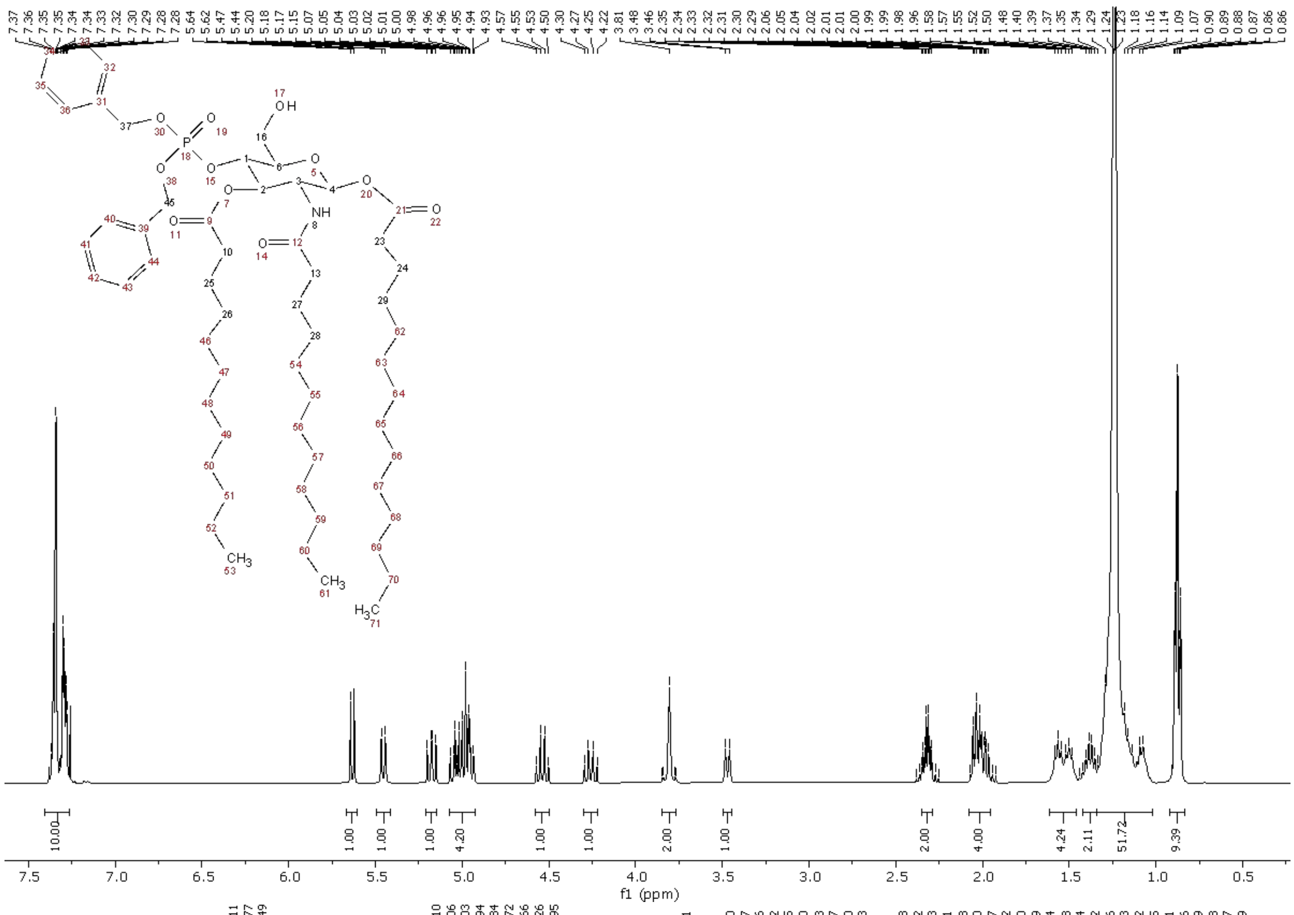

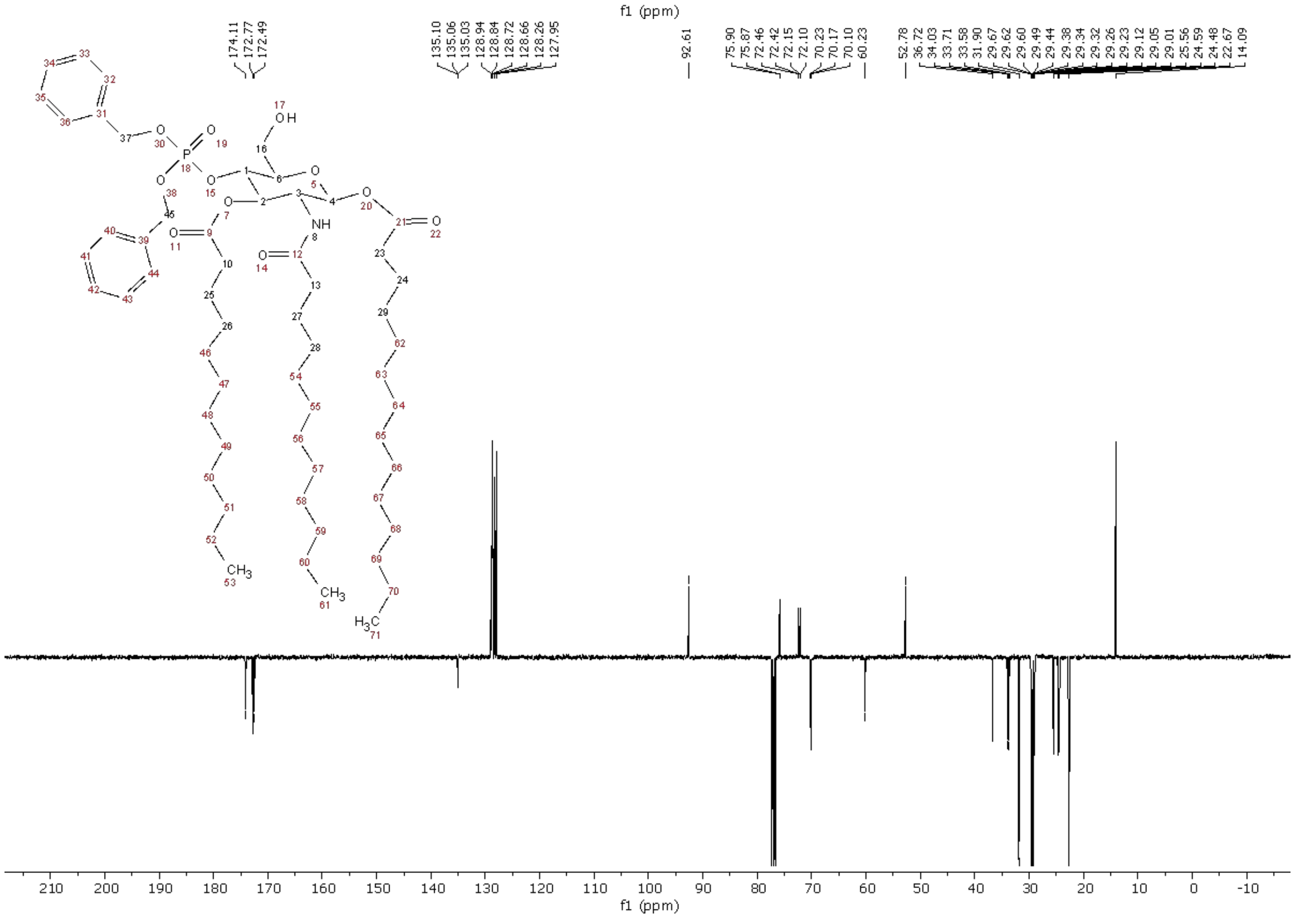

Compound 7

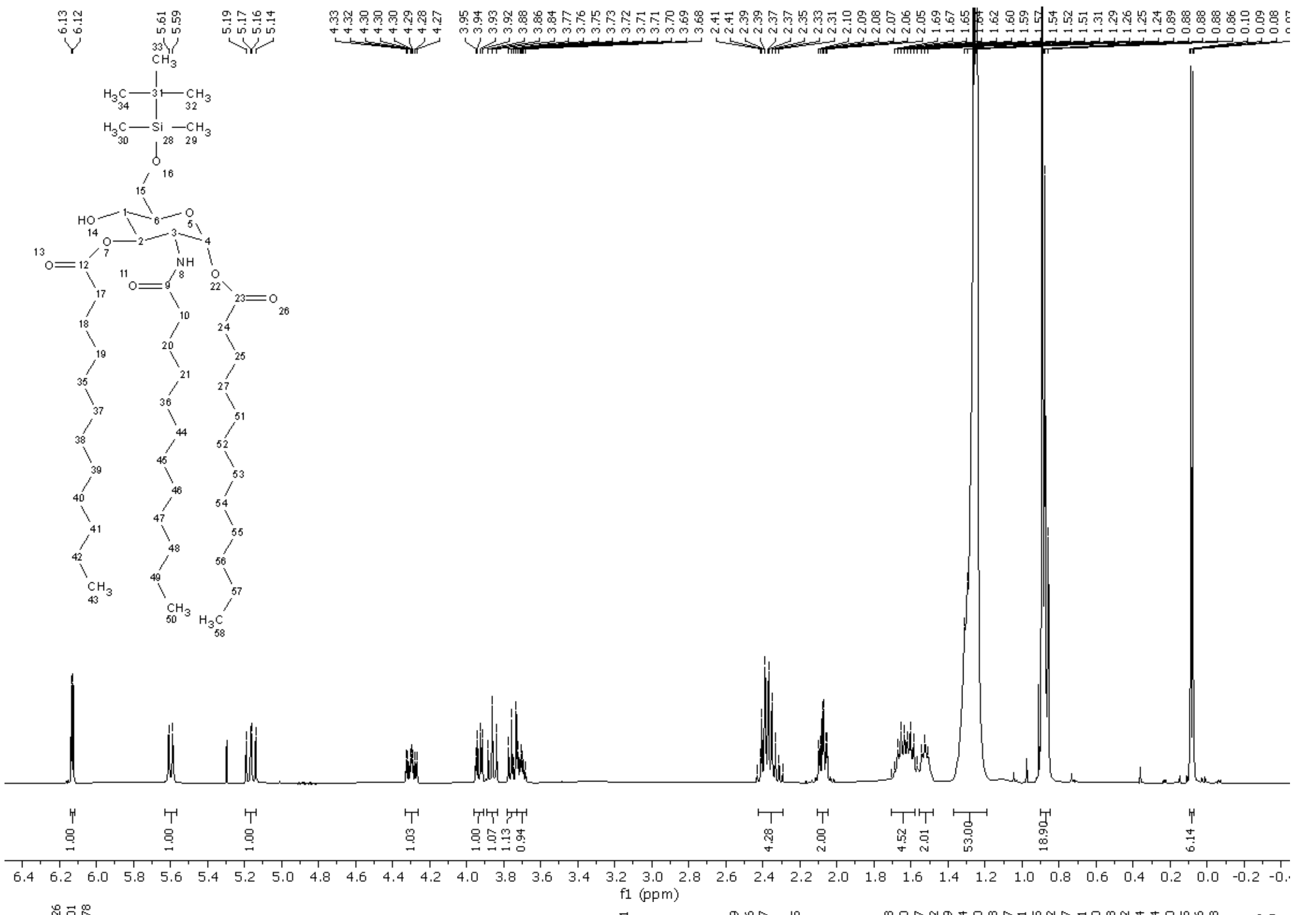

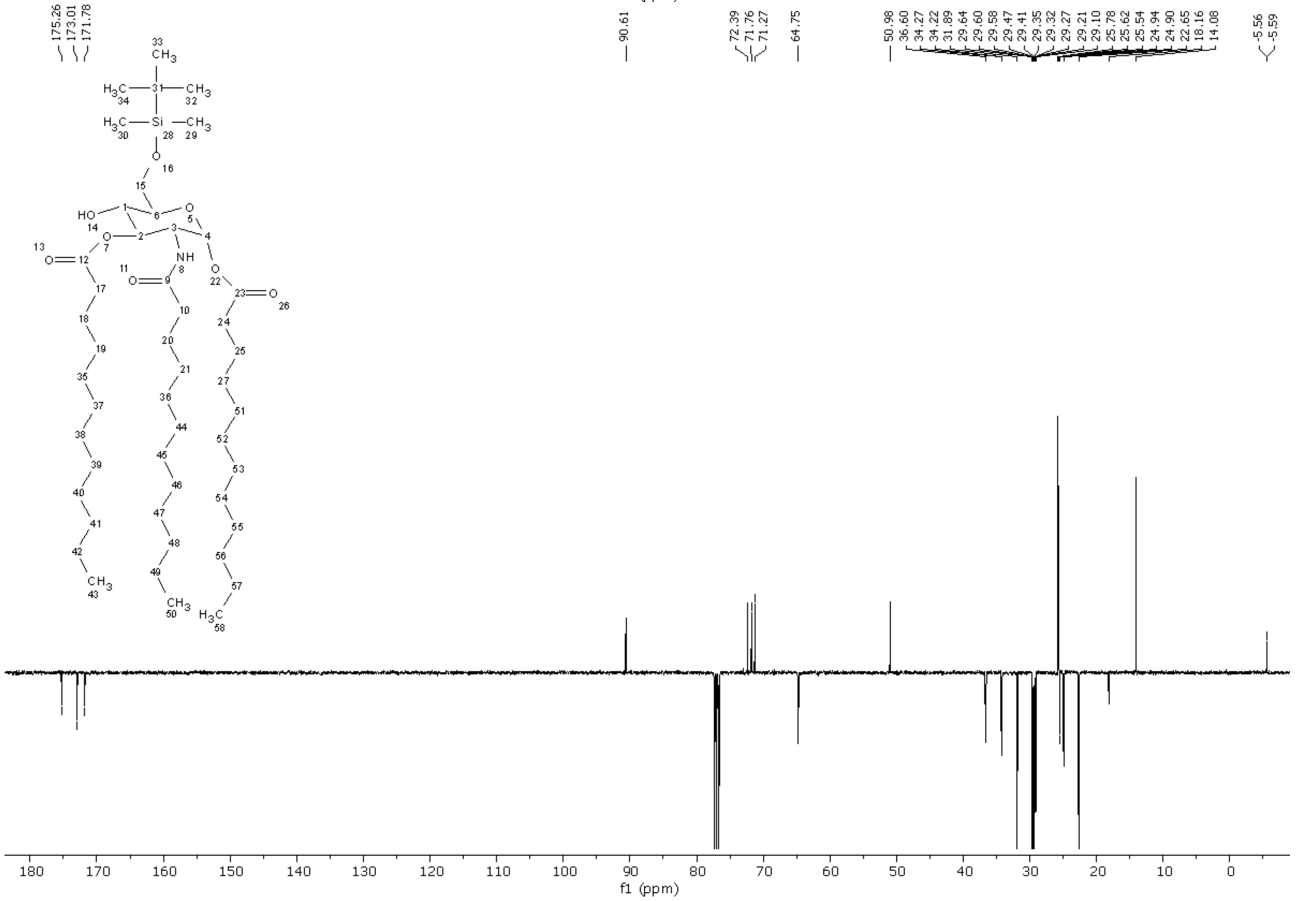

Compound 8

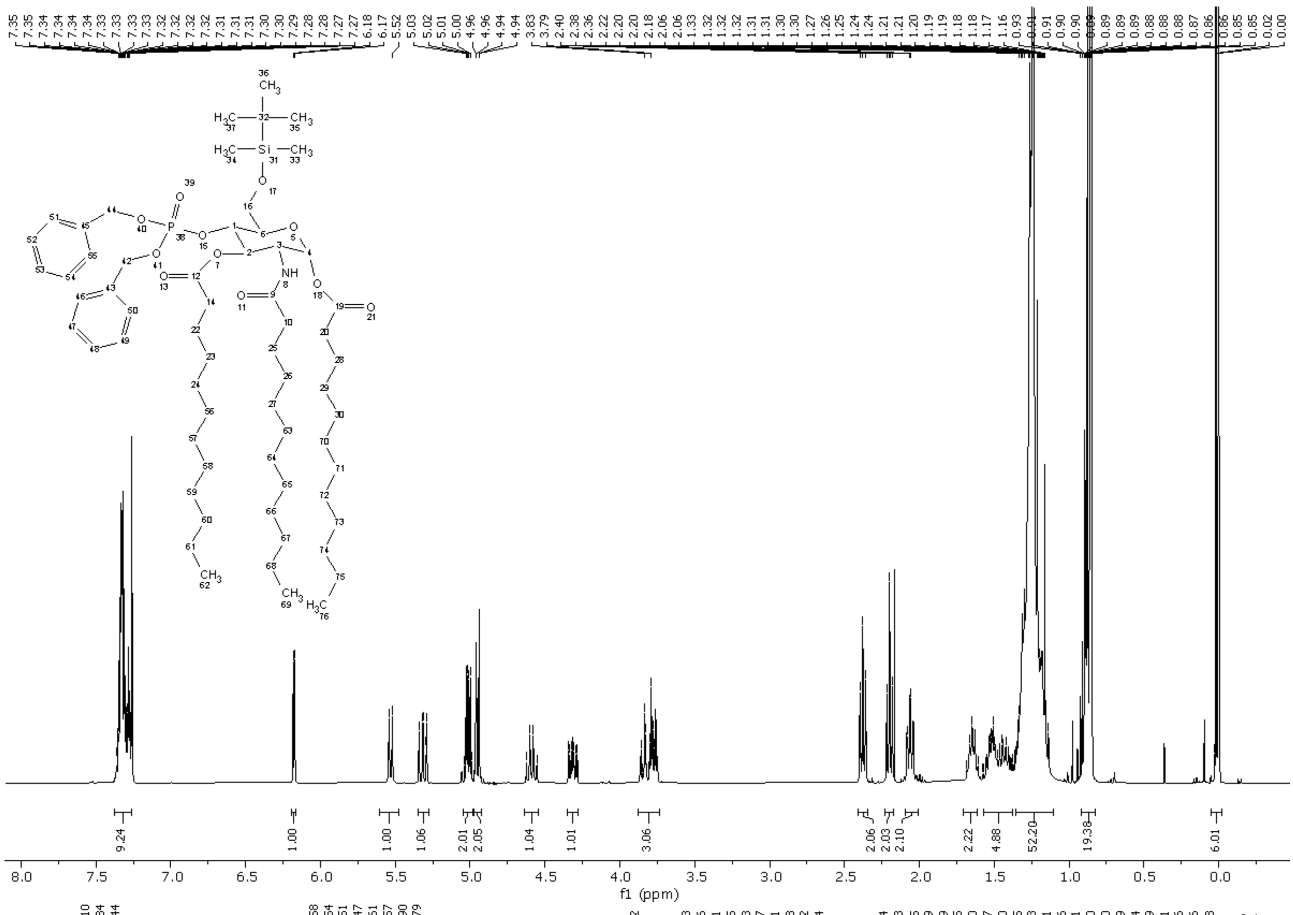

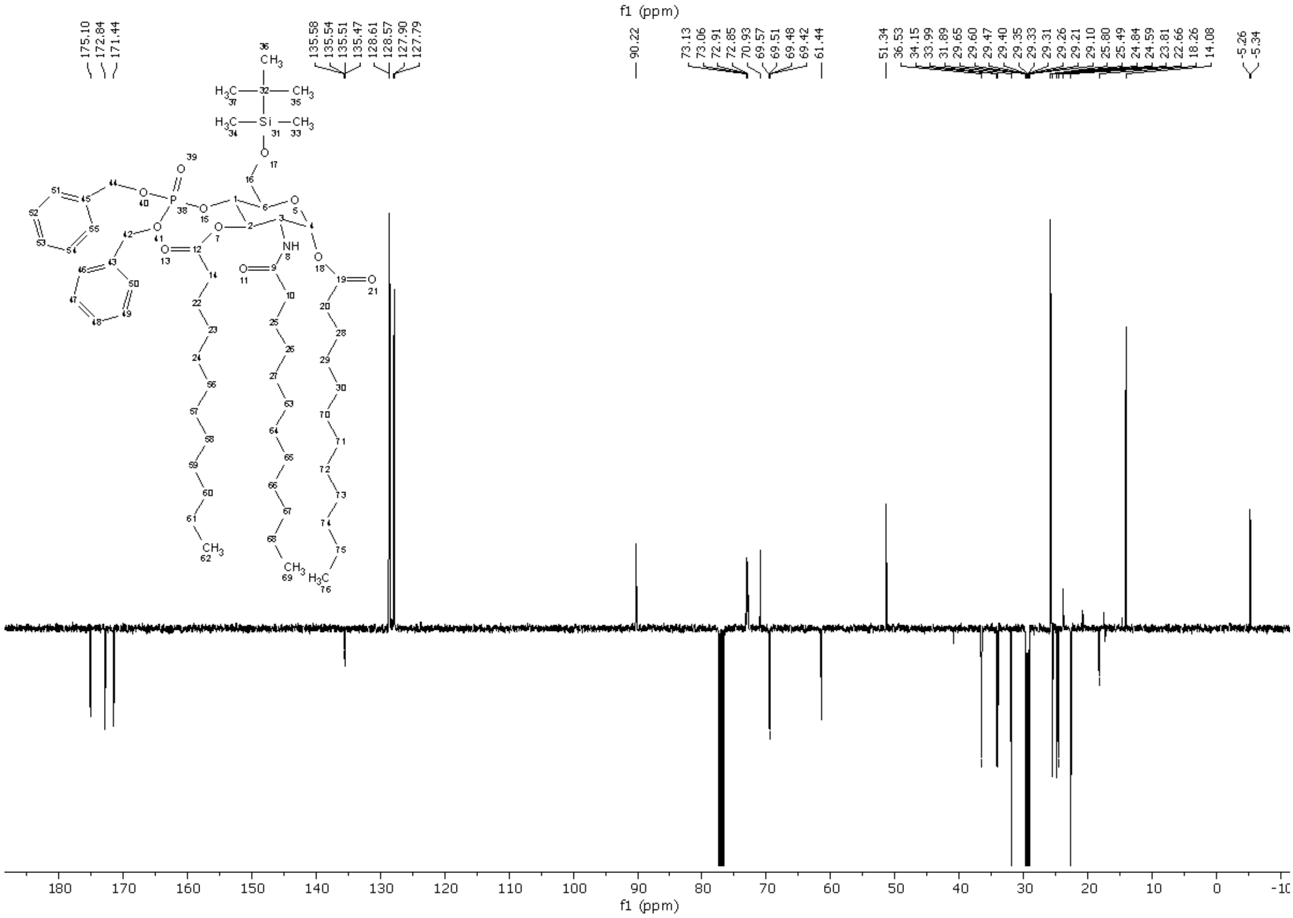

Compound 9

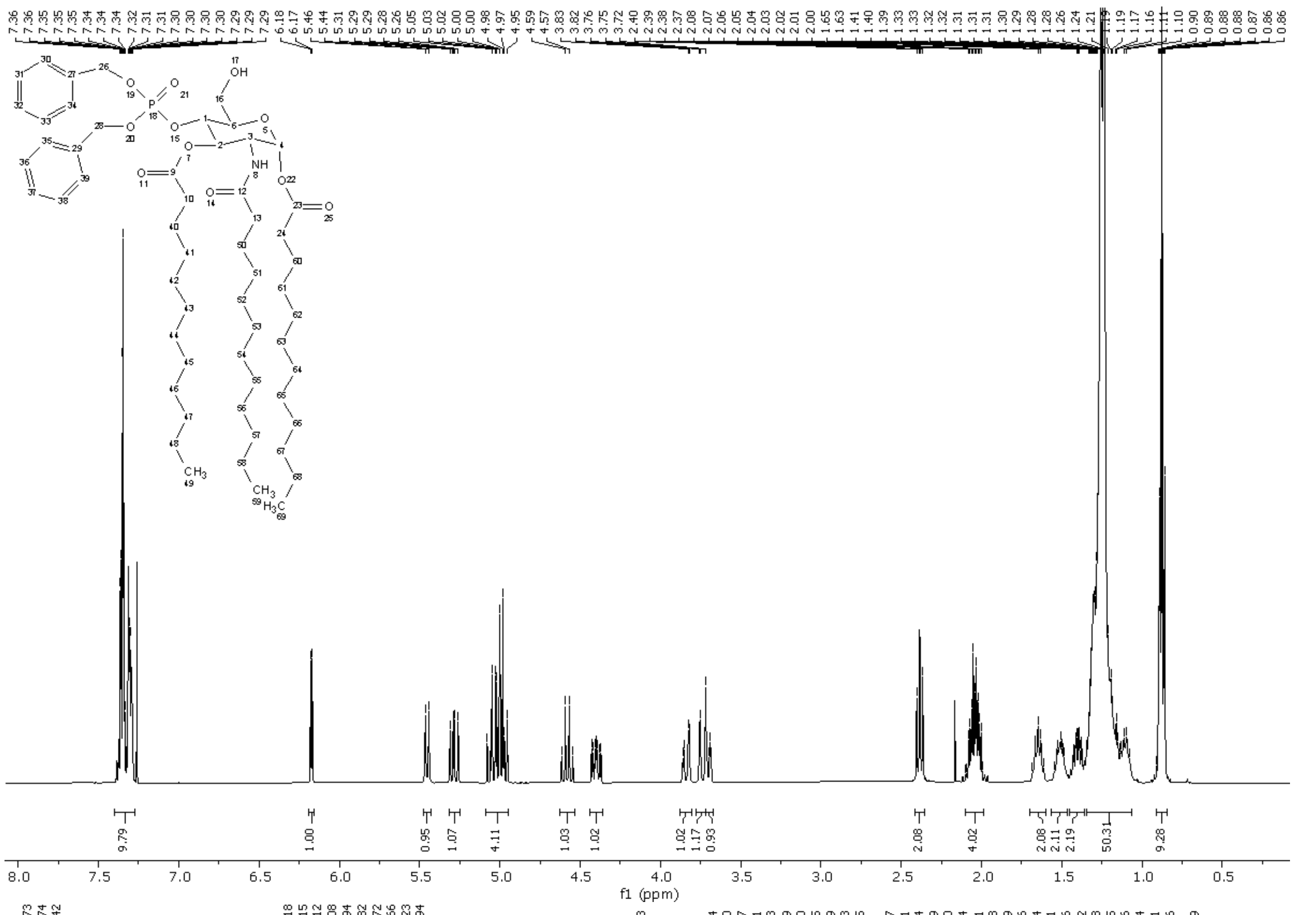

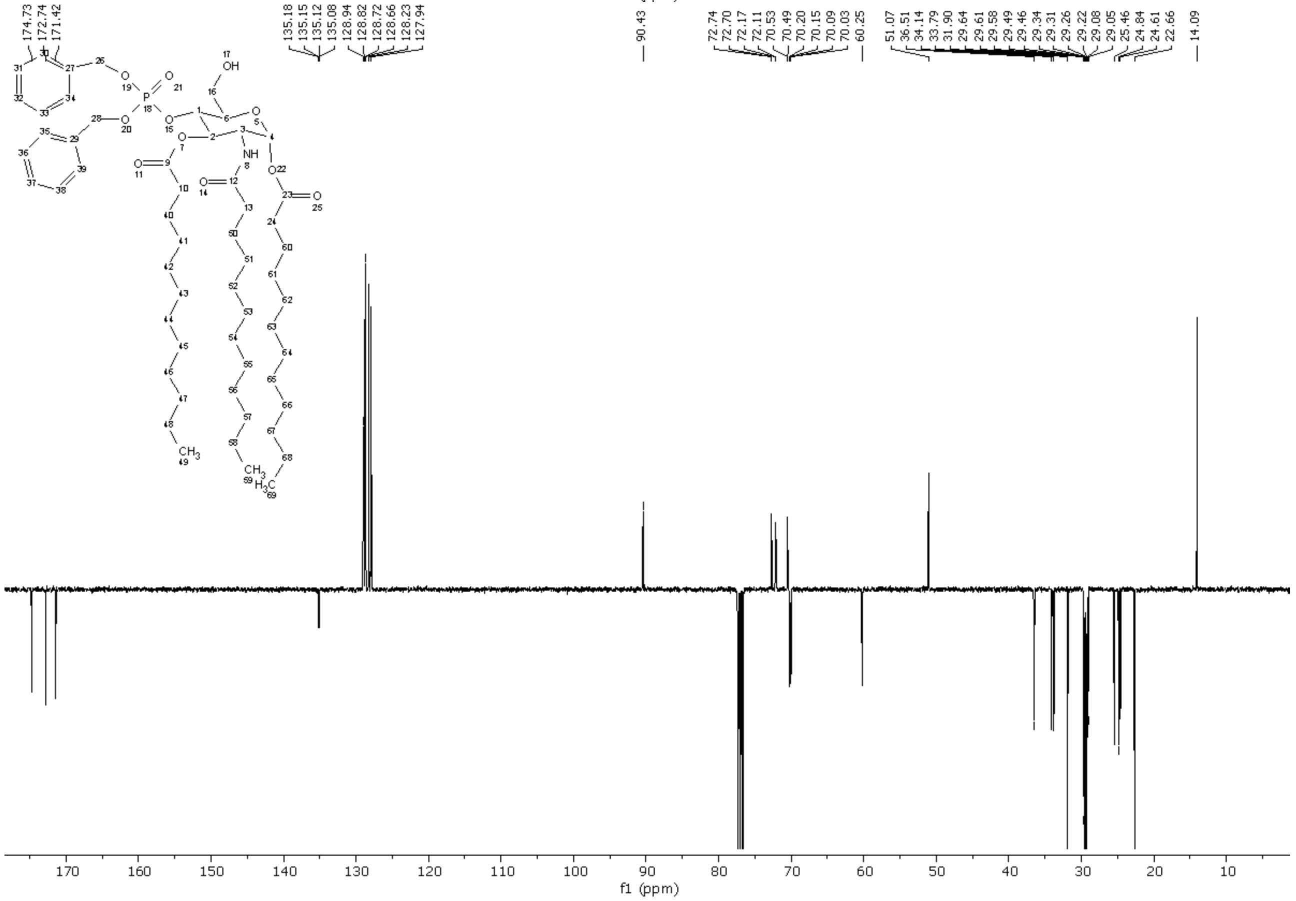

Compound 10

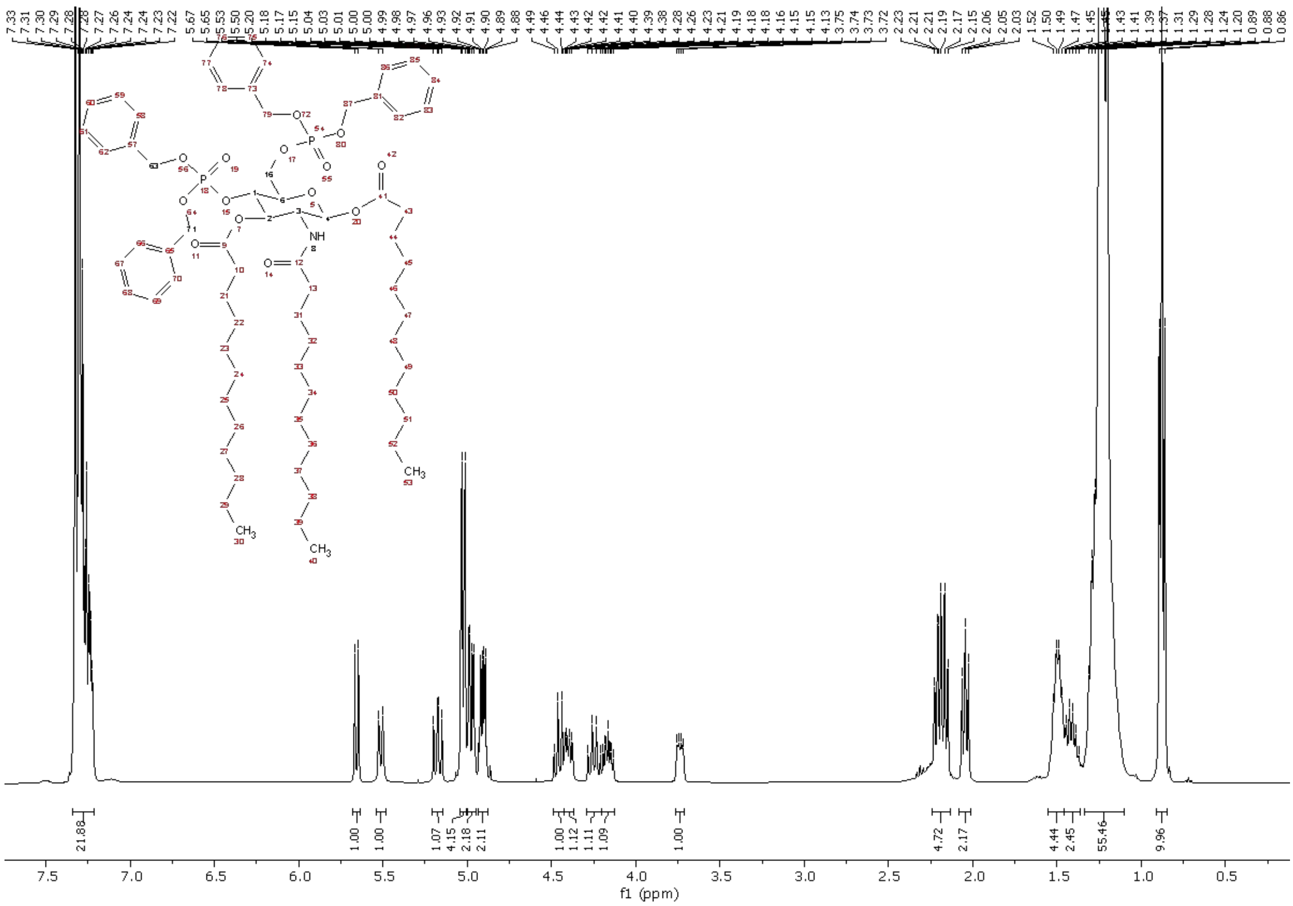

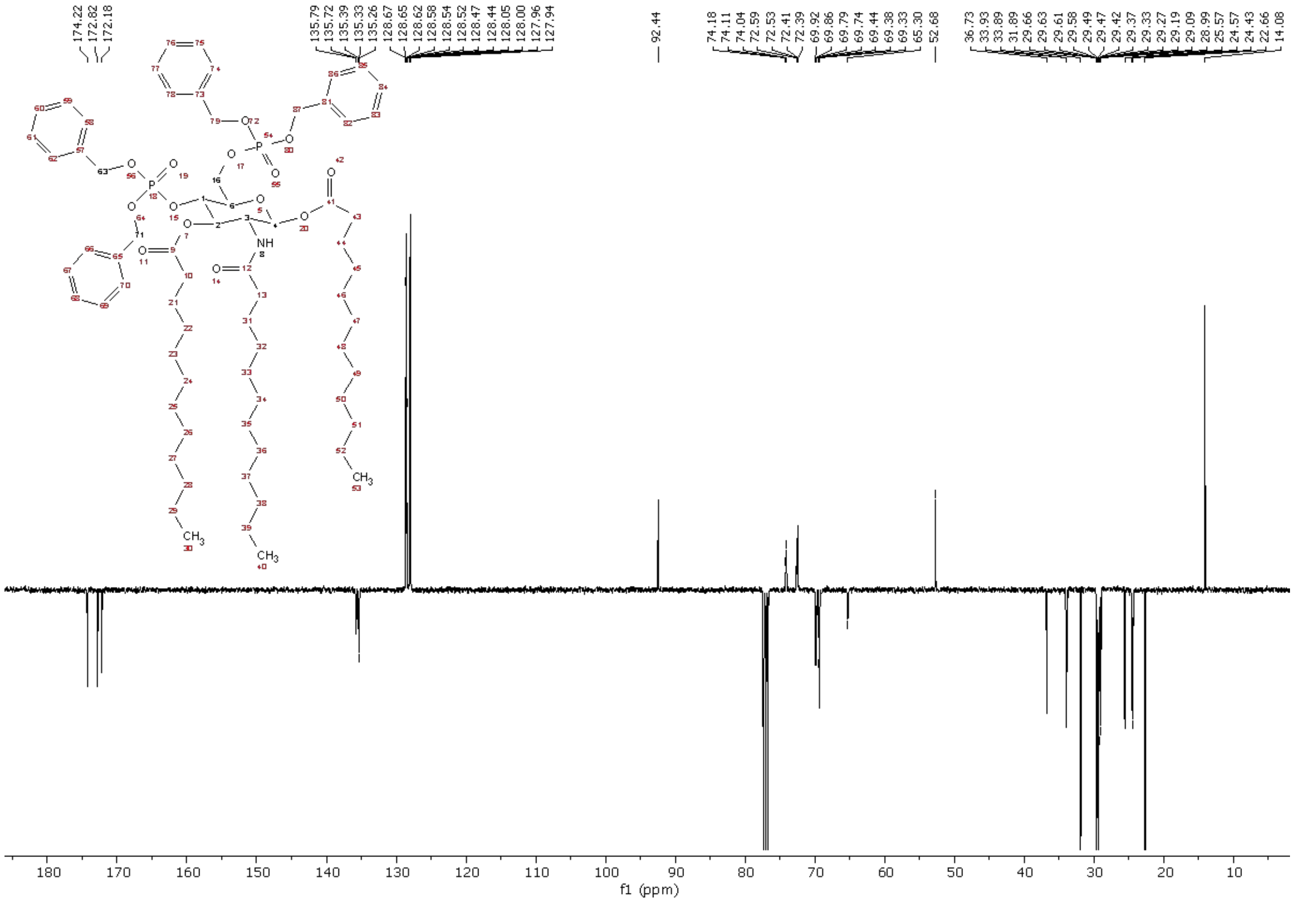

Compound FP200

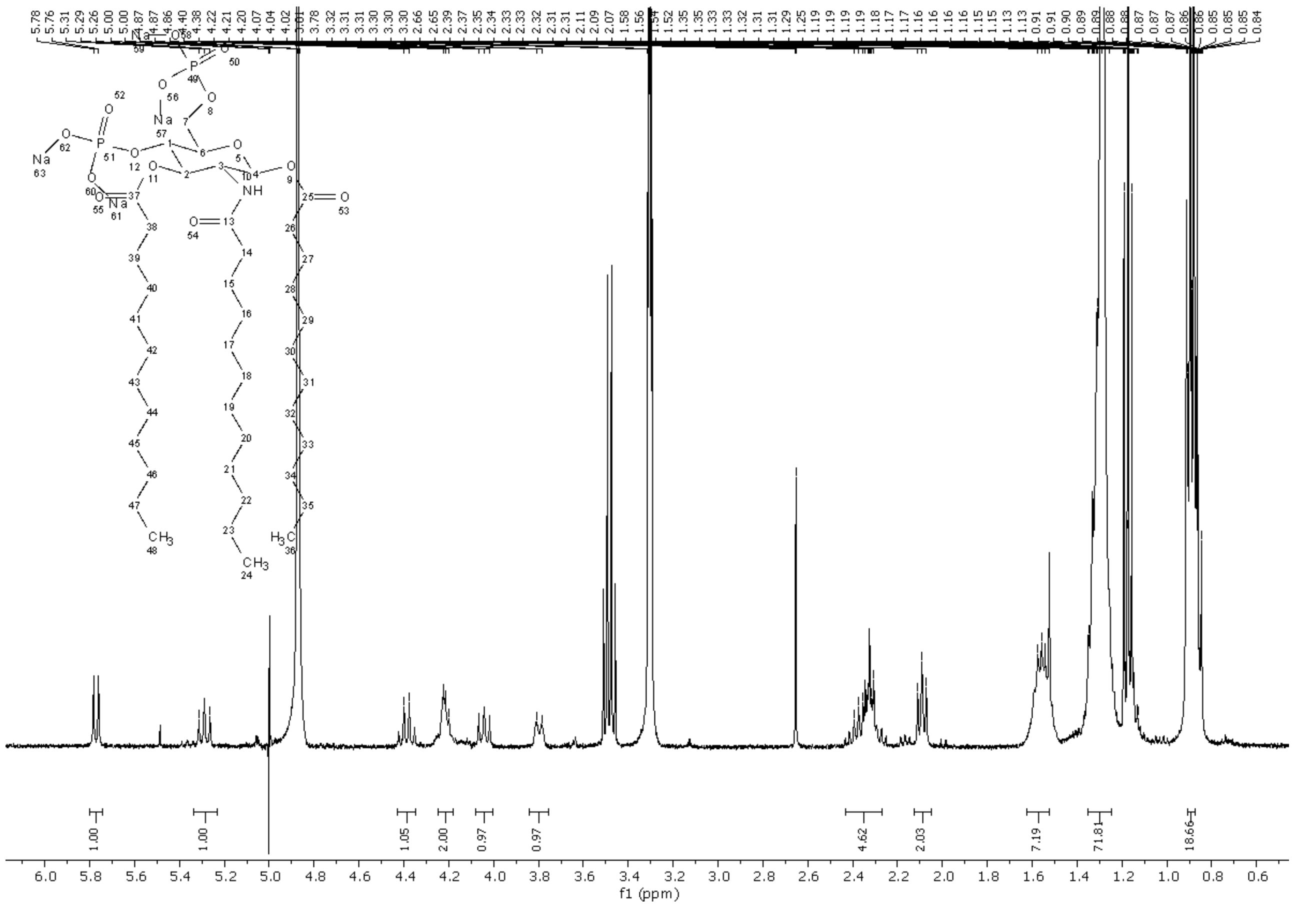

Purity Assessments

Compound FP20

	integral	n° nuclei eq.	MW		
**FP20**	1,00	1,00	850,03		
standard	0,73	9,00	172,27		
				$G_a/G_{standard}$= 60,834	
vol NMR tube (ml)		0,6			
vol analyte solution (ml)		0,6			
			q.ty (mg)	q.ty (mmoli)	[C] (mM)
standard			0,103362	0,00060000	1,000
**Weighted FP20 (mg)**	**Purity**	**q.ty (mg)**	**q.ty (mmoli)**	**$[C]_{NMR\ tube}$ (mM)**	**$[C]_{analyte}$ (mM)**
**6,58**	**95,56**	**6,288**	**0,007397**	**12,329**	**12,329**

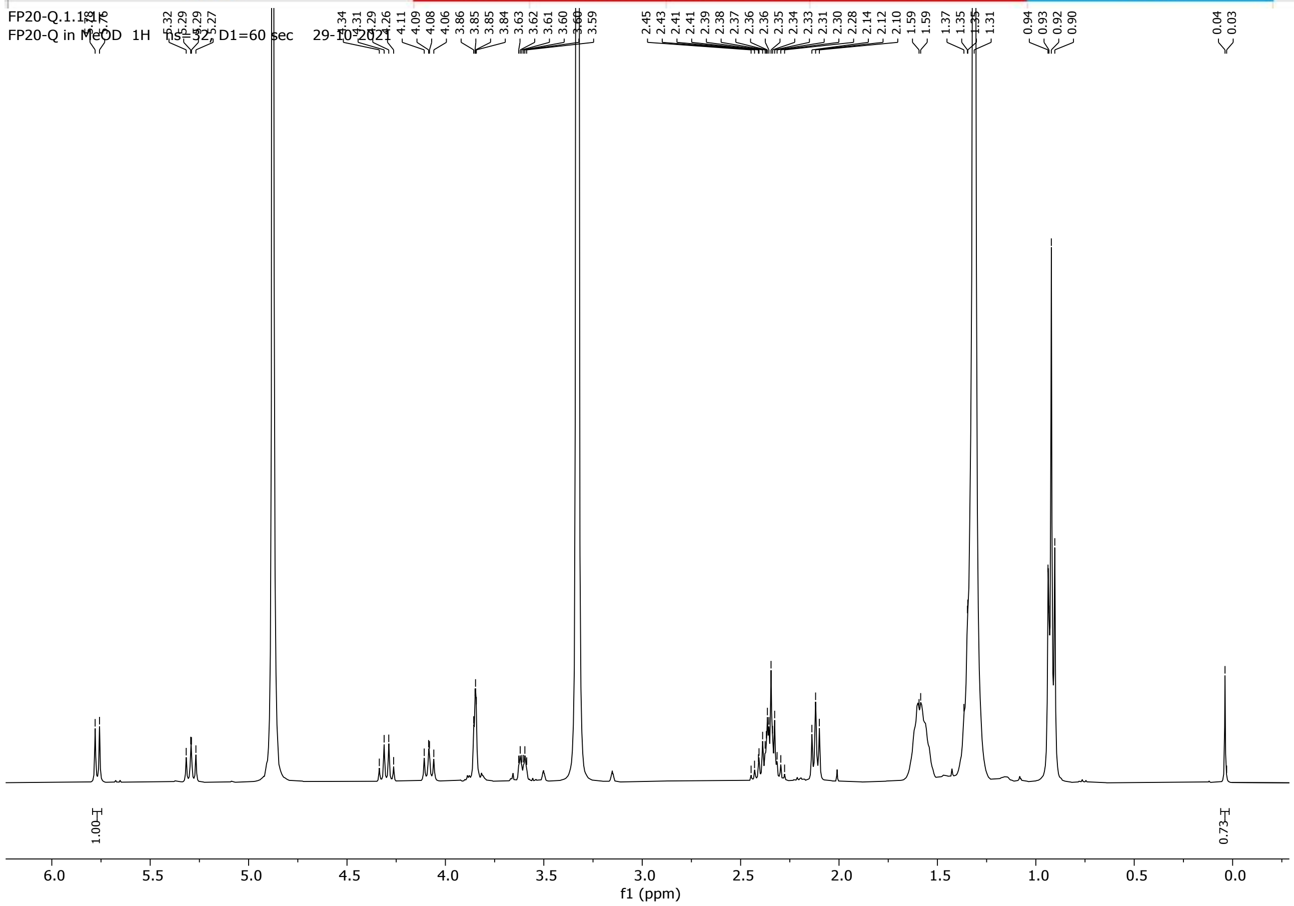

Compound FP21

	integral	n° nuclei eq.	MW		
**FP21**	1,00	1,00	906,14		
standard	0,91	9,00	172,27		
				$G_a/G_{standard}$= 52,022	
vol NMR tube (ml)		0,6			
vol starting analyte (ml)		0,6			
			q.ty (mg)	q.ty (mmoli)	[C] (mM)
standard			0,1292025	0,00075000	1,250
**Weighted FP21 (mg)**	**Purity**	**q.ty (mg)**	**q.ty (mmoli)**	**$[C]_{NMR\ tube}$ (mM)**	**$[C]_{analyte}$ (mM)**
**6,83**	**98,41**	**6,721**	**0,007418**	**12,363**	**12,363**

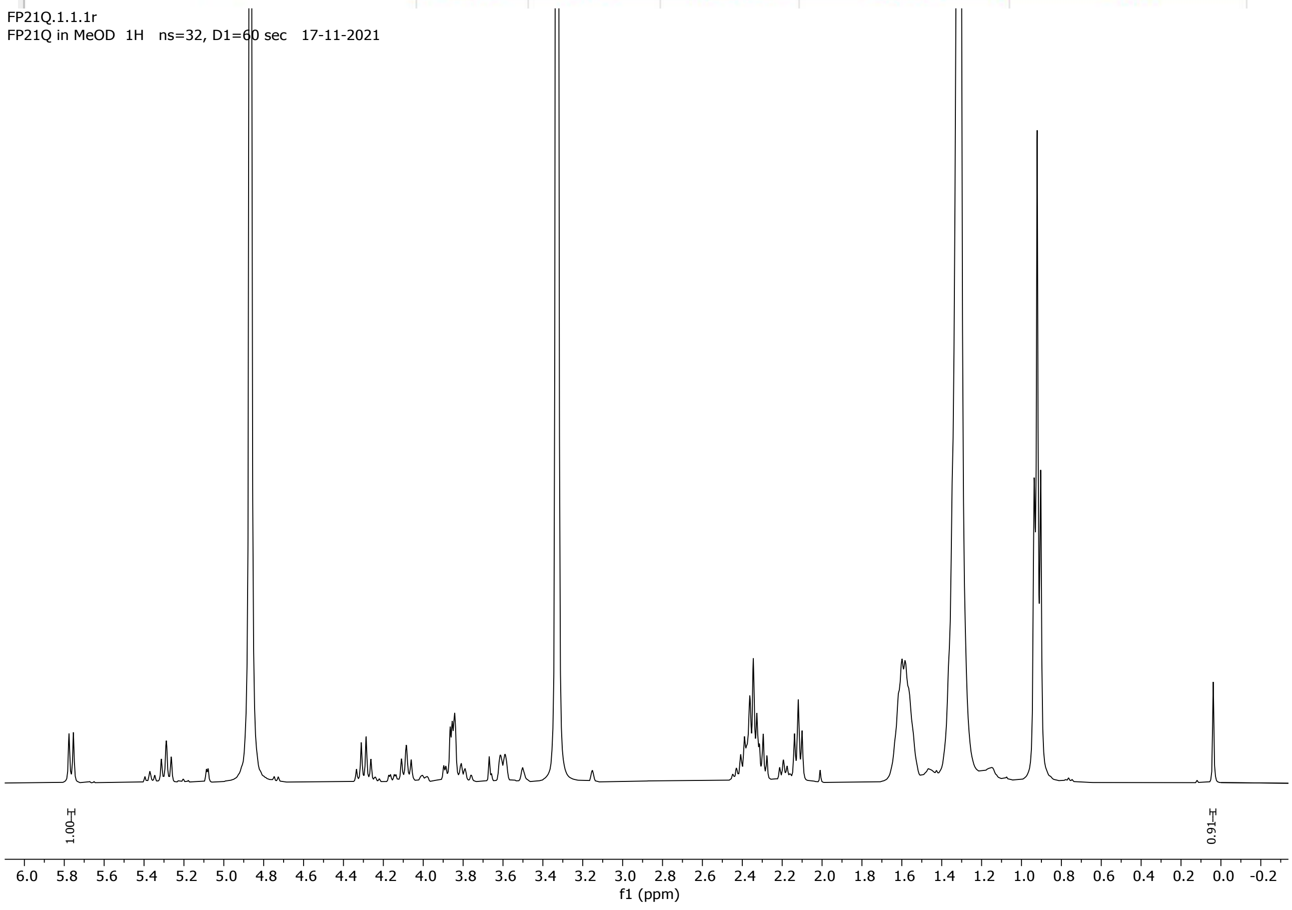

Compound FP22

	integral	n° nuclei eq.	MW		
**FP22**	1,00	1,00	765,87		
standard	0,87	9,00	172,27		
				$G_a/G_{standard}$= 45,991	
vol NMR tube (ml)		0,6			
vol starting analyte (ml)		0,6			
			q.ty (mg)	q.ty (mmoli)	[C] (mM)
standard			0,1292025	0,00075000	1,250
**Weighted FP22 (mg)**	**Purity**	**q.ty (mg)**	**q.ty (mmoli)**	**[C] $_{NMR\ tube}$ (mM)**	**[C]$_{analyte}$ (mM)**
**6,07**	**97,89**	**5,942**	**0,007759**	**12,931**	**12,931**

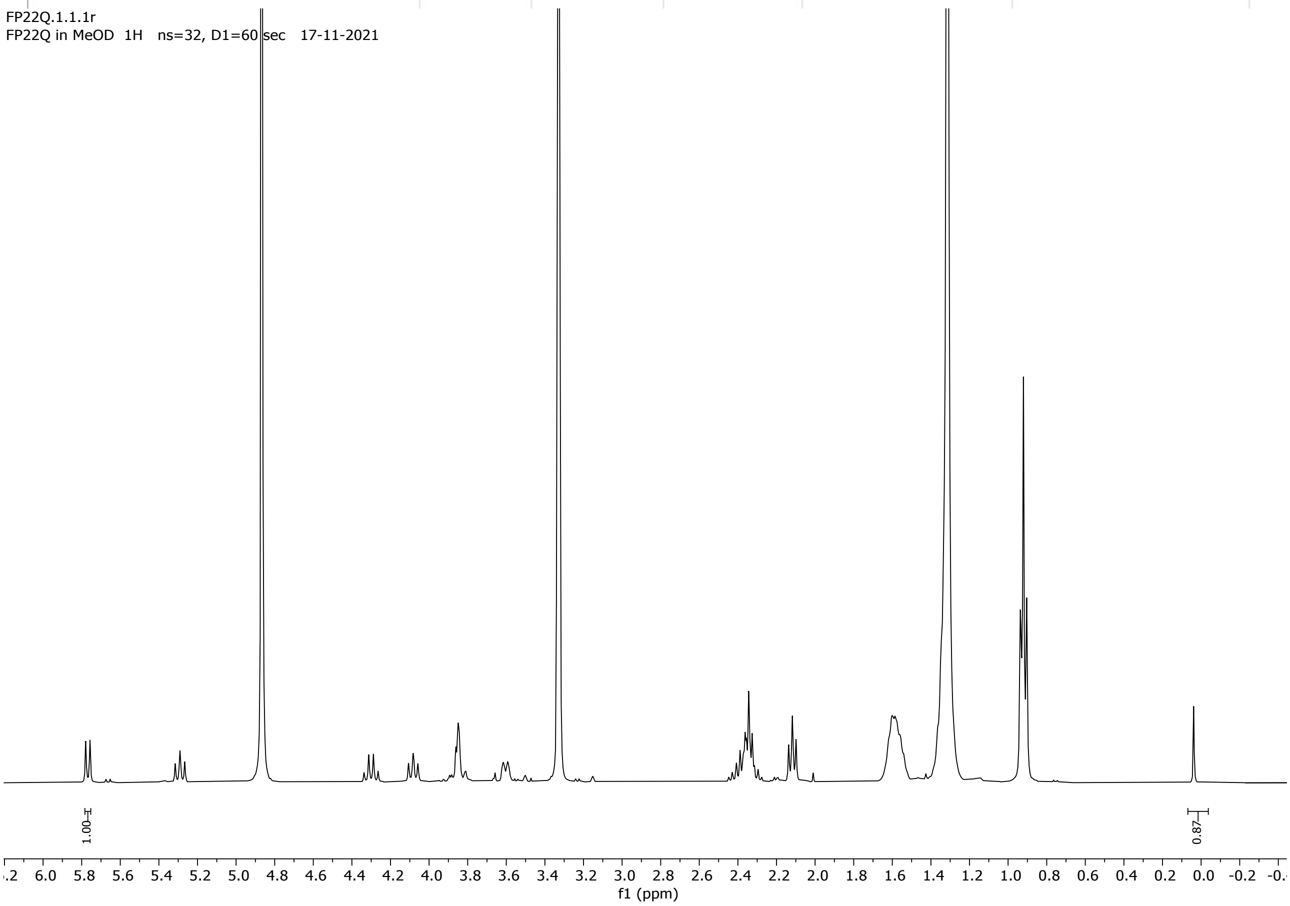

Compound FP23

	integral	n° nuclei eq.	MW		
**FP23**	1,00	1,00	934,00		
standard	1,48	9,00	172,27		
				$G_a/G_{standard}$= 32,970	
vol NMR tube (ml)		0,6			
vol analyte solution (ml)		0,6			
			q.ty (mg)	q.ty (mmoli)	[C] (mM)
standard			0,206724	0,00120000	2,000
**Weighted FP23 (mg)**	**Purity**	**q.ty (mg)**	**q.ty (mmoli)**	**$[C]_{NMR\ tube}$ (mM)**	**$[C]_{analyte}$ (mM)**
**6,88**	**99,07**	**6,816**	**0,007297**	**12,162**	**12,162**

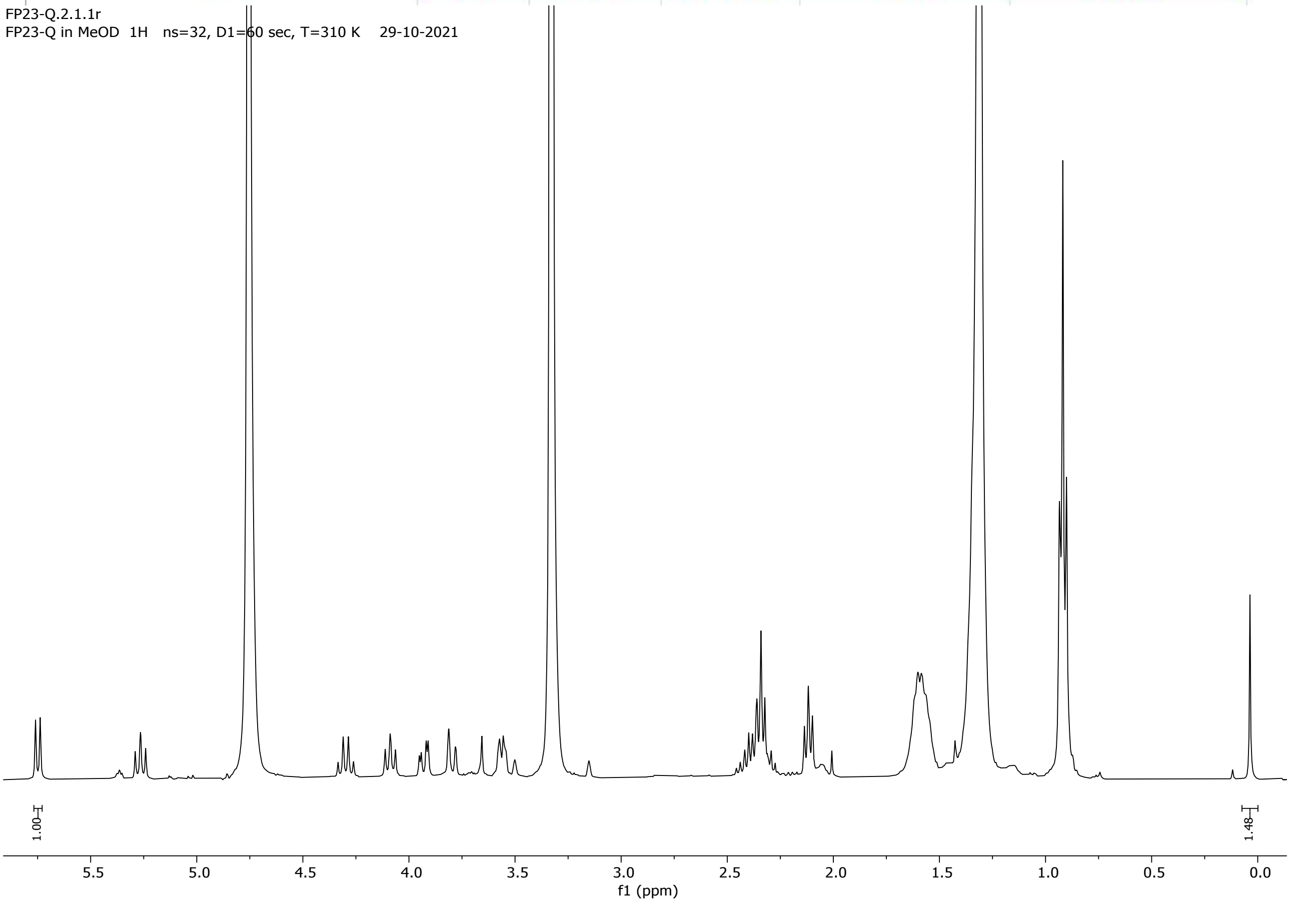

Compound FP24

	integral	n° nuclei eq.	MW		
**FP24**	1,00	1,00	793,93		
standard	0,78	9,00	172,27		
				$G_a/G_{standard}$= 53,177	
vol campione NMR (ml)		0,6			
vol campione di partenza (ml)		0,6			
			q.ty (mg)	q.ty (mmoli)	[C] (mM)
standard			0,1292025	0,00075000	1,250
**Weighted FP24 (mg)**	**Purity**	**q.ty (mg)**	**q.ty (mmoli)**	**[C] $_{NMR\ tube}$ (mM)**	**[C]$_{analyte}$ (mM)**
**6,93**	**99,14**	**6,871**	**0,008654**	**14,423**	**14,423**

FP24R.1.1.1r
FP24Q in MeOD 1H ns=32, D1=60 sec 17-11-2021

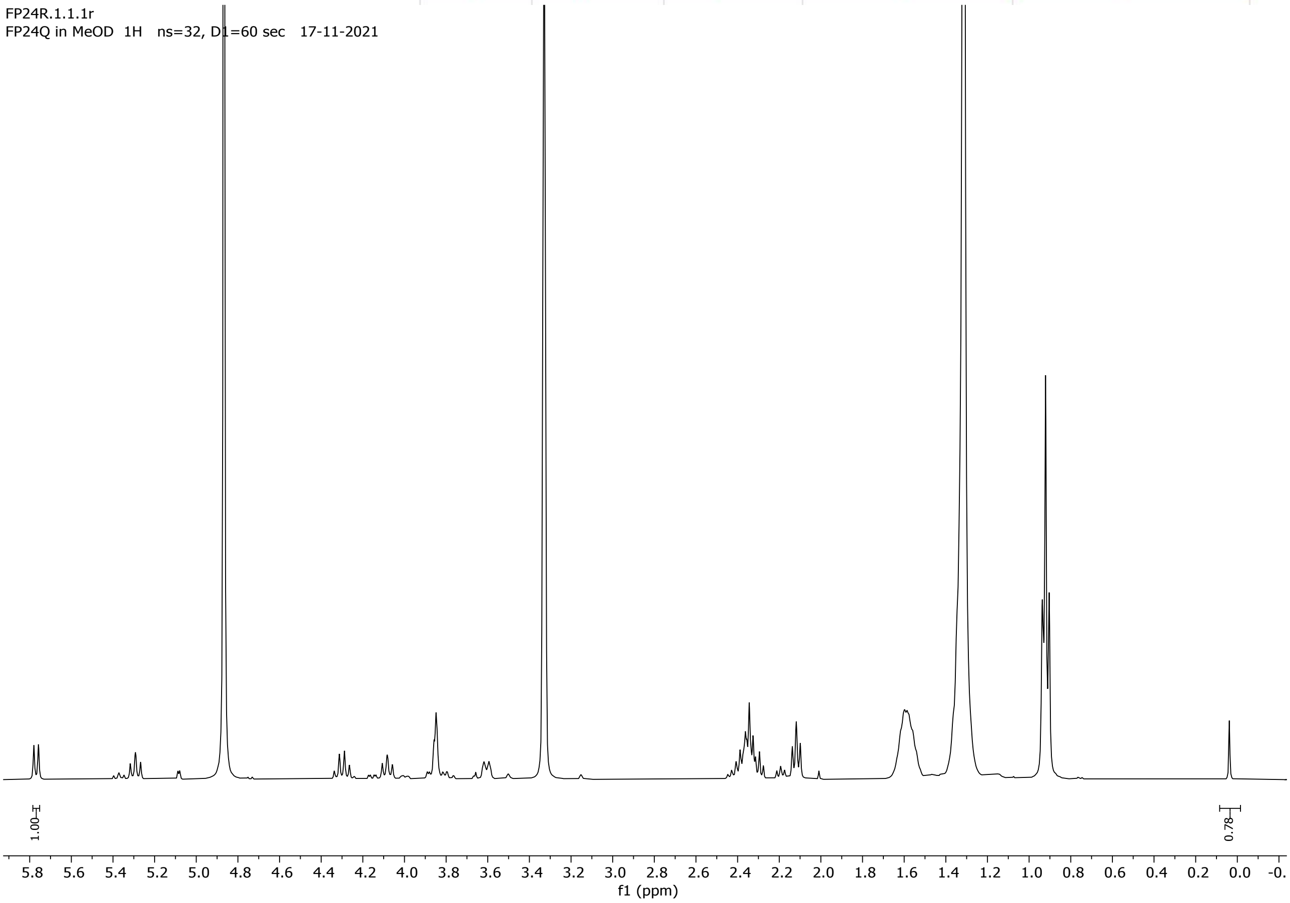

SMILES

(Also available as CSV files)

Biological Activity	TNF-α induction (pg/mL at 25 µM)	IL-1β induction (pg/mL at 25 µM)	IL-6 induction (pg/mL at 25 µM)	Antibody response to OVA immunization (AUC)
**FP20**	103 (TDM); 2511 (PBMC)	304 (TDM); 1803 (PBMC)	11963 (PBMC, not significative)	2
**FP21**	103 (TDM); 2160 (PBMC)	172 (TDM); 656 (PBMC)	5618 (PBMC, not significative)	
**FP22**	44 (TDM); 2760 (PBMC)	32 (TDM); 2022 (PBMC)	6778 (PBMC, not significative)	

SMILES	Name	SMILES
Biologically Active Compounds	FP20	O=C(CCCCCCCCCCC)O[C@H]1[C@H](OP(O[Na])(O[Na])=O)[C@@H](CO)O[C@@H](OC(CCCCCCCCCCC)=O)[C@@H]1NC(CCCCCCCCCCC)=O
	FP21	O=C(CCCCCCCCCCCCC)O[C@H]1[C@H](OP(O[Na])(O[Na])=O)[C@@H](CO)O[C@@H](OC(CCCCCCCCCCCCC)=O)[C@@H]1NC(CCCCCCCCCCC)=O
	FP22	O=C(CCCCCCCCC)O[C@H]1[C@H](OP(O[Na])(O[Na])=O)[C@@H](CO)O[C@@H](OC(CCCCCCCCC)=O)[C@@H]1NC(CCCCCCCCC)=O
	FP23	O=C(CCCCCCCCCCCCC)O[C@H]1[C@H](OP(O[Na])(O[Na])=O)[C@@H](CO)O[C@@H](OC(CCCCCCCCCCCCC)=O)[C@@H]1NC(CCCCCCCCCCCCC)=O
	FP24	O=C(CCCCCCCCC)O[C@H]1[C@H](OP(O[Na])(O[Na])=O)[C@@H](CO)O[C@@H](OC(CCCCCCCCC)=O)[C@@H]1NC(CCCCCCCCCCC)=O
	α-FP20	O=C(CCCCCCCCCCC)O[C@H]1[C@H](OP(O[Na])(O[Na])=O)[C@@H](CO)O[C@H](OC(CCCCCCCCCCC)=O)[C@@H]1NC(CCCCCCCCCCC)=O
	FP200	O=C(CCCCCCCCCCC)O[C@H]1[C@H](OP(O[Na])(O[Na])=O)[C@@H](COP(O[Na])(O[Na])=O)O[C@@H](OC(CCCCCCCCCCC)=O)[C@@H]1NC(CCCCCCCCCCC)=O
Synthetic Intermediates (Examples)	6a	OC[C@H]1OC(O)[C@H](NC(CCCCCCCCCCC)=O)[C@@H](O)[C@@H]1O
	7a	O[C@H]1[C@H](O)[C@@H](CO[Si](C)(C)C(C)(C)C)OC(O)[C@@H]1NC(CCCCCCCCCCC)=O
	8a	O=C(CCCCCCCCCCC)N[C@@H]1[C@@H](OC(CCCCCCCCCCC)=O)[C@H](O)[C@@H](CO[Si](C)(C)C(C)(C)C)O[C@H]1OC(CCCCCCCCCCC)=O
	9a	O=C(CCCCCCCCCCC)O[C@H]1[C@H](OP(OCC2=CC=CC=C2)(OCC3=CC=CC=C3)=O)[C@@H](CO[Si](C)(C)C(C)(C)C)O[C@@H](OC(CCCCCCCCCCC)=O)[C@@H]1NC(CCCCCCCCCCC)=O
	10a	O=C(CCCCCCCCCCC)O[C@H]1[C@H](OP(OCC2=CC=CC=C2)(OCC3=CC=CC=C3)=O)[C@@H](CO)O[C@@H](OC(CCCCCCCCCCC)=O)[C@@H]1NC(CCCCCCCCCCC)=O
	11a	O=C(CCCCCCCCCCC)N[C@@H]1[C@@H](OC(CCCCCCCCCCC)=O)[C@H](O)[C@@H](CO[Si](C)(C)C(C)(C)C)O[C@@H]1OC(CCCCCCCCCCC)=O
	12a	O=C(CCCCCCCCCCC)O[C@H]1[C@H](OP(OCC2=CC=CC=C2)(OCC3=CC=CC=C3)=O)[C@@H](CO[Si](C)(C)C(C)(C)C)O[C@H](OC(CCCCCCCCCCC)=O)[C@@H]1NC(CCCCCCCCCCC)=O
	13a	O=C(CCCCCCCCCCC)O[C@H]1[C@H](OP(OCC2=CC=CC=C2)(OCC3=CC=CC=C3)=O)[C@@H](CO)O[C@H](OC(CCCCCCCCCCC)=O)[C@@H]1NC(CCCCCCCCCCC)=O
	14a	O=C(CCCCCCCCCCC)O[C@H]1[C@H](OP(OCC2=CC=CC=C2)(OCC3=CC=CC=C3)=O)[C@@H](COP(OCC4=CC=CC=C4)(OCC5=CC=CC=C5)=O)O[C@@H](OC(CCCCCCCCCCC)=O)[C@@H]1NC(CCCCCCCCCCC)=O
